# Development and characterization of an in vitro model of colorectal adenocarcinoma with MDR phenotype

**DOI:** 10.1002/cam4.694

**Published:** 2016-03-25

**Authors:** Lorenzo Cinci, Cristina Luceri, Elisabetta Bigagli, Ilaria Carboni, Sara Paccosi, Astrid Parenti, Daniele Guasti, Marcella Coronnello

**Affiliations:** ^1^ Departments of Neuroscience, Psychology, Drug Research and Child Health‐NEUROFARBA, Section of Pharmacology and ToxicologyUniversity of FlorenceViale G. Pieraccini 6FlorenceItaly; ^2^ Diagnostic Genetics UnitAzienda Ospedaliero Universitaria “Careggi”Largo Brambilla 5FlorenceItaly; ^3^ Department of Health Sciences, Clinical Pharmacology and Oncology SectionUniversity of FlorenceViale G. Pieraccini 6FlorenceItaly; ^4^ Department of Experimental and Clinical Medicine, Section of Anatomy and HistologyUniversity of FlorenceLargo Brambilla 5FlorenceItaly

**Keywords:** Colorectal cancer, multi drug resistance, trascriptomics

## Abstract

The major cause of failure in cancer chemotherapy is the development of multidrug resistance (MDR), and the characterization of biological factors involved in this response to therapy is particularly needed. A doxorubicin‐resistant HCT‐8/R clone was selected from sensitive parental cells and characterized analyzing several parameters (cell cycle phase distribution, apoptotic activity, expression, distribution and functionality of the P‐gp efflux pump, the response to other chemotherapy agents, its ultrastructural features, invasiveness, and transcriptomic profile). HCT‐8/R cells showed a peculiar S phase distribution, characterized by a single pulse of proliferation, resistance to drug‐mediated apoptosis, increased expression and functionality of P‐gp and overexpression of stem cell markers (CD44 and aldehyde dehydrogenase 1A2). At the ultrastructural level, HCT‐8/R presented a greater cell volume and several intracytoplasmic vesicles respect to HCT‐8. Moreover, the resistant clone was characterized by cross resistance to other cytotoxic drugs and a greater capacity for migration and invasion, compared to parental cells. Our data reinforce the concept that the MDR phenotype in HCT‐8/R cells is multifactorial and involves multiple mechanisms, representing an interesting tool to understand the biological basis of MDR and to test strategies that overcome resistance to chemotherapy.

## Introduction

Every year, a large number of new cases of colorectal cancer are diagnosed in the world. Colorectal cancer is the third most common cancer and the fourth leading cause of cancer death worldwide 1. Surgical intervention and chemotherapy represent the first‐line strategy for the treatment of the majority of CRC patients 2. However, the clinical outcome of CRC patients is significantly limited by the de novo or acquired chemoresistance, which contributes to most cancer‐related deaths. Tumor cells in fact, can become resistant to the drugs originally used to treat them and cross resistant to other drugs with different mechanisms of action leading to the appearance of a multidrug resistance (MDR) phenotype 3.

Several mechanisms have been suggested to mediate resistance to chemotherapeutic agents in colorectal cancer cells. One of the most studied mechanisms is the high expression of the human MDR1 gene and the P‐glycoprotein (P‐gp) transporter, encoded by MDR1 4. Pgp is a drug efflux pump, which decreases the intracellular content of chemotherapeutic agents such as anthracyclines and its high expression in cancer cells is considered to be the major cause of MDR and the failure of chemotherapy 5.

In the last few years, several in vitro models of MDR in colon cancer cell lines have been developed in order to understand the biological processes underlying the onset of MDR phenotype and to test the novel therapeutic strategies to overcome this phenomenon. HCT‐8 is a cell line derived from the ileocecal portion of the right colon and represents a less frequent cancer type compared to tumors of the left colon. Chen and coworkers recently developed a HCT‐8 clone resistant to 5‐FU to evaluate the role of the tumor suppressor LIM domain‐containing protein 1 (LIMD1) in chemoresistance 6.

Besides the overexpression of efflux pumps, the MDR phenotype implicates the modulation of a large number of biological pathways, such as cell cycle, apoptosis, and DNA damage, which have not been investigated in detail.

In order to further characterize the MDR phenotype, we developed an in vitro model of HCT‐8‐resistant colorectal cancer cell and investigated the aspects of morphology, efflux balance, invasion, migration together with global gene expression profiles. Given the complexity of MDR phenomenon, a careful characterization of in vitro models could help to identify new biological processes involved in MDR and also to develop novel therapeutic strategies to overcome chemoresistance.

## Materials and Methods

### Cell culture

The HCT‐8 cell line was used as parental cell line 7 maintained in culture for many years in our laboratory. HCT‐8 cells were cultured in DMEM (Gibco Invitrogen, Carlsbad,CA) supplemented with 10% fetal bovine serum (Gibco Invitrogen), 100 U/mL penicillin–streptomycin, 1% l‐glutamine(200 mmol/L), and 4.5 g/L glucose, and grown in 5% CO2. Resistant HCT‐8 clone (HCT‐8/R) was established employing three steps selection with 10 *μ*M doxorubicin treatment for 24 h followed by culturing in drug‐free medium. Briefly, 2 × 10^5^ cells/mL were seeded in 75 cm^2^ flask with drug. After 24 h of drug exposure, the cells were washed with serum‐free medium and maintained in medium without drug. After 30 days, an individual cell clone was isolated and maintained in culture with addition of 10^−8^mol/L doxorubicin (Sigma Aldrich, Milan, Italy). All experiments were performed with cells grown in the absence of doxorubicin for at least 4d to avoid drug‐associated secondary effects.

### Cell line identification

The cancer cell identification was performed using AmpFlSTR^®^Identifiler^®^ Plus Kit (Life Technologies, Milan, Italy) in association with 3500 Genetic Analyzer and GeneMapper ID‐X Software a standard DNA STR profiling.

### CGH array

Genomic DNA was extracted by using the QiAmp tissue Kit (Qiagen, Hilden, Germany) and hybridized against Human Genome CGH 44k microarrays (Agilent Technologies, Palo Alto, CA), spanning the entire human genome at a median resolution of ~75 kb, according to the manufacturer's protocols and using lymphocytes from healthy donor as reference. Images were scanned using the Agilent DNA microarray scanner (G2505B) and extracted using the Agilent Feature Extraction Software v9.5 (Agilent Technology, Santa Clara, CA). CGH feature Extraction files were loaded into CGH Analytics 3.5.14 software (Agilent Technologies) and analyzed for aberration calls, using the human genome sequence hg17 version and analyzed for aberration calls by selecting as follows: statistical algorithm ADM‐2, centralization on, fuzzy zero on, threshold at 6.0, aberration filter set at 5 for the minimum number of probes in region and at 0.24 for the minimum absolute average log ratio for region. The Derivative Log Ratio Spread (DLRS) for the hybridization was 0.29.

### Cell cycle analysis

For cell cycle analysis, parental (HCT‐8) and resistant (HCT‐8/R) cells (1 × 10^6^ cells/mL) were fixed, at 24, 48, and 72 h after seeding, in 70% ice‐cold ethanol and stored at 4°C. Cells were stained by propidium iodide (PI) staining technique as described by Coronnello et al 8. PI‐stained cells were then analyzed on a FACSCanto flow cytometer. The fluorescence of PI was collected by a 620 nm band pass filter. Samples were gated on forward scatter versus side scatter to exclude cell debris and clumps. A minimum of 10,000 events were collected for each sample and cell cycle distribution of cells was evaluated on linear scale by Cylchred Windows 95 software (http://www.uwem.ac.uk/study/medicine/hematology/cytonetuk/documents/software.htm).

### Cytotoxicity assay

HCT‐8 and HCT‐8/R were seeded in 96‐well plates at a density of 5 × 10^3^ cells/well in 100 *μ*L of medium added of 10% FBS. After 24 h incubation at 37°C in 5% CO^2^, doxorubicin, 5‐fluorouracil and oxaliplatin (Sigma Aldrich) were added to the wells at a range of concentrations appropriate for each drug to a final volume of 200 *μ*L/well and incubated for 72 h at 37°C in 5% CO^2^. Cell growth was assessed by the colorimetric method based on [3‐(4,5‐dimethylthiazol‐2‐yl)‐5‐(3‐carboxymethoxyphenyl)‐2‐(4‐sulfophenyl)‐2H‐tetrazolium, inner salt; MTS] and an electron coupling reagent (phenazine ethosulfate; PES) (Promega Corporation, WI) The optical density of the chromogenic product was measured at 490 nm. IC_50_ values were determined graphically from relative survival curves obtained by GraphPad Prism 5 software (GraphPad, San Diego, CA). The resistance index of HCT‐8/R was calculated to determine the degree of acquired resistance to doxorubicin and to other drugs. The resistance index, expressed as ratio between IC_50_ of HCT‐8/R in comparison with IC_50_ of parental cells, was calculated according to the expression:

R = IC_50_‐resistant cell line/IC_50_‐sensitive cell line

### Transmission electron microscopy and immunoelectron microscopy

HCT‐8 and HCT‐8/R cells (10^6^cells) were fixed in 4% glutaraldehyde (Elec‐tron Microscopy Sciences, Hatfield, PA) in 0.1 mol/L cacodylate buffer, pH 7.4 for 8 h at 4°C. After fixation, for evaluation of their ultrastructure, cells were embedded in epoxy resin and routinely processed for transmission electron microscopy. For immunoelectron microscopy, after fixation, the cells were preincubated in 0.1% (v/v) Triton (Sigma Aldrich) and 1% (w/v) bovine serum albumin (Sigma Aldrich) in PBS for 15 min at room temperature. Samples were then incubated with 3% hydrogen peroxide to quench endogenous peroxidase and, successively, with primary antibody anti Human MDR‐1 P‐Glycoprotein (P‐gp) (Chemicon Millipore Corporation, Billerica, MA, USA) at final dilution of 1:20 overnight at 4°C. Immunoreaction was revealed by biotin‐conjugated, polyclonal, rabbit anti‐mouse antibody at a final dilution of 1:200 (Dako, Glostrup, Denmark), followed by incubation with streptavidin peroxidase complex (Thermo Scientific, Runcorn, Cheshire,UK) and with DAB kit Sigmafast^™^ 3,3′‐diaminobenzidine tablets (0.7 mg/mL DAB urea hydrogen peroxide mixture 1.6 mg/mLin 6 mmol/L Tris–HCl buffer (Sigma–Aldrich, St. Louis, MO, USA) for 5 min at room temperature. Successively, samples were embedded in epoxy resin and routinely processed for transmission electron microscopy. Sections cut on an ultramicrotome about 70 nm thick were examined at 80 kV in a Jeol 1010 transmission electron micro‐scope (Jeol, Tokyo, Japan).

### Percentage of polynucleolated cells

Semi‐thin sections were obtained from samples prepared for electron transmission microscopy as described above. Sections were stained with toluidine blue and observed in a Leitz microscope (Leica Microsystems MicrosystemsGmbH, Wetzlar, Germany) equipped with an image capture sys‐tem (ProgRes C10plus, Carl Zeiss, Göttingen, Germany). At least 10 photomicrographs were randomly taken from each slide with at 40× objective. Polynucleolated and total cells were counted in every microscopic filed by two observers in a blind fashion. The percentage was calculated as (polynucleolated cells/total cells)*100.

### Percentage of apoptotic cells

For evaluation of apoptotic cells, HCT‐8 and HCT‐8/R were cultured on histological slides both in the absence and in the presence of doxorubicin (2 × IC_50_, 4.10^−7^ and 2.10^−6^, respectively, for 24 h). At the end of treatments, specimens were fixed in 4% formaldehyde (freshly prepared from paraformaldehyde as normally used for experiments in optical microscopy) in 0.1 mol/L phosphate buffer, pH 7.4, for 10 min and stained with hematoxylin–eosin. The cells were observed in a microscope as described above. At least 10 photomicrographs were randomly taken from each slides at 100× objective. Apoptotic cells were identified as described in Caderni et al 9. by two observers in a blind fashion. The percentage of apoptotic cells was calculated as: (apoptotic cells/total cells)*100.

### Transcriptome analysis

Total RNA was extracted using the RNeasy Mini kit (Qiagen, Milan, Italy); RNA concentration and purity was determined by using a NanoPhotometer spectrophotometer (IMPLEN) and the RNA integrity (RIN) checked with a 2100 Bioanalyzer and a RNA 6000 Nano LabChip kit (Agilent Technologies). The labeling and hybridization steps were carried out according to the Agilent protocol (Two‐Color Microarray‐Based Gene Expression Analysis version 5.7), using a two‐color design in which HCT‐8/R cells were contrasted within parental HCT‐8.. The labeled samples were hybridized to Agilent Human GE 4 × 44K microarray in Agilent microarray chambers (G2534A) at 65° for 18 h. GE arrays were scanned using a Genepix 4000B microarray scanner at 5‐*μ*m resolution (Axon Instruments, Foster City, CA, USA). Image analysis and initial quality control were performed using the Agilent Feature Extraction Software v9.5. Differentially genes were identified by t‐test, comparing normalized red (HCT‐8/R) versus green (parental HCT‐8) signals. Pathways analysis was performed using GO‐elite version 1.2 beta (http://www.genmapp. org/go_elite).

### Real‐time PCR

RNA from HCT‐8/R and HCT‐8 cells were retrotranscribed using 100 units of SuperScriptTM II Reverse Transcriptase (Life Technologies) and 1× random examers (Roche Diagnostics, Monza, Italy). The amplification reactions were conducted in a volume of 20 *μ*L containing 1× Quantitect SYBR Green master mix (Qiagen, Valencia, CA, USA) in a 7900HT Applied Biosystems instrument. Reactions were carried out in the presence of specific primers for ATP‐binding cassette, sub‐family B, member 1, ABCB1 (5′‐AGGAAGCCAATGCCTATGACTTTA‐3′ and 5′‐CAACTGGGCCCTCTCTCTC‐3′; NM_000927), caspase 3, CASP3 (5′‐CCGTGAGGAGTTAGCGAGC‐3′ and 5′ TCCAGAGTCCATTGATTCGCT‐3′; NM_004346.3), cyclin D1, CCND1 (5′‐GATGCCAACCTCCTCAACGA‐3′ and 5′‐GGAAGCGGTCCAGGTAGTTC‐3′; NM_053056.2) or for the housekeeping gene glyceraldehyde 3‐phosphate dehydrogenase, GAPDH, used as a constitutive control for normalization.

Relative quantification of mRNA expression was carried out using the Delta Delta Ct (2^−ΔΔCT^) method (Schmittgen and Livak, 2008).

### Flow cytometry Pgp detection

P‐glycoprotein expression was evaluated by flow cytometry analysis. The cells were fixed with 70% methanol for 1 min at room temperature, then washed twice with PBS/BSA. The cells were incubated with a specific antiserum anti‐Human MDR‐1 P‐Glycoprotein P‐gp (Chemicon Millipore Corporation, Billerica, MA) used at 1 *μ*g per 5 × 10^5^ cells for 30 min at room temperature, then washed twice with PBS and detected using fluorescein isothiocyanate (FITC)‐conjugated rabbit F(ab’)2 anti‐mouse IgG (AbD Serotec, Raleigh, NC). Isotype control was used at identical concentration to the P‐glycoprotein antibody. Data (10,000 events) were acquired in the list mode on a FACScan flow cytometer (Becton‐Dickinson, San Jose, CA), equipped with a 488 nm argon laser and supported a software FACSDIVA. Fluorescence emission was collected after passing through band pass filter 530 nm for FITC. Data collected were analyzed using WinMDI 2.9 software. P‐gp grading was based on median cell fluorescence (MFI) and percentage of cells staining. P‐gp expression levels were determined by the ratio of the value of mean fluorescence intensity of the treated (MFIT) with the value of mean fluorescence intensity of control (MFIC).

### Rhodamine 123 efflux assay

The resistant subline, HCT‐8/R was tested for the activity of P‐gp protein using the fluorescent dye accumulation assay. Suspensions of parental and resistant logarithmic phase cells were obtained by trypsinization of monolayer cells. During the accumulation period, two aliquots of cells at a density of 5 × 10^5^ cell/mL were resuspended in DMEM with 10% FCS and 5 *μ*mol/L rhodamine 123 (Sigma, Milan, Italy), and incubated at 37°C in a humidified atmosphere of 5% CO_2_ for 30 min. After accumulation, efflux was initiated by sedimentation at 1200 rpm and resuspension in rhodamine‐free‐medium. The efflux was carried out at 37°C in 5% CO^2^ for 30 min with sampling time at 5, 10, 15, and 30 min. At the end of both accumulation and efflux times, cells were sedimented, washed twice in ice‐cold phosphate‐buffered saline (PBS), placed in PBS on ice, and kept in the dark until flow cytometer analysis. Samples were analyzed by FACSCanto flow cytometer (Becton‐Dickinson) equipped with a 488 nm argon laser. The fluorescence of rhodamine 123 was collected by a 530 nm band pass filter. Samples were gated on forward scatter versus side scatter to exclude cell debris and clumps. A minimum of 10,000 events were collected for each sample and analyzed by WinMDI 2.9 software (http://en.bio-soft.net/other/WinMDI.html).

### Immunocytochemistry

HCT‐8 wild‐type and HCT‐8/R cells were cultured on histological specimens. After 24 h of culture, cells were fixed in 4% formaldehyde in 0.1 mol/L phosphate buffer, pH 7.4, for 10 min. The specimens were preincubated in 0.1% (v/v) Triton (Sigma Aldrich) and 1% (w/v) bovine serum albumin (Sigma Aldrich) in PBS for 15 min at room temperature. The cells were incubated with primary antibody anti‐Human MDR‐1 P‐Glycoprotein P‐gP (Chemicon Millipore Corporation) at final dilution of 1:20 overnight at 4°C. Immunoreaction was revealed by using the secondary antibody Alexa Fluor 488 rabbit anti‐mouse (Invitrogen, San Diego, CA) 1:200 for 2 h at room temperature. At least 10 microscopic fields were randomly taken for each specimen. Measurement of the optical density of P‐gp‐immunosatined cells was carried out using the ImageJ 1.33 free‐share image analysis software (http://rsb.info.nih.gov/ij).

### Doxorubicin uptake

For evaluation of intracellular content of doxorubicin, HCT‐8 and HCT‐8/R were cultured on histological slides and treated for 2 h with 10^−5^mol/L doxorubicin. At the end of the treatment, slides were fixed as described above and observed by fluorescence microscopy (Labophot‐2 Nikon, Japan). Ten photomicrographs were randomly taken for each sample and fluorescence was measured using ImageJ 1.33 image analysis software (http://rsb.info.nih.gov/ij).

### Cell migration and cell invasion

A modified Boyden chamber (48‐multiwell plates; Neuroprobe) was used 10, 11. Polyvinyl pyrrolidone‐free polycarbonate filters, 5 *μ*m pore size, were coated with 100 *μ*g/mL collagen type I and 10 *μ*g/mL fibronectin for migration assay and with Matrigel, 0.5 mg/mL (Becton Dickinson, Bedford, MA), for cell invasion assay. Medium without serum (control) or supplemented with 5% FCS, was added to the lower wells, while 12.5 × 10^3^ cells were seeded into the upper wells and incubated at 37°C for 7 h for cell migration, or 24 h for cell invasion. Methanol‐fixed cells were stained with Diff‐Quik (Dade Behring, Dudingen, CH) and cell migration/invasion was measured by microscopic evaluation of the number of cells moved across the filter, in 10 randomly selected fields at magnification 400×. Each experimental point was measured in triplicate.

## Results

### Cell line identification

The DNA STR profiling (13 markers + Amelogenin, AMEL) matched with the HCT‐8 [HRT‐18] ATCC^®^ CCL‐244^™^ STR with a percent match (EV value) of 0.94. The only mismatch was at the AMEL Y locus, the STR profiling in fact, falsely genotyped our cancer cell line as obtained from a female donor.

### CGH array

HCT8 cells were analyzed by a‐CGH, contrasted with a sample of undamaged DNA from peripheral blood lymphocytes. We found only few aberrations on chromosome 6, and a large deletion on the Y(p)11.2 region encompassing the Amelogenin locus, as shown in Figure S1.

### Cell cycle analysis

In Table 1 we reported the percentage of cells in the cell cycle phases. This analysis highlighted that HCT‐8 cells have the same pattern of cell cycle distribution in the three time points analyzed. The HCT‐8/R showed a small percentage of S‐phase cells both after 24 and 72 h. Contrariwise, after 48 h, the resistant clone showed a higher percentage of S‐phase cells (44.1%). These data were concordant with doubling times of HCT‐8 and HCT‐8/R cells, 27 and 33 h, respectively.

**Table 1:** Distribution of HCT‐8 in cell cycle phases. Values represent the percentage of cells in the individual phases for increasing time point from seeding

	Percentage of cells								
	G_0_G_1_			S			G_2_M		
	24 h	48 h	72 h	24 h	48 h	72 h	24 h	48 h	72 h
HCT‐8	57.5	58.8	57.0	31.3	35.6	37.1	11.2	5.6	5.9
HCT‐8/R	64.8	50.4	65.7	25.6	44.1	30.7	9.6	5.5	3.6

### Cross‐resistance profiles

The sensitivity of both HCT‐8 lines to a range of chemotherapeutic agents was determined. The IC_50_ values for doxorubicin in HCT‐8/S and HCT‐8/R were 47 ± 1.2 and 478 ± 5.35 nmol/L, respectively (Resistance Fold (RF): 10.2). The resistant clone showed a rather high resistance even to 5‐FU (IC_50_ HCT‐8/S 2.01 ± 0.15; IC_50_ HCT‐8/R 12.72 ± 0.25; RF: 6.4). HCT‐8/R showed no resistance to oxaliplatin.

### Transcriptome analysis

Among the 14,177 probes that passed the quality‐control step, 935 (6.6%) genes were significantly up‐regulated and 765 (4.5%) down‐regulated in HCT‐8/R cells compared to the HCT‐8 parental cells, with a fold change of at least ±2 (Table S1). The most up‐regulated gene in HCT‐8/R cells was the carbonyl reductase 1 (CBR1), CD44, several families of aldehyde dehydrogenase, and three members of the carbonic anhydrase gene family (CA2, CA8, and CA13) were also overexpressed. The gene for P‐gp, MDR‐1, was up‐regulated 34‐fold in HCT‐8/R compared to the parental HCT‐8 cell line. It is interesting to note that between down‐regulated genes we found those related to cell cycle such as CCND1 (cyclin D1) and CHES1 (checkpoint suppressor 1), RRP22 (RAS related on chromosome 22) and DRAM (damage‐regulated autophagy modulator) involved in apoptotic pathway. Pathways analysis confirmed the down‐regulation of the cell cycle and found an up‐regulation of the Notch signaling pathway (Table S2).

### q‐PCR

Q‐PCR confirmed the significant up‐regulation of ABCB1 and the down‐regulation of cyclin D1 in HCT8/R cells; CASP3 gene was selected as an example of differentially expressed gene with a relatively low fold change (−1.68); however, q‐PCR emphasizes its down‐regulation in HCT‐8/R compared to the parental cells (−5 fold).

### Percentage of polynucleolated cells

**Figure 1:**
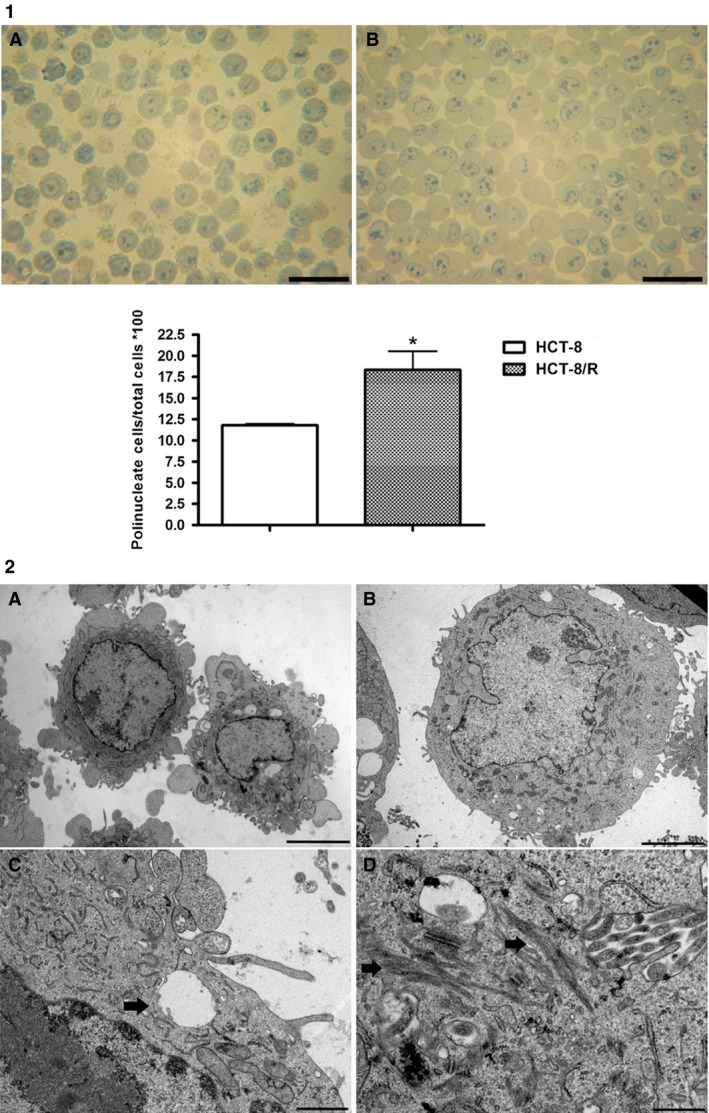
Panel 1, presence of polynucleolate cells in HCT‐8(A) and HCT‐8/R (B) evaluated in semi‐thin sections stained with toluidine blue. The percentage of polynucleolate cells are expressed in the graf. *P < 0.05, scale bar = 50 *μ*m. (ten microscopic fields analyzed with an average of 100 cells; total number of analyzed cells: 1000) Panel 2, ultrastructural images of HCT‐8 and HCT‐8/R. Panel A shows HCT‐8 cells (×6000; scale bar 5 *μ*m). Panel B shows HCT‐8/R (×6000; scale bar 5 *μ*m). Panel C higher magnification of a HCT‐8/R cell that shows a vesicular body in the cytoplasm (×20,000; scale bar 1 *μ*m). Panel D shows several cytokeratin fibers in HCT‐8/R cells (×20,000; scale bar 1 *μ*m).

The morphological analysis of semi‐thin sections has highlighted a significant increase of the number of polynucleolated cells in HCT‐8/R with respect to HCT‐8 expressed as percentage of polynucleolated cells/microscopic field (Fig. 1 Panel 1).

### Ultrastructural analysis

Ultrastructural analysis determined that resistant cells had a larger volume than sensitive cells. HCT‐8/R showed a more globular appearance with a lower number of evaginations of the cytoplasm and a high ratio nucleus/cytoplasm. This analysis demonstrated a more complicated ultrastructural organization in HCT‐8/R cells (Fig. 1). In fact, we observed an increased number of fibers, probably to be interpreted as cytokeratin. HCT‐8/R cells showed many multivesicular bodies positioned near the plasmatic membrane.

### Percentage of apoptotic cells

**Figure 2:**
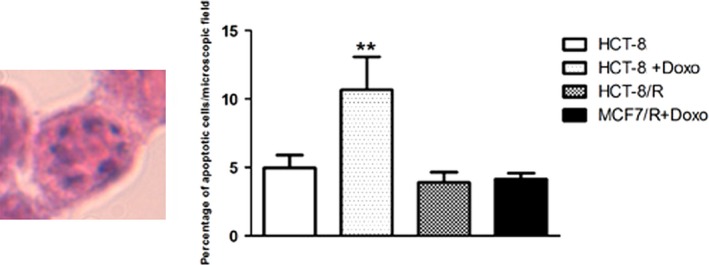
Evaluation of percentage of apoptotic cells in HCT‐8 and HCT‐8/R, in the presence and in the absence of doxorubicin. The digitalized image on the left shows an illustrative cell characterized by apoptotic appearance (presence of round or oval nuclear fragments *apoptotic bodies*). The percentage of apoptotic cells are illustrated in the graph on the right. **P < 0.01.

The percentage of apoptotic cells was evaluated in each microscopic field. Apoptotic cells were recognized by the presence of apoptotic appearance of nucleus (Fig. 2) in HCT‐8 and HCT‐8/R stained with hematoxylin–eosin. No difference was found between the percentage of apoptotic cells in HCT‐8 and HCT‐8/R in physiological conditions. The treatment with Doxorubicin (2 × IC50 for 24 h) induced a significant increase of apoptotic cells in HCT‐8 but not in HCT‐8/R cells.

### Flow cytometer evaluation of P‐gp expression and functionality

**Figure 3:**
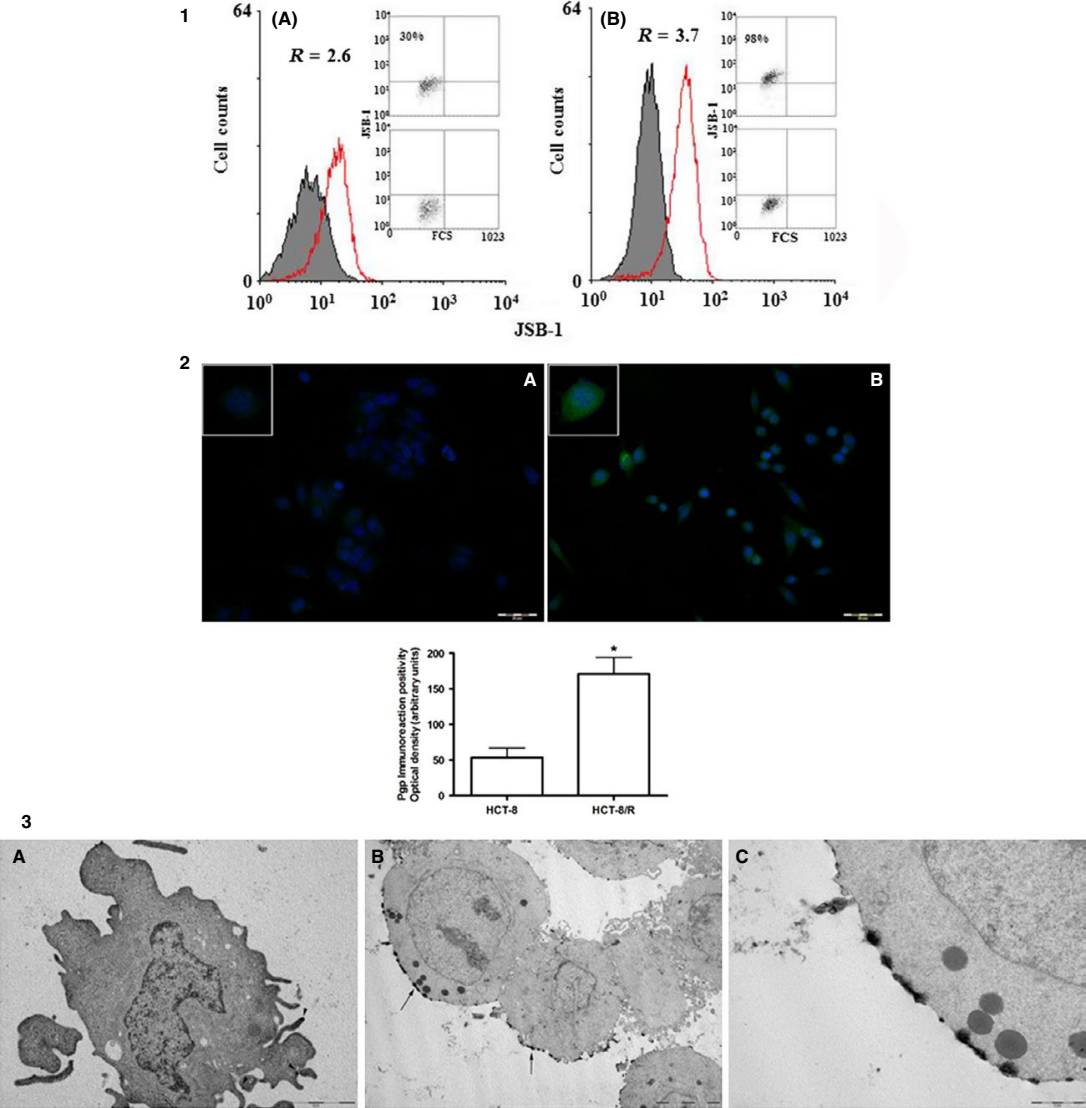
Panel 1 shows the expression of the Pgp *MDR‐1* gene product in HCT‐8 (A) and HCT‐8/R (B) cells. R = ratio between MFI of treated sample and isotype control Percentage of cells staining was also reported. Panel 2: immunocytochemistry of immunostained cells with anti‐Pgp antibody. The upper panel shows the immunoreaction positivity in HCT‐8 (panel A) and HCT‐8/R (panel B). Inserts show higher magnification of illustrative cells in which is possible to evaluate the intensity and distribution of immunolabeling. The quantitative results of densitometry are given in the graph below. *P < 0.05 (ten microscopic fields analyzed with an average of 100 cells; total number of analyzed cells: 1000) Panel 3: immunoelectron micrographs of HCT‐8 immunostained with anti‐Pgp antibody. Panel A: HCT‐8, immunoreaction positivity is visible only in a few portions of cytoplasmic membrane (black arrow heads), ×15,000. Panel B: HCT‐8/R, immunoreaction positivity appears intense and distributed in large parts of the cytoplasmic membrane, ×5000. Panel C: higher magnification of panel B, ×20,000.

**Figure 4:**
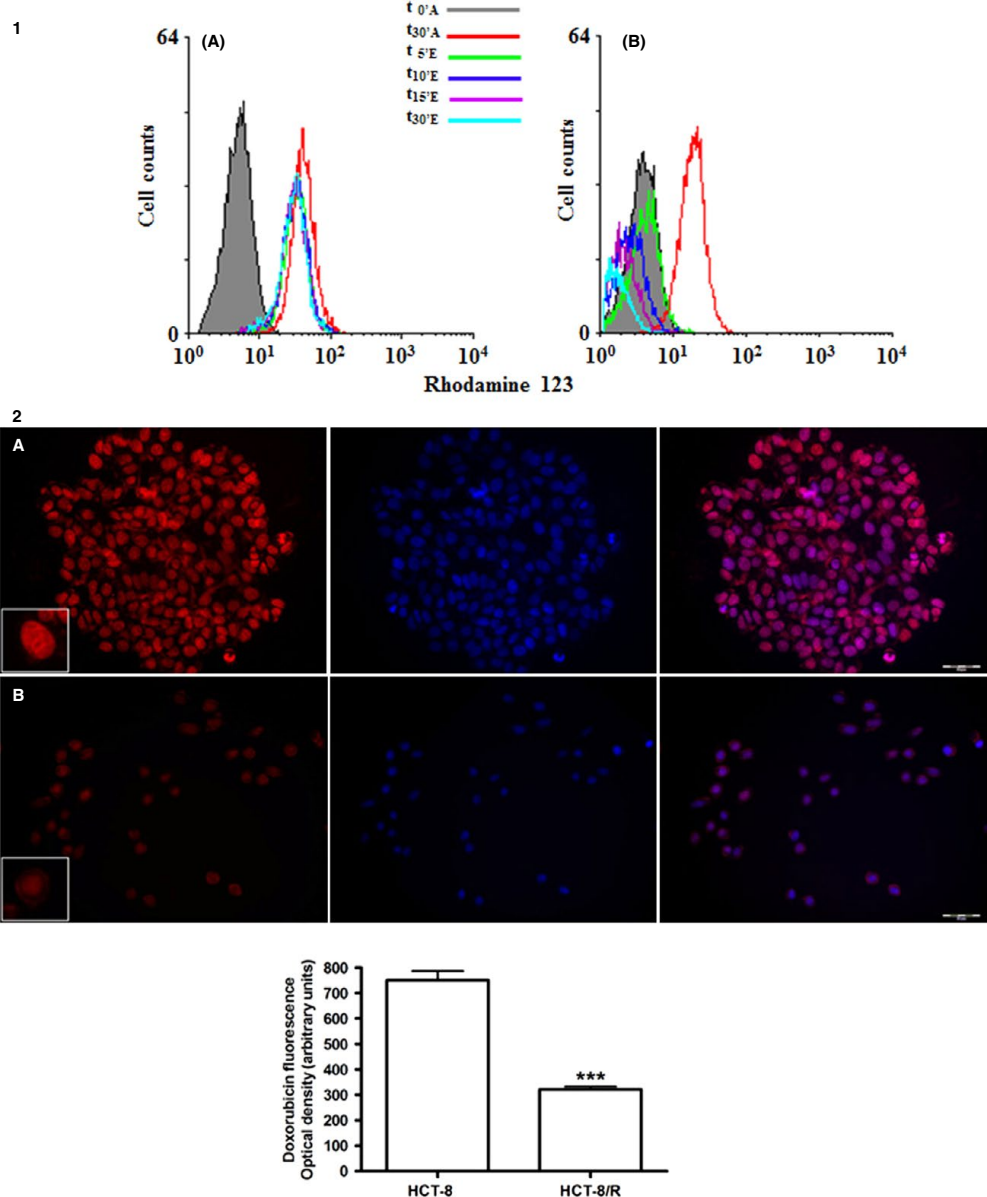
Panel 1 shows flow cytometry histograms of the intracellular rhodamine‐123 accumulation and efflux: (A) parental HCT‐8 cell line; (B) resistant HCT‐8 subline. Panel 2: doxorubicin uptake and distribution evaluated by autofluorescence. The upper panel shows doxorubicin presence and distribution in HCT‐8 (A) and HCT‐8/R (B). The first image of each row shows the doxorubicin autofluorescence, the central image shows DAPI staining and the third images shows the merge. The quantitative results are given in the graph below. ***P < 0.001 (ten microscopic fields analyzed with an average of 100 cells; total number of analyzed cells: 1000).

P‐gp expression and activity were evaluated by flow cytometer in HCT‐8 and HCT‐8/R. The sensitive cells contained 30% of P‐gp‐positive cells (Fig. 3). In HCT‐8/R cells, the percentage of P‐gp‐positive cells was 98%. P‐gp activity was evaluated by rhodamine‐123 assay. Intracellular rhodamine fluorescence was measured by flow cytometric analysis. The uptake data were collected after 30 min of incubation with rhodamine 123 and efflux results were obtained after 30 min in rhodamine‐free medium. HCT‐8/R had a reduced accumulation of rhodamine 123 with respect to HCT‐8 cells. In parallel, in the resistant clone, we observed a greater efflux of drug highlighted by the small amount of rhodamine remaining in the cells after the efflux period (Fig. 4).

### Immunocytochemical analysis for Pgp

The presence of P‐gp in HCT‐8 cells was evaluated with an immunocytochemical reaction carried out using a specific antiserum. HCT‐8 showed a weak labeling. The immunoreactivity was significantly higher in HCT‐8/R cells, in which the labeling appeared evident and distributed over the entire cytoplasmic membrane. Quantification carried out by densitometry, showed a significant increase of fluorescence levels in HCT‐8/R respect to the sensitive clone (Fig. 3).

### Immunoelectron microscopy for P‐gp

The presence and the distribution of P‐gp were evaluated also by a pre‐embedding immunoelectron technique using a specific antiserum. In HCT‐8, the labeling was evident only in limited portions of the plasma membrane. On the contrary, HCT‐8/R showed an intense labeling distributed in a cluster along rather extended portions of cell membrane (Fig. 3). The labeling was absent in cell membrane helpings involved in intercellular contacts.

### Cellular doxorubicin uptake

In HCT‐8, the autofluorescence of the drug appeared more evident respect to resistant cells. In both clones, doxorubicin was distributed mainly inside the nucleus, but in HCT‐8/R, the labeling was distributed along the cytoplasmic membrane (Fig. 4). Quantitative analysis showed a significant decrease of doxorubicin content in the resistant clone.

### Cell migration and invasion

**Figure 5:**
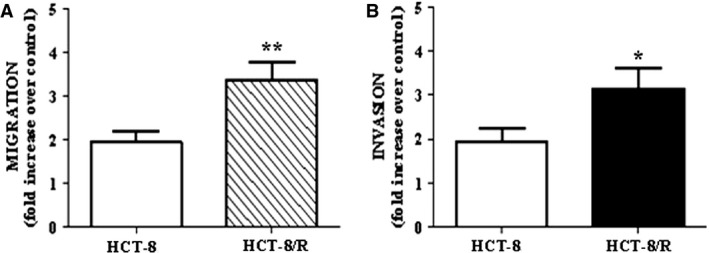
Functional characterization of HCT‐8/R cells. Cells were let to migrate (A) or to invade (B) in response to 5% FCS medium. Results are reported as increase over control (1% FCS medium). *P < 0.05, **P < 0.01 versus parental cells.

Under control condition (1% FCS medium), both HCT‐8 and HCT‐8/R cells were able to migrate, the former being more active (54 ± 3.9 and 27 ± 3.2 migrated cells for HCT‐8/S and HCT‐8/R, respectively). When cells were stimulated to migrate in response to 5% FCS, resistant cell migration was significantly higher than that of parental cells (Fig. 5). Comparable results were obtained with invasion experiments, in which HCT‐8/R had a more aggressive invasive phenotype, characterized by a significantly higher cell migration compared to parental cells (3.14 ± 0.4 vs. 1.93 ± 0.3 fold increase, for doxorubicin‐resistant and parental cells, respectively, Fig. 5).

## Discussion

Colorectal cancer (CRC) is a serious public health problem, with more than one million new cases and over a half million deaths worldwide each year 12. Multidrug resistance (MDR) profoundly limits the effectiveness of currently used chemotherapeutic regimens for many human malignancies including CRC 13, 14.

The MDR phenotype is a complex phenomenon since numerous factors, beyond the up‐regulation of drug efflux pumps such as P‐gp, affect drug sensitivity. Some of them have been reported such as drug activation and inactivation mechanisms, alterations in drug targets, DNA methylation, processing of drug‐induced damage, and evasion of apoptosis 15. In this view, the strategy to revert chemoresistance by solely inhibiting P‐gp might be insufficient and/or bypassed by the activation of alternative biological pathways. Consequently, a multitarget approach to overcome chemoresistance might have more chances of success. Thus, the aim of our work was to develop and deeply characterize a colon cancer cell model displaying MDR to be used to test new strategies to revert this phenotype.

A MDR phenotype is usually obtained by prolonged cell culture in the presence of low doses of chemotherapeutic drugs (for instance 0.01 and 2 *μ*g/ml 5‐FU) 6 or through rather complex procedures involving raising concentrations of drugs for long periods 16. In this , the HCT‐8‐resistant clone was selected by exposing the parental cells to a constant doxorubicin concentration for three repeated treatment cycles. This selection method mimics rather closely the clinical practice involving the appearance of chemoresistant clones after few cycles of treatment with single doses.

The resistant phenotype was induced by using doxorubicin as a typical P‐gp substrate and to exploit its autofluorescence to follow its intra‐ and extracellular movements.

First, we identified our cell line as HCT‐8 by DNA STR profiling with a percent match higher than 80%. The only mismatch was at the AMEL Y locus, a region reported sometimes as deleted 17 a CGH analysis performed on our cell line, pointed out a large deletion at the Y(p)11.2 region encompassing the AMEL locus.

To further characterize our HCT‐8/R clone, we evaluated its doubling time and cell cycle distribution; the resistant HCT‐8 subclone showed a doubling time longer than HCT‐8 parental cells and a rather variable percentage of S phase cells indicating an irregular duplication rate. Moreover, HCT‐8/R showed a marked increase in the percentage of cells in S phase after 48 h from seeding suggesting a duplication rate characterized by temporally circumscribed pulses.

Several biological phenomena connected with cancer such as metastasis could be evaluated in an ecological vision 18; the S phase distribution of HCT‐8/R cells could be compatible with a Darwinian selection of a clone equipped with survival strategies aimed at decreasing the probability of the coexistence of replicative events in the presence of cytotoxic drugs. These data are supported by those from trascriptomic analysis that shows how the HCT‐8/R presented a down‐regulations of genes encoding for proteins such as cyclin D1, involved in the cell cycle. Cyclin D1, in particular, controls the G1/S‐phase transition and its overexpression is associated with the increase in radiation‐induced apoptosis 19.

The HCT‐8/R cells are not only resistant to doxorubicin, but also to 5‐FU, whereas they are sensitive to oxaliplatin; the overexpression of efflux pumps such as P‐gp (up‐regulated in HCT‐8/R compared to the parental HCT‐8 cell line) is one of the most important mechanisms involved in the resistance to both 5‐FU and doxorubicin 20. On the contrary, resistance to oxaliplatin is mediated by other transporters such as ATP7A and ATP7B 21, genes that are not actually modified in HCT‐8/R.

The analysis carried out by transmission electron microscopy revealed a significant increase in cell volume in HCT‐8/R compared to the parental cells and the presence of cytokeratin fibers and vesicular bodies. These morphological phenomena could be correlated with an increased efflux of exogenous substances such as cytotoxic drugs. Morphologically, HCT‐8/R showed a higher percentage of polynucleolated cells in comparison to the HCT‐8 cells. The presence of more than a nucleolus per cell may be the indication of an adaptive process aimed to counteract nucleolus damages induced by doxorubicin. Ultrastructural studies showed the occurrence of doxorubicin nucleolar segregation in rat liver and heart cells 22, and Abe and coworkers found considerable alterations in the nucleolar morphology and function on Hela cells treated with doxorubicin. This treatment also induced a dramatic reduction of nucleolar 45S ribosomal precursor RNA biosynthesis 23. Moreover, recent findings show that changes in nucleolar morphology is related to cellular activity, such as growth, proliferation, and cell cycle progression 24. Cancer cells require continuous ribosome biogenesis and protein translation to maintain their high proliferation rate. In rapidly proliferating cancer cells, nucleolar proteins, which are involved in rRNA synthesis and processing, become more abundant, leading to nucleolar hypertrophy 25. Our resistant cells showed a longer doubling time than that of parental cells and a particular cell‐cycle phase distribution. The coexistence of polynucleolated cells and a lower proliferation rate in our model may be justified in the context of the complexity of MDR phenotype.

In fact, it is possible to hypothesize that on one hand, the lower proliferation rate observed in resistant cells might give an evolutionary advantage in the presence of an antineoplastic drug, but on the other hand, the same cells MDR cells might benefit from their supernumerary nucleoli to counteract chemotherapy‐induced nucleolar damages. In addition to cancer cells, a large nucleolus is a characteristic feature of stem cells and progenitor cells 24. The presence of polynucleolated cells in our model may be thus in concordance with their overexpression of stem cells markers such as CD44 and aldehyde dehydrogenase.

The MDR phenotype observed in the HCT‐8/R appears to be also related to their ability to evade the apoptotic process; the resistant clone in fact shows a lower percentage of apoptotic cells in comparison to parental cells after treatment with doxorubicin. In our data, doxorubicin doubled the percentage of apoptotic cells in parental clone, but did not change the basal level of apoptotic cells in HCT‐8/R. In the complexity of the MDR phenotype, these data are of particular interest since they indicate that resistant cells are able to escape the drug effect not only by extracellular efflux mediated by P‐gp overexpression but also by several other biological mechanisms such as a sort of apoptotic “immunity”. Indeed, we found several genes involved in the apoptotic process differentially expressed in HCT‐8/R cells compared to the HCT‐8 such as RAS related on chromosome 22 (RRP22) that promotes caspase‐independent cell death, damage‐regulated autophagy modulator (DRAM), and inositol polyphosphate‐5‐phosphatase (INPP5D), involved in the promotion of apoptosis through p53 pathway 26, 27, 28. To further support the involvement of apoptosis “immunity” in the complex phenomenon of MDR, our q‐PCR data confirmed that caspase 3, a key gene involved in the apoptotic pathway, was down‐regulated in HCT‐8/R compared to parental cells. Of note, the MDR phenotype was also be associated with the appearance of stem cell features such as a significant overexpression of the well‐known stem cells markers CD44 and aldehyde dehydrogenase 1 family member A2 (ALDH1A2). This aspect, together with the increased capacity of migration and invasion suggest that the HCT‐8/R clone may mimic a more aggressive form of colorectal cancer. Pasqualato and coworkers reported that the migration and invasion capacity was related to several morphological parameters such as area‐perimeter ratio, circularity, and deformability 29. HCT‐8/R showed a greater volume and a higher nucleus–cytoplasm ratio as well. The analysis of migration and invasion capacity was included in the characterization of our MDR cells because even if these traits are not directly involved in the onset of chemoresistance, they represent an additional aggressive feature which characterized our clone. Previous observations suggested that the acquisition of the multidrug resistance phenotype is associated with elevated invasion and metastasis that might be linked with P‐gp overexpression 30.

The most well‐known mechanism of drug resistance is the up‐regulation of ATP‐binding cassette (ABC) transporters, such as P‐gp 20. As mentioned before, the HCT‐8/R clone exhibited a 34‐fold overexpression of the gene encoding for the P‐gp pump. The increased expression of Pgp on cell membrane was confirmed by cytofluorimetric and immunocytochemical analysis. Immune‐electron microscopy, in particular, shed light into a peculiar distribution of P‐gp that is located in clusters along the cell membrane not involved in cell–cell junctions. The cytofluorimetric analysis and the quantification of doxorubicin autofluorescence, indicated an increased activity of Pgp in HCT‐8/R. In resistant cells, doxorubicin is located on cell membrane and it is almost absent inside the nucleus compared to parental cells. This peculiar distribution is compatible with the binding of doxorubicin with the increased number of efflux pumps such as P‐gp, in the membrane layer.

Another important mechanism involved in the onset of MDR phenotype is hypoxia. In fact, in response to tumor hypoxia, cells undergo adaptive modifications that promote their survival and intratumoral hypoxia is closely associated with increased resistance to conventional chemo‐ and radiotherapies 31. It is interesting to note that the most up‐regulated gene (+497 fold) in HCT‐8/R cells was the carbonyl reductase 1 (CBR1), a NADPH‐dependent enzyme that catalyzes a large number of endogenous and pharmacological substrates, reported to be up‐regulated in response to HIF‐1*α* and to be able to protect cancer cells against hypoxia and anticancer drugs such as cisplatin and doxorubicin, by reducing oxidative stress 32, 33. Moreover, in HCT‐8/R cells, a modest up‐regulation of three carbonic anhydrases (CA2, CA8, and CA13) involved in cellular hypoxia‐induced response were also observed.

In conclusion, because of its peculiar characteristics of cell cycle distribution, apoptosis, morphology, stem cells markers, migration, and invasion, our in vitro model is able to mimic an aggressive colorectal cancer with a MDR phenotype. These features make the HCT‐8/R clone particularly useful for the study of the mechanisms underlying the MDR and for testing new pharmacological strategies to overcome this phenomenon.

## Conflict of Interest

The authors declare no conflict of interest.

## Supporting information


**Figure S1.** Overview of the overall chromosomal aberrations found in the HCT‐8 cell line by aCGH analysis.

**Table S1.** List of genes found significantly modulated in HCT‐8 cell line compared to the HCT‐8/R‐resistant clone, with a fold change (FC) of at least ±2.

**Table S2.** List of pathways significantly enriched by GO‐Elite analysis.
