# Supplementary figures and images for: Development and characterization of an in vitro model of colorectal adenocarcinoma with MDR phenotype
Source: Cancer Med. 2016 Mar 25;5(6):1279–91. doi: 10.1002/cam4.694 (PMC4924386; doi:10.1002/cam4.694)

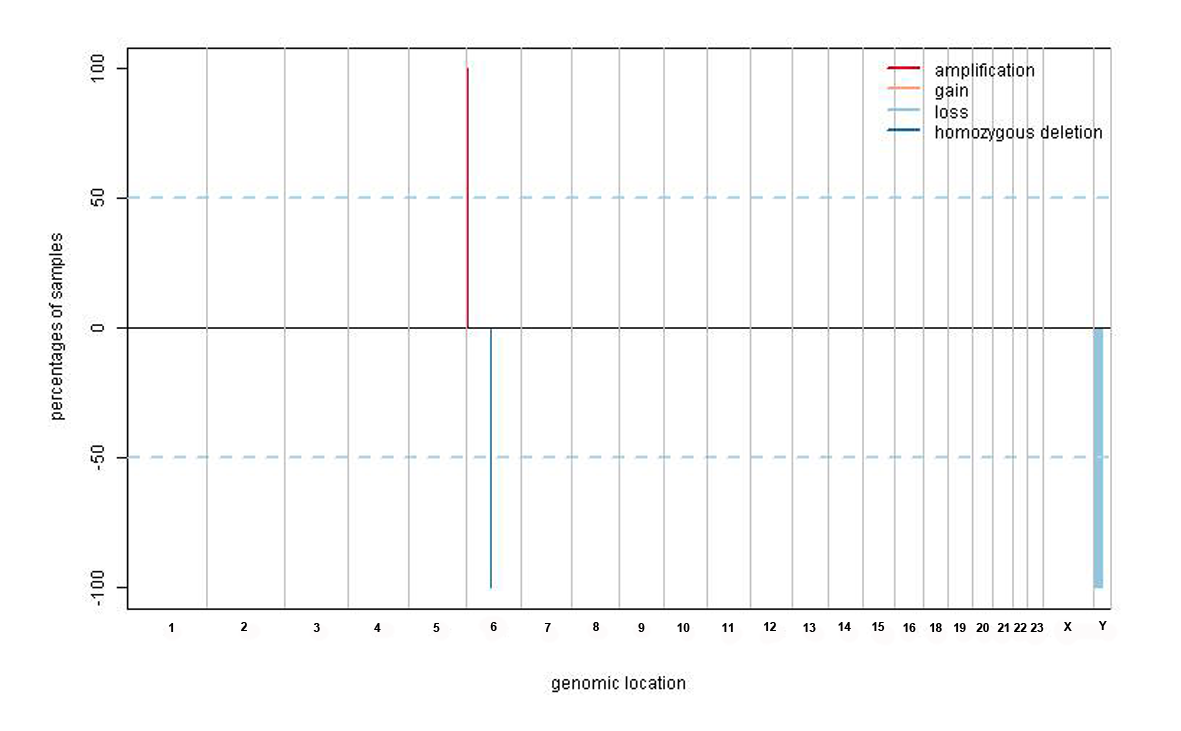

Supplement: Supplementary file 1 — Figure S1. Overview of the overall chromosomal aberrations found in the HCT‐8 cell line by aCGH analysis. [file CAM4-5-1279-s001.tif]
