# Supplementary material for: Development and characterization of an in vitro model of colorectal adenocarcinoma with MDR phenotype
Source: Cancer Med. 2016 Mar 25;5(6):1279–91. doi: 10.1002/cam4.694 (PMC4924386; doi:10.1002/cam4.694)
Supplement: Supplementary file 2 — Table S1. List of genes found significantly modulated in HCT‐8 cell line compared to the HCT‐8/R‐resistant clone, with a fold change (FC) of at least ±2. [file CAM4-5-1279-s002.doc]

**Table 1 supplementary .** List of genes found significantly modulated in HCT-8 cell line compared to the HCT-8/R resistant clone, with a fold change (FC) of at least ±2.

| **Gene Name** | **Description** | FC |
| --- | --- | --- |
| CBR1 | Homo sapiens carbonyl reductase 1 (CBR1), mRNA [NM_001757] | -497.27 |
| MAGEA9 | Homo sapiens melanoma antigen family A, 9 (MAGEA9), mRNA [NM_005365] | -402.62 |
| NAP1L5 | Homo sapiens nucleosome assembly protein 1-like 5 (NAP1L5), mRNA [NM_153757] | -310.06 |
| FSTL5 | Homo sapiens follistatin-like 5 (FSTL5), mRNA [NM_020116] | -252.65 |
| CLEC2B | Homo sapiens C-type lectin domain family 2, member B (CLEC2B), mRNA [NM_005127] | -177.50 |
| THC2747684 | Unknown | -151.45 |
| SCOC | Homo sapiens short coiled-coil protein (SCOC), mRNA [NM_032547] | -97.15 |
| EPHX2 | Homo sapiens epoxide hydrolase 2, cytoplasmic (EPHX2), mRNA [NM_001979] | -90.51 |
| HOXB6 | Homo sapiens homeobox B6 (HOXB6), mRNA [NM_018952] | -90.27 |
| TFPI | Homo sapiens tissue factor pathway inhibitor (lipoprotein-associated coagulation inhibitor) (TFPI), transcript variant 2, mRNA [NM_001032281] | -86.82 |
| MAGEA8 | Homo sapiens melanoma antigen family A, 8 (MAGEA8), mRNA [NM_005364] | -80.04 |
| BF213738 | BF213738 601847628F1 NIH_MGC_55 Homo sapiens cDNA clone IMAGE:4078519 5', mRNA sequence [BF213738] | -66.29 |
| CR594735 | full-length cDNA clone CS0DE011YB11 of Placenta of Homo sapiens (human). [CR594735] | -65.64 |
| RAB32 | Homo sapiens RAB32, member RAS oncogene family (RAB32), mRNA [NM_006834] | -60.26 |
| NLRP1 | Homo sapiens NLR family, pyrin domain containing 1, mRNA (cDNA clone MGC:57544 IMAGE:5756099), complete cds. [BC051787] | -57.99 |
| THC2573284 | Unknown | -51.31 |
| THC2534012 | Unknown | -51.04 |
| ALDH1A2 | Homo sapiens aldehyde dehydrogenase 1 family, member A2 (ALDH1A2), transcript variant 3, mRNA [NM_170697] | -50.33 |
| XAGE1 | Homo sapiens X antigen family, member 1 (XAGE1), transcript variant 2, mRNA [NM_133431] | -48.92 |
| SH2D1B | Homo sapiens SH2 domain containing 1B (SH2D1B), mRNA [NM_053282] | -46.66 |
| GAD1 | Homo sapiens glutamate decarboxylase 1 (brain, 67kDa) (GAD1), transcript variant GAD25, mRNA [NM_013445] | -40.36 |
| RECK | Homo sapiens cDNA clone MGC:71628 IMAGE:30336414, complete cds. [BC060806] | -39.97 |
| TMEM108 | Homo sapiens transmembrane protein 108 (TMEM108), mRNA [NM_023943] | -38.18 |
| ALDH1A2 | Homo sapiens aldehyde dehydrogenase 1 family, member A2 (ALDH1A2), transcript variant 3, mRNA [NM_170697] | -36.36 |
| MAGEC1 | Homo sapiens melanoma antigen family C, 1 (MAGEC1), mRNA [NM_005462] | -35.73 |
| C14orf72 | PREDICTED: Homo sapiens chromosome 14 open reading frame 72 (C14orf72), mRNA [XM_096733] | -34.36 |
| ABCB1 | Homo sapiens ATP-binding cassette, sub-family B (MDR/TAP), member 1 (ABCB1), mRNA [NM_000927] | -34.07 |
| AIG1 | Homo sapiens androgen-induced 1 (AIG1), mRNA [NM_016108] | -29.70 |
| TSPAN5 | Homo sapiens cDNA FLJ31097 fis, clone IMR321000210. [AK055659] | -27.99 |
| CDCP1 | Homo sapiens CUB domain containing protein 1 (CDCP1), transcript variant 1, mRNA [NM_022842] | -25.94 |
| A_24_P564761 | Unknown | -25.77 |
| CCRK | Homo sapiens cell cycle related kinase (CCRK), transcript variant 3, mRNA [NM_001039803] | -23.17 |
| MBOAT2 | Homo sapiens membrane bound O-acyltransferase domain containing 2 (MBOAT2), mRNA [NM_138799] | -22.71 |
| GAGE7 | Homo sapiens G antigen 7 (GAGE7), mRNA [NM_021123] | -22.68 |
| SKAP2 | Homo sapiens src kinase associated phosphoprotein 2 (SKAP2), mRNA [NM_003930] | -22.31 |
| TSPAN5 | Homo sapiens tetraspanin 5 (TSPAN5), mRNA [NM_005723] | -21.49 |
| ZDHHC23 | Homo sapiens zinc finger, DHHC-type containing 23 (ZDHHC23), mRNA [NM_173570] | -21.46 |
| RNF182 | Homo sapiens ring finger protein 182 (RNF182), mRNA [NM_152737] | -21.29 |
| MAGED1 | Homo sapiens NRAGE mRNA, complete cds. [AF217963] | -21.14 |
| TFPI | Homo sapiens tissue factor pathway inhibitor (lipoprotein-associated coagulation inhibitor) (TFPI), transcript variant 1, mRNA [NM_006287] | -20.40 |
| HBE1 | Homo sapiens hemoglobin, epsilon 1 (HBE1), mRNA [NM_005330] | -19.97 |
| BE672039 | 7a50b09.x1 NCI_CGAP_GC6 Homo sapiens cDNA clone IMAGE:3222137 3', mRNA sequence [BE672039] | -19.07 |
| THC2488083 | AF044209 nuclear receptor co-repressor N-CoR {Homo sapiens} (exp=-1; wgp=0; cg=0), partial (8%) [THC2488083] | -19.00 |
| DPYS | Homo sapiens dihydropyrimidinase (DPYS), mRNA [NM_001385] | -18.79 |
| ENST00000367932 | Homo sapiens cDNA FLJ43132 fis, clone CTONG3005813. [AK125122] | -18.68 |
| MAGEC2 | Homo sapiens melanoma antigen family C, 2 (MAGEC2), mRNA [NM_016249] | -18.53 |
| PPP1R14C | Homo sapiens protein phosphatase 1, regulatory (inhibitor) subunit 14C (PPP1R14C), mRNA [NM_030949] | -18.02 |
| LANCL3 | Homo sapiens LanC lantibiotic synthetase component C-like 3 (bacterial) (LANCL3), mRNA [NM_198511] | -17.11 |
| CD36 | Homo sapiens CD36 molecule (thrombospondin receptor) (CD36), transcript variant 2, mRNA [NM_001001547] | -17.07 |
| CD36 | CD36=collagen type I/thrombospondin receptor {one exon} [human, mRNA Partial, 369 nt]. [S67044] | -16.90 |
| LOC254848 | Homo sapiens hypothetical protein LOC254848, mRNA (cDNA clone IMAGE:4544270), with apparent retained intron. [BC014113] | -16.86 |
| HBE1 | Homo sapiens hemoglobin, epsilon 1 (HBE1), mRNA [NM_005330] | -16.69 |
| HBBP1 | Homo sapiens hemoglobin, beta pseudogene 1 (HBBP1) on chromosome 11 [NR_001589] | -16.43 |
| FOXC1 | Homo sapiens forkhead box C1 (FOXC1), mRNA [NM_001453] | -16.02 |
| CRYM | Homo sapiens crystallin, mu (CRYM), transcript variant 1, mRNA [NM_001888] | -15.67 |
| PRR16 | Homo sapiens proline rich 16 (PRR16), mRNA [NM_016644] | -14.63 |
| CYP26A1 | Homo sapiens cytochrome P450, family 26, subfamily A, polypeptide 1 (CYP26A1), transcript variant 2, mRNA [NM_057157] | -14.18 |
| BC031359 | Homo sapiens, clone IMAGE:4778855, mRNA, partial cds. [BC031359] | -13.90 |
| SQRDL | Homo sapiens sulfide quinone reductase-like (yeast) (SQRDL), mRNA [NM_021199] | -13.17 |
| OR51B5 | Homo sapiens olfactory receptor, family 51, subfamily B, member 5 (OR51B5), mRNA [NM_001005567] | -13.15 |
| A_32_P75141 | Unknown | -13.04 |
| A_32_P113462 | Unknown | -12.82 |
| BC038245 | Homo sapiens, clone IMAGE:5241654, mRNA. [BC038245] | -12.71 |
| ELOVL7 | Homo sapiens ELOVL family member 7, elongation of long chain fatty acids (yeast) (ELOVL7), mRNA [NM_024930] | -12.60 |
| VPS13D | Homo sapiens vacuolar protein sorting 13 homolog D (S. cerevisiae) (VPS13D), transcript variant 1, mRNA [NM_015378] | -11.25 |
| SMAD1 | Homo sapiens SMAD family member 1 (SMAD1), transcript variant 1, mRNA [NM_005900] | -11.02 |
| BEX2 | Homo sapiens brain expressed X-linked 2 (BEX2), mRNA [NM_032621] | -10.37 |
| AK096020 | Homo sapiens cDNA FLJ38701 fis, clone KIDNE2002198. [AK096020] | -9.99 |
| A_32_P21742 | Unknown | -9.63 |
| ANK3 | Homo sapiens ankyrin 3, node of Ranvier (ankyrin G) (ANK3), transcript variant 1, mRNA [NM_020987] | -9.26 |
| SPAG16 | Homo sapiens sperm associated antigen 16 (SPAG16), transcript variant 2, mRNA [NM_001025436] | -9.13 |
| CA2 | Homo sapiens carbonic anhydrase II (CA2), mRNA [NM_000067] | -9.02 |
| ANK3 | Homo sapiens ankyrin 3, node of Ranvier (ankyrin G) (ANK3), transcript variant 1, mRNA [NM_020987] | -8.81 |
| FES | Homo sapiens feline sarcoma oncogene (FES), mRNA [NM_002005] | -8.72 |
| PELI2 | Homo sapiens pellino homolog 2 (Drosophila) (PELI2), mRNA [NM_021255] | -8.70 |
| HBB | Homo sapiens hemoglobin, beta (HBB), mRNA [NM_000518] | -8.50 |
| LIMS3 | Homo sapiens LIM and senescent cell antigen-like domains 3 (LIMS3), mRNA [NM_033514] | -8.49 |
| PPP2R5C | Homo sapiens mRNA; cDNA DKFZp761H0317 (from clone DKFZp761H0317). [AL834350] | -8.28 |
| SCG3 | Homo sapiens secretogranin III (SCG3), mRNA [NM_013243] | -8.22 |
| BC035377 | Homo sapiens cDNA clone IMAGE:4826240. [BC035377] | -8.19 |
| LONRF3 | Homo sapiens LON peptidase N-terminal domain and ring finger 3 (LONRF3), transcript variant 1, mRNA [NM_001031855] | -8.09 |
| RHD | Homo sapiens Rh blood group, D antigen (RHD), mRNA [NM_016124] | -7.92 |
| ISG20 | Homo sapiens interferon stimulated exonuclease gene 20kDa (ISG20), mRNA [NM_002201] | -7.92 |
| LOC123688 | Homo sapiens similar to RIKEN cDNA C630028N24 gene (LOC123688), mRNA [NM_001013619] | -7.70 |
| ENST00000334770 | Homo sapiens cDNA FLJ16078 fis, clone NT2NE2003252, weakly similar to Human putative serine/threonine protein kinase PRK (prk) mRNA. [AK122648] | -7.69 |
| FCGRT | Homo sapiens Fc fragment of IgG, receptor, transporter, alpha (FCGRT), mRNA [NM_004107] | -7.59 |
| ASPH | Homo sapiens aspartate beta-hydroxylase (ASPH), transcript variant 4, mRNA [NM_032467] | -7.57 |
| AK126242 | Homo sapiens cDNA FLJ44254 fis, clone TKIDN2009641. [AK126242] | -7.53 |
| A_32_P219377 | Unknown | -7.46 |
| IMMP2L | Homo sapiens IMP2 inner mitochondrial membrane peptidase-like (S. cerevisiae) (IMMP2L), mRNA [NM_032549] | -7.42 |
| MBOAT2 | O-acyltransferase (membrane bound) domain containing 2 [Source:RefSeq_peptide;Acc:NP_620154] [ENST00000354442] | -7.23 |
| SH3GL3 | Homo sapiens SH3-domain GRB2-like 3 (SH3GL3), mRNA [NM_003027] | -7.19 |
| EPB42 | Homo sapiens erythrocyte membrane protein band 4.2 (EPB42), mRNA [NM_000119] | -7.16 |
| HRASLS3 | Homo sapiens HRAS-like suppressor 3 (HRASLS3), mRNA [NM_007069] | -6.79 |
| HEY1 | Homo sapiens hairy/enhancer-of-split related with YRPW motif 1 (HEY1), transcript variant 2, mRNA [NM_001040708] | -6.76 |
| ASPH | Homo sapiens aspartate beta-hydroxylase (ASPH), transcript variant 3, mRNA [NM_032466] | -6.75 |
| TUBA1 | Homo sapiens tubulin, alpha 1 (TUBA1), mRNA [NM_006000] | -6.73 |
| RP5-875H10.1 | Homo sapiens SAM domain containing 1 (LOC389432), mRNA [NM_001030060] | -6.66 |
| IDS | Homo sapiens iduronate 2-sulfatase (Hunter syndrome) (IDS), transcript variant 1, mRNA [NM_000202] | -6.59 |
| PPIC | Homo sapiens peptidylprolyl isomerase C (cyclophilin C) (PPIC), mRNA [NM_000943] | -6.36 |
| ZDHHC11 | Homo sapiens zinc finger, DHHC-type containing 11 (ZDHHC11), mRNA [NM_024786] | -6.30 |
| MLC1 | Homo sapiens megalencephalic leukoencephalopathy with subcortical cysts 1 (MLC1), transcript variant 1, mRNA [NM_015166] | -6.28 |
| HSPC159 | Homo sapiens galectin-related protein (HSPC159), mRNA [NM_014181] | -6.22 |
| FBXO16 | Homo sapiens F-box protein 16 (FBXO16), mRNA [NM_172366] | -6.18 |
| PDGFA | Homo sapiens platelet-derived growth factor alpha polypeptide (PDGFA), transcript variant 1, mRNA [NM_002607] | -6.11 |
| HIST2H2AA4 | Homo sapiens histone cluster 2, H2aa4 (HIST2H2AA4), mRNA [NM_001040874] | -6.09 |
| AATK | Homo sapiens cDNA FLJ16758 fis, clone BRACE3038687, moderately similar to Homo sapiens apoptosis-associated tyrosine kinase (AATK). [AK131529] | -5.97 |
| ZNF697 | Homo sapiens cDNA: FLJ23366 fis, clone HEP15665. [AK027019] | -5.92 |
| TMC5 | Homo sapiens transmembrane channel-like 5 (TMC5), mRNA [NM_024780] | -5.91 |
| ZDHHC11 | Homo sapiens zinc finger, DHHC-type containing 11 (ZDHHC11), mRNA [NM_024786] | -5.83 |
| AKR1CL2 | Homo sapiens mRNA for aldo-keto reductase related protein 1, complete cds. [AB040820] | -5.82 |
| THC2715094 | Q4J972_SULAC (Q4J972) Conserved Crenarchaeal protein, partial (9%) [THC2715094] | -5.82 |
| PPIC | Homo sapiens peptidylprolyl isomerase C (cyclophilin C) (PPIC), mRNA [NM_000943] | -5.81 |
| WDR54 | Homo sapiens WD repeat domain 54 (WDR54), mRNA [NM_032118] | -5.81 |
| EAF2 | Homo sapiens ELL associated factor 2 (EAF2), mRNA [NM_018456] | -5.76 |
| REEP2 | Homo sapiens receptor accessory protein 2 (REEP2), mRNA [NM_016606] | -5.75 |
| ACYP2 | AGENCOURT_6818400 NIH_MGC_114 Homo sapiens cDNA clone IMAGE:5762573 5', mRNA sequence [BQ066852] | -5.66 |
| CD44 | Homo sapiens CD44 molecule (Indian blood group) (CD44), transcript variant 1, mRNA [NM_000610] | -5.63 |
| DHRS3 | Homo sapiens dehydrogenase/reductase (SDR family) member 3 (DHRS3), mRNA [NM_004753] | -5.61 |
| C9orf58 | Homo sapiens chromosome 9 open reading frame 58 (C9orf58), transcript variant 2, mRNA [NM_001002260] | -5.53 |
| TUBA1 | Homo sapiens tubulin, alpha 1 (TUBA1), mRNA [NM_006000] | -5.50 |
| BC052334 | Homo sapiens cDNA clone IMAGE:6176570, partial cds. [BC052334] | -5.50 |
| CALB1 | Homo sapiens calbindin 1, 28kDa (CALB1), mRNA [NM_004929] | -5.42 |
| AK023629 | Homo sapiens cDNA FLJ13567 fis, clone PLACE1008331. [AK023629] | -5.39 |
| FBXL16 | Homo sapiens F-box and leucine-rich repeat protein 16 (FBXL16), mRNA [NM_153350] | -5.38 |
| DYNLT3 | Dynein light chain Tctex-type 3 (T-complex-associated testis-expressed 1-like) (Protein 91/23). [Source:Uniprot/SWISSPROT;Acc:P51808] [ENST00000378578] | -5.37 |
| BX419129 | BX419129 Homo sapiens FETAL BRAIN Homo sapiens cDNA clone CS0DF013YC22 5-PRIME, mRNA sequence [BX419129] | -5.37 |
| CD9 | Homo sapiens CD9 molecule (CD9), mRNA [NM_001769] | -5.34 |
| A_32_P99804 | Unknown | -5.33 |
| PDCD1 | Homo sapiens programmed cell death 1 (PDCD1), mRNA [NM_005018] | -5.26 |
| DHRS2 | Homo sapiens dehydrogenase/reductase (SDR family) member 2 (DHRS2), transcript variant 1, mRNA [NM_182908] | -5.25 |
| CUGBP1 | Homo sapiens CUG triplet repeat, RNA binding protein 1 (CUGBP1), transcript variant 2, mRNA [NM_198700] | -5.24 |
| BC040051 | Homo sapiens, clone IMAGE:6018828, mRNA. [BC040051] | -5.23 |
| PSPH | Homo sapiens phosphoserine phosphatase (PSPH), mRNA [NM_004577] | -5.22 |
| THC2707492 | Unknown | -5.18 |
| THC2605277 | Q8NG87_HUMAN (Q8NG87) Seven transmembrane helix receptor (Fragment), complete [THC2605277] | -5.06 |
| BG031574 | 602299712F1 NIH_MGC_87 Homo sapiens cDNA clone IMAGE:4394138 5', mRNA sequence [BG031574] | -5.04 |
| RABGAP1L | Homo sapiens RAB GTPase activating protein 1-like (RABGAP1L), transcript variant 1, mRNA [NM_014857] | -4.96 |
| DYNLT3 | Homo sapiens dynein, light chain, Tctex-type 3 (DYNLT3), mRNA [NM_006520] | -4.94 |
| EBI2 | Homo sapiens Epstein-Barr virus induced gene 2 (lymphocyte-specific G protein-coupled receptor) (EBI2), mRNA [NM_004951] | -4.91 |
| ZNF506 | Homo sapiens mRNA; cDNA DKFZp761G18121 (from clone DKFZp761G18121). [AL136548] | -4.88 |
| RHCE | Homo sapiens Rh blood group, CcEe antigens (RHCE), transcript variant 1, mRNA [NM_020485] | -4.85 |
| CR624390 | full-length cDNA clone CS0DC018YB19 of Neuroblastoma Cot 25-normalized of Homo sapiens (human). [CR624390] | -4.85 |
| HIST1H1C | Homo sapiens histone cluster 1, H1c (HIST1H1C), mRNA [NM_005319] | -4.85 |
| RRAGD | Homo sapiens Ras-related GTP binding D (RRAGD), mRNA [NM_021244] | -4.80 |
| NNMT | Homo sapiens nicotinamide N-methyltransferase (NNMT), mRNA [NM_006169] | -4.79 |
| BF184174 | 601843220F1 NIH_MGC_54 Homo sapiens cDNA clone IMAGE:4063925 5', mRNA sequence [BF184174] | -4.77 |
| FAM20B | Homo sapiens family with sequence similarity 20, member B (FAM20B), mRNA [NM_014864] | -4.75 |
| BF378976 | BF378976 QV1-UM0099-020400-141-b12_1 UM0099 Homo sapiens cDNA, mRNA sequence [BF378976] | -4.73 |
| SCG3 | Homo sapiens secretogranin III (SCG3), mRNA [NM_013243] | -4.73 |
| C21orf119 | Homo sapiens chromosome 21 open reading frame 119 (C21orf119), mRNA [NM_032910] | -4.62 |
| TMEM16E | Transmembrane protein 16E (Gnathodiaphyseal dysplasia 1 protein). [Source:Uniprot/SWISSPROT;Acc:Q75V66] [ENST00000324559] | -4.62 |
| C8orf70 | Homo sapiens chromosome 8 open reading frame 70 (C8orf70), mRNA [NM_016010] | -4.61 |
| HIST2H2AA4 | Homo sapiens histone cluster 2, H2aa4 (HIST2H2AA4), mRNA [NM_001040874] | -4.59 |
| FLJ35767 | Homo sapiens FLJ35767 protein (FLJ35767), mRNA [NM_207459] | -4.58 |
| CYB5A | Homo sapiens cytochrome b5 type A (microsomal) (CYB5A), transcript variant 2, mRNA [NM_001914] | -4.58 |
| NAG | Homo sapiens neuroblastoma-amplified protein (NAG), mRNA [NM_015909] | -4.56 |
| GSTZ1 | Homo sapiens glutathione transferase zeta 1 (maleylacetoacetate isomerase) (GSTZ1), transcript variant 1, mRNA [NM_145870] | -4.56 |
| FLJ36000 | Homo sapiens cDNA FLJ36000 fis, clone TESTI2015180. [AK093319] | -4.54 |
| IDS | Homo sapiens iduronate 2-sulfatase (Hunter syndrome) (IDS), transcript variant 2, mRNA [NM_006123] | -4.54 |
| HSPC159 | galectin-related protein (HSPC159), mRNA [Source:RefSeq_dna;Acc:NM_014181] [ENST00000238875] | -4.53 |
| GABARAPL1 | Homo sapiens GABA(A) receptor-associated protein like 1 (GABARAPL1), mRNA [NM_031412] | -4.49 |
| EPPK1 | Homo sapiens epiplakin 1 (EPPK1), mRNA [NM_031308] | -4.47 |
| C1orf183 | Homo sapiens chromosome 1 open reading frame 183 (C1orf183), transcript variant 1, mRNA [NM_019099] | -4.46 |
| MAGEB2 | Homo sapiens melanoma antigen family B, 2 (MAGEB2), mRNA [NM_002364] | -4.43 |
| MGC23284 | Homo sapiens FP17581 mRNA, complete cds. [AY203928] | -4.42 |
| A_32_P209163 | Unknown | -4.41 |
| TPD52 | Homo sapiens tumor protein D52 (TPD52), transcript variant 1, mRNA [NM_001025252] | -4.40 |
| THC2647658 | Unknown | -4.37 |
| THC2695815 | Unknown | -4.37 |
| AGGF1 | Homo sapiens angiogenic factor with G patch and FHA domains 1, mRNA (cDNA clone IMAGE:3659316), complete cds. [BC002828] | -4.36 |
| A_24_P112542 | Unknown | -4.34 |
| ASRGL1 | Homo sapiens asparaginase like 1 (ASRGL1), mRNA [NM_025080] | -4.32 |
| AA484677 | AA484677 ne64a07.s1 NCI_CGAP_Alv1 Homo sapiens cDNA clone IMAGE:909012, mRNA sequence [AA484677] | -4.32 |
| NCOR1 | Homo sapiens nuclear receptor co-repressor 1 (NCOR1), mRNA [NM_006311] | -4.31 |
| C9orf58 | Homo sapiens chromosome 9 open reading frame 58 (C9orf58), transcript variant 2, mRNA [NM_001002260] | -4.30 |
| AA418814 | AA418814 zw01a02.s1 Soares_NhHMPu_S1 Homo sapiens cDNA clone IMAGE:767978 3', mRNA sequence [AA418814] | -4.29 |
| THC2536579 | Unknown | -4.29 |
| C9orf9 | Homo sapiens chromosome 9 open reading frame 9 (C9orf9), mRNA [NM_018956] | -4.28 |
| OSCAR | Homo sapiens osteoclast-associated receptor (OSCAR), transcript variant 1, mRNA [NM_206818] | -4.26 |
| NMU | Homo sapiens neuromedin U (NMU), mRNA [NM_006681] | -4.25 |
| APBB1 | Homo sapiens amyloid beta (A4) precursor protein-binding, family B, member 1 (Fe65) (APBB1), transcript variant 1, mRNA [NM_001164] | -4.23 |
| HTATIP2 | Homo sapiens HIV-1 Tat interactive protein 2, 30kDa (HTATIP2), mRNA [NM_006410] | -4.22 |
| SEC22B | Homo sapiens SEC22 vesicle trafficking protein homolog B (S. cerevisiae) (SEC22B), mRNA [NM_004892] | -4.20 |
| HBD | Homo sapiens hemoglobin, delta (HBD), mRNA [NM_000519] | -4.19 |
| THC2501618 | O51948_NEIGO (O51948) Outer membrane protein I precursor, partial (6%) [THC2534745] | -4.16 |
| OSBPL6 | Homo sapiens oxysterol binding protein-like 6 (OSBPL6), transcript variant 1, mRNA [NM_032523] | -4.16 |
| HTATIP2 | Homo sapiens alternatively spliced product of metastasis-suppressor gene CC3 (TC3) mRNA, complete cds. [AF092095] | -4.15 |
| CCDC88 | Homo sapiens coiled-coil domain containing 88 (CCDC88), mRNA [NM_032251] | -4.15 |
| TUBA1 | Homo sapiens tubulin, alpha 1 (TUBA1), mRNA [NM_006000] | -4.14 |
| GDF15 | Homo sapiens growth differentiation factor 15 (GDF15), mRNA [NM_004864] | -4.12 |
| DNAL1 | Homo sapiens dynein, axonemal, light chain 1 (DNAL1), mRNA [NM_031427] | -4.10 |
| OVOS2 | Homo sapiens ovostatin 2, mRNA (cDNA clone IMAGE:4827636). [BC039117] | -4.07 |
| HBD | Homo sapiens hemoglobin, delta (HBD), mRNA [NM_000519] | -4.07 |
| FBXO3 | Homo sapiens F-box protein 3 (FBXO3), transcript variant 2, mRNA [NM_033406] | -4.06 |
| SNX10 | Homo sapiens sorting nexin 10 (SNX10), mRNA [NM_013322] | -4.04 |
| EEF1A2 | Homo sapiens eukaryotic translation elongation factor 1 alpha 2 (EEF1A2), mRNA [NM_001958] | -4.04 |
| HS1BP3 | Homo sapiens HCLS1 binding protein 3 (HS1BP3), mRNA [NM_022460] | -3.99 |
| A_32_P126647 | Unknown | -3.96 |
| BCAN | Homo sapiens brevican, mRNA (cDNA clone IMAGE:3618761), partial cds. [BC005081] | -3.96 |
| NOTCH2 | Homo sapiens Notch homolog 2 (Drosophila) (NOTCH2), mRNA [NM_024408] | -3.95 |
| CAV2 | Homo sapiens caveolin 2 (CAV2), transcript variant 1, mRNA [NM_001233] | -3.94 |
| DHRS2 | Homo sapiens dehydrogenase/reductase (SDR family) member 2 (DHRS2), transcript variant 1, mRNA [NM_182908] | -3.94 |
| ASRGL1 | Homo sapiens asparaginase like 1 (ASRGL1), mRNA [NM_025080] | -3.94 |
| SLC22A18 | Homo sapiens solute carrier family 22 (organic cation transporter), member 18 (SLC22A18), transcript variant 2, mRNA [NM_183233] | -3.94 |
| ABLIM1 | Homo sapiens actin binding LIM protein 1 (ABLIM1), transcript variant 3, mRNA [NM_001003408] | -3.93 |
| LOC154822 | Homo sapiens hypothetical protein LOC154822, mRNA (cDNA clone IMAGE:3528300), partial cds. [BC013024] | -3.92 |
| C15orf26 | Homo sapiens chromosome 15 open reading frame 26 (C15orf26), mRNA [NM_173528] | -3.91 |
| MLF1 | Homo sapiens myeloid leukemia factor 1 (MLF1), mRNA [NM_022443] | -3.90 |
| C9orf19 | Homo sapiens chromosome 9 open reading frame 19 (C9orf19), mRNA [NM_022343] | -3.90 |
| A_32_P112263 | Unknown | -3.89 |
| HIST1H2AD | Homo sapiens histone cluster 1, H2ad (HIST1H2AD), mRNA [NM_021065] | -3.87 |
| A_32_P46351 | Unknown | -3.86 |
| ADSSL1 | Homo sapiens adenylosuccinate synthase like 1 (ADSSL1), transcript variant 1, mRNA [NM_199165] | -3.84 |
| LOC283663 | Homo sapiens cDNA FLJ33196 fis, clone ADRGL2006034. [AK090515] | -3.83 |
| AK123679 | Homo sapiens cDNA FLJ41685 fis, clone HCASM2006338. [AK123679] | -3.81 |
| LEPROT | Homo sapiens leptin receptor overlapping transcript (LEPROT), mRNA [NM_017526] | -3.81 |
| LOC654342 | PREDICTED: Homo sapiens lymphocyte-specific protein 1 (LSP1), mRNA [XM_946374] | -3.78 |
| LRFN3 | Homo sapiens leucine rich repeat and fibronectin type III domain containing 3 (LRFN3), mRNA [NM_024509] | -3.77 |
| HIST1H2AE | Homo sapiens histone cluster 1, H2ae (HIST1H2AE), mRNA [NM_021052] | -3.76 |
| PNPLA4 | Homo sapiens patatin-like phospholipase domain containing 4 (PNPLA4), mRNA [NM_004650] | -3.76 |
| KIAA1609 | Homo sapiens KIAA1609 (KIAA1609), mRNA [NM_020947] | -3.75 |
| CASP4 | Homo sapiens caspase 4, apoptosis-related cysteine peptidase (CASP4), transcript variant gamma, mRNA [NM_033306] | -3.74 |
| HBG1 | Homo sapiens hemoglobin, gamma A (HBG1), mRNA [NM_000559] | -3.73 |
| FER | Proto-oncogene tyrosine-protein kinase FER (EC 2.7.10.2) (p94-FER) (c- FER). [Source:Uniprot/SWISSPROT;Acc:P16591] [ENST00000379734] | -3.73 |
| ASRGL1 | Homo sapiens asparaginase like 1, mRNA (cDNA clone IMAGE:3952485), complete cds. [BC006267] | -3.72 |
| CTSF | Homo sapiens cathepsin F (CTSF), mRNA [NM_003793] | -3.71 |
| THC2748857 | Q2RX18_RHORT (Q2RX18) Two component CheB methylesterase , partial (5%) [THC2748857] | -3.69 |
| RABGAP1L | Homo sapiens RAB GTPase activating protein 1-like (RABGAP1L), transcript variant 1, mRNA [NM_014857] | -3.69 |
| CASP4 | Homo sapiens caspase 4, apoptosis-related cysteine peptidase (CASP4), transcript variant gamma, mRNA [NM_033306] | -3.67 |
| THC2677011 | Unknown | -3.67 |
| THC2499508 | Unknown | -3.67 |
| THC2609515 | MMU67770 ribosomal protein S26 {Mus musculus} (exp=-1; wgp=0; cg=0), partial (16%) [THC2609515] | -3.65 |
| TMEM22 | Homo sapiens transmembrane protein 22 (TMEM22), mRNA [NM_025246] | -3.64 |
| DDB2 | Homo sapiens damage-specific DNA binding protein 2, 48kDa (DDB2), mRNA [NM_000107] | -3.63 |
| THC2663297 | Q5VWT3_HUMAN (Q5VWT3) Complement component (3b/4b) receptor 1-like, partial (27%) [THC2663297] | -3.62 |
| THC2611974 | AY250221 nogo receptor-like 3 {Homo sapiens} (exp=-1; wgp=0; cg=0), partial (12%) [THC2611974] | -3.60 |
| NPDC1 | Homo sapiens neural proliferation, differentiation and control, 1 (NPDC1), mRNA [NM_015392] | -3.59 |
| BBS12 | Homo sapiens Bardet-Biedl syndrome 12 (BBS12), mRNA [NM_152618] | -3.57 |
| PQLC3 | Homo sapiens PQ loop repeat containing 3 (PQLC3), mRNA [NM_152391] | -3.57 |
| GPD2 | Homo sapiens glycerol-3-phosphate dehydrogenase 2 (mitochondrial) (GPD2), mRNA [NM_000408] | -3.56 |
| NPC2 | Homo sapiens Niemann-Pick disease, type C2 (NPC2), mRNA [NM_006432] | -3.56 |
| A_32_P109165 | Unknown | -3.55 |
| KIAA0265 | Homo sapiens KIAA0265 protein (KIAA0265), mRNA [NM_014997] | -3.55 |
| MLF1 | Homo sapiens myeloid leukemia factor 1 (MLF1), mRNA [NM_022443] | -3.55 |
| BC011940 | Homo sapiens cDNA clone IMAGE:4329532, partial cds. [BC011940] | -3.54 |
| BC020539 | Homo sapiens, clone IMAGE:3048919, mRNA, partial cds. [BC020539] | -3.54 |
| C9orf95 | Homo sapiens chromosome 9 open reading frame 95 (C9orf95), mRNA [NM_017881] | -3.53 |
| NAG | Homo sapiens neuroblastoma-amplified protein (NAG), mRNA [NM_015909] | -3.53 |
| ARMC9 | Homo sapiens armadillo repeat containing 9 (ARMC9), mRNA [NM_025139] | -3.53 |
| OGDHL | Homo sapiens oxoglutarate dehydrogenase-like (OGDHL), mRNA [NM_018245] | -3.53 |
| GABARAPL1 | Homo sapiens GABA(A) receptor-associated protein like 1 (GABARAPL1), mRNA [NM_031412] | -3.52 |
| HBA2 | Homo sapiens hemoglobin, alpha 2 (HBA2), mRNA [NM_000517] | -3.51 |
| TDH | Homo sapiens cDNA FLJ25033 fis, clone CBL02720. [AK057762] | -3.51 |
| HIST1H2AM | Homo sapiens histone cluster 1, H2am (HIST1H2AM), mRNA [NM_003514] | -3.51 |
| TTC26 | Homo sapiens tetratricopeptide repeat domain 26 (TTC26), mRNA [NM_024926] | -3.48 |
| PCFT | Homo sapiens proton-coupled folate transporter (PCFT), mRNA [NM_080669] | -3.47 |
| HEBP1 | Homo sapiens heme binding protein 1 (HEBP1), mRNA [NM_015987] | -3.47 |
| THC2507805 | Unknown | -3.45 |
| ZC3H12D | CDNA FLJ46041 fis, clone SPLEN2036608. [Source:Uniprot/SPTREMBL;Acc:Q6ZRW2] [ENST00000389942] | -3.45 |
| C14orf179 | Homo sapiens chromosome 14 open reading frame 179 (C14orf179), mRNA [NM_052873] | -3.44 |
| RABAC1 | Homo sapiens Rab acceptor 1 (prenylated) (RABAC1), mRNA [NM_006423] | -3.42 |
| MAP3K10 | Homo sapiens mitogen-activated protein kinase kinase kinase 10 (MAP3K10), mRNA [NM_002446] | -3.42 |
| APH1B | Homo sapiens anterior pharynx defective 1 homolog B (C. elegans) (APH1B), mRNA [NM_031301] | -3.42 |
| HIST1H1E | Homo sapiens histone cluster 1, H1e (HIST1H1E), mRNA [NM_005321] | -3.41 |
| AK098629 | Homo sapiens cDNA FLJ25763 fis, clone TST06294. [AK098629] | -3.41 |
| ICAM2 | Homo sapiens intercellular adhesion molecule 2 (ICAM2), mRNA [NM_000873] | -3.40 |
| C12orf5 | Homo sapiens chromosome 12 open reading frame 5 (C12orf5), mRNA [NM_020375] | -3.40 |
| IL10RA | Homo sapiens interleukin 10 receptor, alpha (IL10RA), mRNA [NM_001558] | -3.39 |
| SPAG9 | Homo sapiens sperm associated antigen 9, mRNA (cDNA clone IMAGE:3351321), complete cds. [BC007524] | -3.39 |
| BC018626 | Homo sapiens, Similar to hect domain and RLD 2, clone IMAGE:4581928, mRNA. [BC018626] | -3.35 |
| LOC158830 | Homo sapiens similar to Ab2-183 (LOC158830), mRNA [NM_001025265] | -3.35 |
| GPD1 | Homo sapiens glycerol-3-phosphate dehydrogenase 1 (soluble) (GPD1), mRNA [NM_005276] | -3.34 |
| TMEM22 | Homo sapiens transmembrane protein 22 (TMEM22), mRNA [NM_025246] | -3.33 |
| EMX1 | Homo sapiens empty spiracles homolog 1 (Drosophila) (EMX1), transcript variant 2, mRNA [NM_001040404] | -3.32 |
| BU602485 | AGENCOURT_10015118 NIH_MGC_142 Homo sapiens cDNA clone IMAGE:6496567 5', mRNA sequence [BU602485] | -3.32 |
| TPD52 | Homo sapiens tumor protein D52 (TPD52), transcript variant 1, mRNA [NM_001025252] | -3.31 |
| KCTD9 | Homo sapiens clone FLB9630 PRO2603 mRNA, complete cds. [AF130091] | -3.31 |
| RSHL2 | Homo sapiens radial spokehead-like 2 (RSHL2), mRNA [NM_031924] | -3.31 |
| HIST2H2AC | Homo sapiens histone cluster 2, H2ac (HIST2H2AC), mRNA [NM_003517] | -3.31 |
| BPGM | Homo sapiens 2,3-bisphosphoglycerate mutase (BPGM), transcript variant 2, mRNA [NM_199186] | -3.29 |
| RNF141 | Homo sapiens ring finger protein 141 (RNF141), mRNA [NM_016422] | -3.29 |
| BM932296 | UI-E-EJ1-ajk-k-19-0-UI.r1 UI-E-EJ1 Homo sapiens cDNA clone UI-E-EJ1-ajk-k-19-0-UI 5', mRNA sequence [BM932296] | -3.29 |
| MAP7 | Homo sapiens microtubule-associated protein 7 (MAP7), mRNA [NM_003980] | -3.28 |
| CREB3L2 | Homo sapiens cAMP responsive element binding protein 3-like 2, mRNA (cDNA clone IMAGE:4185677), complete cds. [BC063666] | -3.28 |
| CYP2R1 | Homo sapiens cytochrome P450, family 2, subfamily R, polypeptide 1 (CYP2R1), mRNA [NM_024514] | -3.27 |
| HBG1 | Homo sapiens hemoglobin, gamma A (HBG1), mRNA [NM_000559] | -3.27 |
| BC038432 | Homo sapiens cDNA clone IMAGE:5162874, partial cds. [BC038432] | -3.26 |
| ITPR1 | Homo sapiens inositol 1,4,5-triphosphate receptor, type 1 (ITPR1), mRNA [NM_002222] | -3.26 |
| THC2713545 | Q6TDT1_HUMAN (Q6TDT1) Protein transactivated by hepatitis B virus E antigen, partial (34%) [THC2713545] | -3.26 |
| SYTL4 | Homo sapiens synaptotagmin-like 4 (granuphilin-a) (SYTL4), mRNA [NM_080737] | -3.26 |
| CD52 | Homo sapiens CD52 molecule (CD52), mRNA [NM_001803] | -3.25 |
| LOC730766 | PREDICTED: Homo sapiens similar to Peroxisomal coenzyme A diphosphatase NUDT7 (Nucleoside diphosphate-linked moiety X motif 7) (Nudix motif 7) (LOC730766), mRNA [XM_001127131] | -3.24 |
| RHBDF2 | Homo sapiens rhomboid 5 homolog 2 (Drosophila) (RHBDF2), transcript variant 1, mRNA [NM_024599] | -3.24 |
| SCRN3 | Homo sapiens secernin 3 (SCRN3), mRNA [NM_024583] | -3.23 |
| TREML2 | Homo sapiens triggering receptor expressed on myeloid cells-like 2 (TREML2), mRNA [NM_024807] | -3.23 |
| A_32_P57002 | Unknown | -3.22 |
| TANC1 | Homo sapiens tetratricopeptide repeat, ankyrin repeat and coiled-coil containing 1 (TANC1), mRNA [NM_033394] | -3.22 |
| WDSUB1 | Homo sapiens WD repeat, sterile alpha motif and U-box domain containing 1 (WDSUB1), mRNA [NM_152528] | -3.21 |
| CNOT6 | Homo sapiens CCR4-NOT transcription complex, subunit 6 (CNOT6), mRNA [NM_015455] | -3.20 |
| HIST3H2A | Homo sapiens histone cluster 3, H2a (HIST3H2A), mRNA [NM_033445] | -3.19 |
| CCDC24 | Homo sapiens coiled-coil domain containing 24 (CCDC24), mRNA [NM_152499] | -3.19 |
| THC2505770 | Unknown | -3.19 |
| CPNE3 | Homo sapiens copine III (CPNE3), mRNA [NM_003909] | -3.19 |
| HBLD1 | Homo sapiens HesB like domain containing 1 (HBLD1), mRNA [NM_194279] | -3.19 |
| THC2717907 | Q4IZP3_AZOVI (Q4IZP3) Outer membrane porin, partial (3%) [THC2717907] | -3.18 |
| EGLN3 | Homo sapiens egl nine homolog 3 (C. elegans) (EGLN3), mRNA [NM_022073] | -3.17 |
| PINK1 | Homo sapiens PTEN induced putative kinase 1 (PINK1), nuclear gene encoding mitochondrial protein, mRNA [NM_032409] | -3.17 |
| ROPN1L | Homo sapiens ropporin 1-like (ROPN1L), mRNA [NM_031916] | -3.15 |
| PLA2G4A | Homo sapiens phospholipase A2, group IVA (cytosolic, calcium-dependent) (PLA2G4A), mRNA [NM_024420] | -3.14 |
| LOC147645 | Homo sapiens, clone IMAGE:4401841, mRNA. [BC016993] | -3.14 |
| FLJ40722 | Homo sapiens hypothetical protein FLJ40722 (FLJ40722), mRNA [NM_173678] | -3.12 |
| C1orf172 | Homo sapiens chromosome 1 open reading frame 172 (C1orf172), mRNA [NM_152365] | -3.12 |
| FAM27E1 | Homo sapiens family with sequence similarity 27, member E1 (FAM27E1), mRNA [NM_001024608] | -3.12 |
| LEPROT | Homo sapiens leptin receptor overlapping transcript (LEPROT), mRNA [NM_017526] | -3.12 |
| A_32_P174385 | Unknown | -3.11 |
| RP11-151A6.2 | Homo sapiens hypothetical protein BC004360, mRNA (cDNA clone IMAGE:2822295), complete cds. [BC001077] | -3.11 |
| ATP6V1E2 | Homo sapiens ATPase, H+ transporting, lysosomal 31kDa, V1 subunit E2 (ATP6V1E2), mRNA [NM_080653] | -3.11 |
| HSPC268 | Homo sapiens hypothetical protein HSPC268 (HSPC268), mRNA [NM_197964] | -3.11 |
| PAQR6 | Homo sapiens progestin and adipoQ receptor family member VI (PAQR6), transcript variant 1, mRNA [NM_024897] | -3.11 |
| CTR9 | Homo sapiens Ctr9, Paf1/RNA polymerase II complex component, homolog (S. cerevisiae) (CTR9), mRNA [NM_014633] | -3.11 |
| AIM1L | Homo sapiens absent in melanoma 1-like (AIM1L), mRNA [NM_017977] | -3.10 |
| MANEAL | Homo sapiens mannosidase, endo-alpha-like (MANEAL), transcript variant 1, mRNA [NM_001031740] | -3.10 |
| C21orf119 | Homo sapiens chromosome 21 open reading frame 119 (C21orf119), mRNA [NM_032910] | -3.09 |
| AW268902 | AW268902 xv48h10.x1 Soares_NFL_T_GBC_S1 Homo sapiens cDNA clone IMAGE:2816419 3', mRNA sequence [AW268902] | -3.09 |
| HIST2H2AB | Homo sapiens histone cluster 2, H2ab (HIST2H2AB), mRNA [NM_175065] | -3.08 |
| HIST1H2AJ | Homo sapiens histone cluster 1, H2aj (HIST1H2AJ), mRNA [NM_021066] | -3.08 |
| FBXO25 | Homo sapiens F-box protein 25 (FBXO25), transcript variant 1, mRNA [NM_183421] | -3.08 |
| BU566406 | AGENCOURT_10396508 NIH_MGC_141 Homo sapiens cDNA clone IMAGE:6605580 5', mRNA sequence [BU566406] | -3.07 |
| CCNDBP1 | Homo sapiens cyclin D-type binding-protein 1 (CCNDBP1), transcript variant 2, mRNA [NM_037370] | -3.07 |
| CTH | Homo sapiens cystathionase (cystathionine gamma-lyase) (CTH), transcript variant 1, mRNA [NM_001902] | -3.07 |
| TMEM116 | Homo sapiens transmembrane protein 116 (TMEM116), mRNA [NM_138341] | -3.07 |
| THC2732746 | Unknown | -3.06 |
| TAC3 | Homo sapiens tachykinin 3 (neuromedin K, neurokinin beta) (TAC3), transcript variant 1, mRNA [NM_001006667] | -3.06 |
| TCEAL1 | Homo sapiens transcription elongation factor A (SII)-like 1 (TCEAL1), transcript variant 3, mRNA [NM_001006640] | -3.06 |
| DDEFL1 | Homo sapiens development and differentiation enhancing factor-like 1 (DDEFL1), mRNA [NM_017707] | -3.06 |
| ENST00000356177 | Histone H3.1 (H3/a) (H3/b) (H3/c) (H3/d) (H3/f) (H3/h) (H3/i) (H3/j) (H3/k) (H3/l). [Source:Uniprot/SWISSPROT;Acc:P68431] [ENST00000356177] | -3.06 |
| A_23_P170713 | Unknown | -3.05 |
| THC2616195 | Q569K0_HUMAN (Q569K0) ZNF599 protein, partial (84%) [THC2616195] | -3.05 |
| C3orf26 | Homo sapiens chromosome 3 open reading frame 26 (C3orf26), mRNA [NM_032359] | -3.04 |
| AI359640 | AI359640 qy33b12.x1 NCI_CGAP_Brn23 Homo sapiens cDNA clone IMAGE:2013791 3', mRNA sequence [AI359640] | -3.04 |
| HSP90AA2 | Homo sapiens heat shock protein 90kDa alpha (cytosolic), class A member 2 (HSP90AA2), mRNA [NM_001040141] | -3.03 |
| VPS13B | Homo sapiens vacuolar protein sorting 13 homolog B (yeast) (VPS13B), transcript variant 5, mRNA [NM_017890] | -3.03 |
| RABGAP1L | Homo sapiens RAB GTPase activating protein 1-like (RABGAP1L), transcript variant 1, mRNA [NM_014857] | -3.03 |
| BC073976 | Homo sapiens cDNA clone IMAGE:6018774, partial cds. [BC073976] | -3.03 |
| C14orf143 | Homo sapiens chromosome 14 open reading frame 143 (C14orf143), mRNA [NM_145231] | -3.02 |
| ASRGL1 | Homo sapiens asparaginase like 1, mRNA (cDNA clone IMAGE:3952485), complete cds. [BC006267] | -3.01 |
| MGC70863 | Homo sapiens similar to RPL23AP7 protein (MGC70863), transcript variant 1, mRNA [NM_203477] | -3.00 |
| CSTF3 | Homo sapiens cleavage stimulation factor, 3' pre-RNA, subunit 3, 77kDa (CSTF3), transcript variant 2, mRNA [NM_001033505] | -2.99 |
| HIST1H2AK | Homo sapiens histone cluster 1, H2ak (HIST1H2AK), mRNA [NM_003510] | -2.98 |
| A_24_P118472 | Unknown | -2.98 |
| BC019667 | Homo sapiens cDNA clone IMAGE:4453251, partial cds. [BC019667] | -2.98 |
| COL15A1 | Homo sapiens collagen, type XV, alpha 1 (COL15A1), mRNA [NM_001855] | -2.98 |
| NAGK | Homo sapiens N-acetylglucosamine kinase (NAGK), mRNA [NM_017567] | -2.97 |
| SAT2 | Homo sapiens spermidine/spermine N1-acetyltransferase 2 (SAT2), mRNA [NM_133491] | -2.97 |
| S100P | Homo sapiens S100 calcium binding protein P (S100P), mRNA [NM_005980] | -2.97 |
| DKFZp313A2432 | Homo sapiens mRNA; cDNA DKFZp313A2432 (from clone DKFZp313A2432). [AL833119] | -2.96 |
| TBL1Y | Homo sapiens transducin (beta)-like 1Y-linked (TBL1Y), transcript variant 1, mRNA [NM_033284] | -2.95 |
| SLC44A1 | Homo sapiens solute carrier family 44, member 1 (SLC44A1), mRNA [NM_080546] | -2.95 |
| HIST1H2AJ | Homo sapiens histone cluster 1, H2aj (HIST1H2AJ), mRNA [NM_021066] | -2.95 |
| AMOTL1 | Homo sapiens angiomotin like 1 (AMOTL1), mRNA [NM_130847] | -2.94 |
| ANXA4 | Homo sapiens annexin A4 (ANXA4), mRNA [NM_001153] | -2.94 |
| KIAA0329 | Homo sapiens KIAA0329 (KIAA0329), mRNA [NM_014844] | -2.93 |
| NME7 | Homo sapiens non-metastatic cells 7, protein expressed in (nucleoside-diphosphate kinase) (NME7), transcript variant 1, mRNA [NM_013330] | -2.93 |
| MRPL53 | Homo sapiens mitochondrial ribosomal protein L53 (MRPL53), nuclear gene encoding mitochondrial protein, mRNA [NM_053050] | -2.91 |
| SLC36A4 | Homo sapiens solute carrier family 36 (proton/amino acid symporter), member 4 (SLC36A4), mRNA [NM_152313] | -2.91 |
| OPTN | Homo sapiens optineurin (OPTN), transcript variant 1, mRNA [NM_001008211] | -2.90 |
| THC2538882 | Q5VT28_HUMAN (Q5VT28) Family with sequence similarity 27, member B (Family with sequence similarity 27, member A) (Family with sequence similarity 27, member C), partial (85%) [THC2538882] | -2.90 |
| TJP1 | Homo sapiens tight junction protein 1 (zona occludens 1) (TJP1), transcript variant 1, mRNA [NM_003257] | -2.89 |
| C11orf71 | Homo sapiens chromosome 11 open reading frame 71 (C11orf71), mRNA [NM_019021] | -2.89 |
| FER | Homo sapiens fer (fps/fes related) tyrosine kinase (phosphoprotein NCP94) (FER), mRNA [NM_005246] | -2.88 |
| DUSP13 | Homo sapiens dual specificity phosphatase 13 (DUSP13), transcript variant 1, mRNA [NM_001007271] | -2.88 |
| STARD9 | StAR-related lipid transfer protein 9 (StARD9) (START domain- containing protein 9) (Fragment). [Source:Uniprot/SWISSPROT;Acc:Q9P2P6] [ENST00000290607] | -2.87 |
| LOC255783 | Homo sapiens hypothetical protein LOC255783 (LOC255783) on chromosome 19 [NR_002797] | -2.87 |
| GPATCH2 | Homo sapiens G patch domain containing 2 (GPATCH2), mRNA [NM_018040] | -2.87 |
| C14orf179 | Homo sapiens chromosome 14 open reading frame 179 (C14orf179), mRNA [NM_052873] | -2.87 |
| ACRBP | Homo sapiens acrosin binding protein (ACRBP), mRNA [NM_032489] | -2.86 |
| POLE4 | Homo sapiens polymerase (DNA-directed), epsilon 4 (p12 subunit) (POLE4), mRNA [NM_019896] | -2.86 |
| C11orf71 | Homo sapiens chromosome 11 open reading frame 71 (C11orf71), mRNA [NM_019021] | -2.86 |
| LOC51255 | Homo sapiens hypothetical protein LOC51255 (LOC51255), mRNA [NM_016494] | -2.86 |
| A_32_P168727 | Unknown | -2.85 |
| XK | Homo sapiens X-linked Kx blood group (McLeod syndrome) (XK), mRNA [NM_021083] | -2.85 |
| HEMGN | Homo sapiens hemogen (HEMGN), transcript variant 1, mRNA [NM_018437] | -2.85 |
| POLE4 | Homo sapiens polymerase (DNA-directed), epsilon 4 (p12 subunit) (POLE4), mRNA [NM_019896] | -2.84 |
| TBL1X | Homo sapiens transducin (beta)-like 1X-linked (TBL1X), mRNA [NM_005647] | -2.84 |
| BG777521 | 602664870F1 NIH_MGC_60 Homo sapiens cDNA clone IMAGE:4804769 5', mRNA sequence [BG777521] | -2.83 |
| GSTT1 | Homo sapiens glutathione S-transferase theta 1 (GSTT1), mRNA [NM_000853] | -2.83 |
| LOC390705 | Homo sapiens similar to protein phosphatase 2A 48 kDa regulatory subunit isoform 1; serine/threonine protein phosphatase 2A, 48kDa regulatory subunit; PP2A, subunit B, PR48 isoform; PP2A B subunit PR48; NY-REN-8 antigen, mRNA (cDNA clone... | -2.83 |
| ENST00000357303 | CDNA FLJ37034 fis, clone BRACE2011478 (Hypothetical protein FLJ37034). [Source:Uniprot/SPTREMBL;Acc:Q8N9J7] [ENST00000357303] | -2.83 |
| C20orf142 | Homo sapiens chromosome 20 open reading frame 142, mRNA (cDNA clone IMAGE:4933017), with apparent retained intron. [BC029662] | -2.83 |
| BBS2 | Homo sapiens Bardet-Biedl syndrome 2 (BBS2), mRNA [NM_031885] | -2.82 |
| THC2655094 | XM_747355 succinyl-CoA synthetase {Aspergillus fumigatus Af293} (exp=-1; wgp=0; cg=0), partial (3%) [THC2655094] | -2.82 |
| THC2743390 | Unknown | -2.82 |
| SNHG10 | Homo sapiens small nucleolar RNA host gene (non-protein coding) 10 (SNHG10) on chromosome 14 [NR_003138] | -2.81 |
| MLLT11 | Homo sapiens myeloid/lymphoid or mixed-lineage leukemia (trithorax homolog, Drosophila); translocated to, 11 (MLLT11), mRNA [NM_006818] | -2.81 |
| LOC154822 | Homo sapiens cDNA FLJ43821 fis, clone TESTI4002290. [AK125809] | -2.81 |
| ENST00000373268 | Homo sapiens cDNA clone IMAGE:30416677. [BC090920] | -2.80 |
| CR624517 | full-length cDNA clone CS0DC002YA18 of Neuroblastoma Cot 25-normalized of Homo sapiens (human). [CR624517] | -2.80 |
| BRF1 | Homo sapiens BRF1 homolog, subunit of RNA polymerase III transcription initiation factor IIIB (S. cerevisiae), mRNA (cDNA clone IMAGE:4830425), complete cds. [BC016743] | -2.80 |
| SLCO2B1 | Homo sapiens solute carrier organic anion transporter family, member 2B1 (SLCO2B1), mRNA [NM_007256] | -2.80 |
| C2orf3 | Homo sapiens chromosome 2 open reading frame 3 (C2orf3), mRNA [NM_003203] | -2.79 |
| IMMP2L | Homo sapiens IMP2 inner mitochondrial membrane peptidase-like (S. cerevisiae) (IMMP2L), mRNA [NM_032549] | -2.79 |
| PNMA1 | Homo sapiens paraneoplastic antigen MA1 (PNMA1), mRNA [NM_006029] | -2.79 |
| DDT | Homo sapiens D-dopachrome tautomerase (DDT), mRNA [NM_001355] | -2.79 |
| HIST1H2AM | Homo sapiens histone cluster 1, H2am (HIST1H2AM), mRNA [NM_003514] | -2.79 |
| H2AFJ | Homo sapiens H2A histone family, member J (H2AFJ), transcript variant 2, mRNA [NM_177925] | -2.78 |
| COQ6 | Homo sapiens coenzyme Q6 homolog, monooxygenase (S. cerevisiae) (COQ6), transcript variant 1, mRNA [NM_182476] | -2.78 |
| ZNF626 | Homo sapiens zinc finger protein 626 (ZNF626), transcript variant 2, mRNA [NM_145297] | -2.77 |
| PHOSPHO2 | Homo sapiens phosphatase, orphan 2 (PHOSPHO2), mRNA [NM_001008489] | -2.77 |
| AX721087 | Sequence 47 from Patent WO0220754. [AX721087] | -2.76 |
| BC080624 | Homo sapiens cDNA clone MGC:99790 IMAGE:6304510, complete cds. [BC080624] | -2.76 |
| THC2757602 | Unknown | -2.76 |
| DUSP3 | Homo sapiens dual specificity phosphatase 3 (vaccinia virus phosphatase VH1-related) (DUSP3), mRNA [NM_004090] | -2.76 |
| WBP5 | Homo sapiens WW domain binding protein 5 (WBP5), transcript variant 1, mRNA [NM_016303] | -2.75 |
| C1orf53 | Homo sapiens chromosome 1 open reading frame 53 (C1orf53), mRNA [NM_001024594] | -2.75 |
| C12orf4 | Homo sapiens chromosome 12 open reading frame 4 (C12orf4), mRNA [NM_020374] | -2.75 |
| ROM1 | Homo sapiens retinal outer segment membrane protein 1 (ROM1), mRNA [NM_000327] | -2.74 |
| C14orf2 | Homo sapiens chromosome 14 open reading frame 2 (C14orf2), mRNA [NM_004894] | -2.74 |
| HARS2 | Homo sapiens histidyl-tRNA synthetase 2 (HARS2), mRNA [NM_080820] | -2.74 |
| NPAS1 | Homo sapiens neuronal PAS domain protein 1 (NPAS1), mRNA [NM_002517] | -2.74 |
| C17orf55 | Homo sapiens chromosome 17 open reading frame 55 (C17orf55), mRNA [NM_178519] | -2.73 |
| BQ071182 | BQ071182 AGENCOURT_6853099 NIH_MGC_47 Homo sapiens cDNA clone IMAGE:5927550 5', mRNA sequence [BQ071182] | -2.73 |
| IHPK2 | Homo sapiens inositol hexaphosphate kinase 2 (IHPK2), transcript variant 3, mRNA [NM_001005910] | -2.73 |
| PCBP4 | Homo sapiens poly(rC) binding protein 4 (PCBP4), transcript variant 4, mRNA [NM_033010] | -2.72 |
| LOC644656 | Homo sapiens cDNA clone IMAGE:5286302. [BC036621] | -2.72 |
| ID2 | Homo sapiens inhibitor of DNA binding 2, dominant negative helix-loop-helix protein (ID2), mRNA [NM_002166] | -2.72 |
| MGC70863 | Homo sapiens similar to RPL23AP7 protein (MGC70863), transcript variant 1, mRNA [NM_203477] | -2.72 |
| C5orf4 | Homo sapiens chromosome 5 open reading frame 4 (C5orf4), transcript variant 2, mRNA [NM_032385] | -2.71 |
| KREMEN1 | Homo sapiens kringle containing transmembrane protein 1 (KREMEN1), transcript variant 3, mRNA [NM_001039570] | -2.71 |
| ENST00000378770 | Heat shock protein 90Ad. [Source:Uniprot/SPTREMBL;Acc:Q58FG1] [ENST00000378770] | -2.71 |
| ZNF688 | Homo sapiens zinc finger protein 688 (ZNF688), transcript variant 1, mRNA [NM_145271] | -2.70 |
| TUBA2 | Homo sapiens tubulin, alpha 2 (TUBA2), transcript variant 2, mRNA [NM_079836] | -2.70 |
| TMEM157 | Homo sapiens transmembrane protein 157 (TMEM157), mRNA [NM_198507] | -2.70 |
| PSMD14 | Homo sapiens proteasome (prosome, macropain) 26S subunit, non-ATPase, 14 (PSMD14), mRNA [NM_005805] | -2.70 |
| THC2528011 | O46172_NEPCL (O46172) Dragline silk protein spidroin 1 (Fragment), partial (7%) [THC2528011] | -2.69 |
| ZFYVE1 | Homo sapiens zinc finger, FYVE domain containing 1 (ZFYVE1), transcript variant 1, mRNA [NM_021260] | -2.69 |
| SFT2D2 | Homo sapiens SFT2 domain containing 2 (SFT2D2), mRNA [NM_199344] | -2.69 |
| METT5D1 | Homo sapiens methyltransferase 5 domain containing 1 (METT5D1), mRNA [NM_152636] | -2.69 |
| MLSTD2 | Homo sapiens male sterility domain containing 2 (MLSTD2), mRNA [NM_032228] | -2.69 |
| PEX12 | Homo sapiens peroxisomal biogenesis factor 12 (PEX12), mRNA [NM_000286] | -2.68 |
| LHFPL2 | Homo sapiens lipoma HMGIC fusion partner-like 2 (LHFPL2), mRNA [NM_005779] | -2.68 |
| A_24_P745883 | Unknown | -2.68 |
| TMEM121 | Homo sapiens transmembrane protein 121 (TMEM121), mRNA [NM_025268] | -2.68 |
| C10orf83 | Homo sapiens chromosome 10 open reading frame 83, mRNA (cDNA clone IMAGE:4690584), complete cds. [BC022054] | -2.67 |
| GSTT1 | Homo sapiens glutathione S-transferase theta 1 (GSTT1), mRNA [NM_000853] | -2.67 |
| HIST1H2AB | Homo sapiens histone cluster 1, H2ab (HIST1H2AB), mRNA [NM_003513] | -2.67 |
| TP53TG3 | Homo sapiens TP53TG3 protein (TP53TG3), mRNA [NM_016212] | -2.67 |
| A_24_P147849 | Unknown | -2.67 |
| TCEAL8 | Homo sapiens transcription elongation factor A (SII)-like 8 (TCEAL8), transcript variant 1, mRNA [NM_153333] | -2.67 |
| MLSTD2 | Homo sapiens male sterility domain containing 2 (MLSTD2), mRNA [NM_032228] | -2.66 |
| STARD10 | Homo sapiens START domain containing 10 (STARD10), mRNA [NM_006645] | -2.66 |
| CK300181 | UI-E-EJ1-ajx-g-19-0-UI.s1 UI-E-EJ1 Homo sapiens cDNA clone UI-E-EJ1-ajx-g-19-0-UI 3', mRNA sequence [CK300181] | -2.66 |
| DSCR4 | Homo sapiens Down syndrome critical region gene 4 (DSCR4), mRNA [NM_005867] | -2.66 |
| UBE2H | Homo sapiens ubiquitin-conjugating enzyme E2H (UBC8 homolog, yeast) (UBE2H), transcript variant 1, mRNA [NM_003344] | -2.65 |
| URP2 | Homo sapiens UNC-112 related protein 2 (URP2), transcript variant URP2LF, mRNA [NM_178443] | -2.65 |
| MRPL53 | Homo sapiens mitochondrial ribosomal protein L53 (MRPL53), nuclear gene encoding mitochondrial protein, mRNA [NM_053050] | -2.65 |
| TAF10 | Homo sapiens TAF10 RNA polymerase II, TATA box binding protein (TBP)-associated factor, 30kDa (TAF10), mRNA [NM_006284] | -2.64 |
| THC2714497 | Q4T2S3_TETNG (Q4T2S3) Chromosome undetermined SCAF10199, whole genome shotgun sequence, partial (4%) [THC2652443] | -2.64 |
| PDLIM5 | Homo sapiens PDZ and LIM domain 5 (PDLIM5), transcript variant 1, mRNA [NM_006457] | -2.64 |
| C9orf23 | Homo sapiens chromosome 9 open reading frame 23 (C9orf23), transcript variant 2, mRNA [NM_148179] | -2.64 |
| TANC1 | Homo sapiens tetratricopeptide repeat, ankyrin repeat and coiled-coil containing 1 (TANC1), mRNA [NM_033394] | -2.64 |
| RPL23AP7 | Homo sapiens ribosomal protein L23a pseudogene 7 (RPL23AP7) on chromosome 2 [NR_000029] | -2.64 |
| POLD4 | Homo sapiens polymerase (DNA-directed), delta 4 (POLD4), mRNA [NM_021173] | -2.63 |
| HIST1H4K | Homo sapiens histone cluster 1, H4k (HIST1H4K), mRNA [NM_003541] | -2.63 |
| TUBB3 | Homo sapiens tubulin, beta 3 (TUBB3), mRNA [NM_006086] | -2.63 |
| NFU1 | Homo sapiens NFU1 iron-sulfur cluster scaffold homolog (S. cerevisiae) (NFU1), transcript variant 1, mRNA [NM_015700] | -2.62 |
| THC2506377 | HSU02032 ribosomal protein L23a {Homo sapiens} (exp=-1; wgp=0; cg=0), partial (92%) [THC2506377] | -2.62 |
| CCDC115 | Homo sapiens coiled-coil domain containing 115 (CCDC115), mRNA [NM_032357] | -2.62 |
| ALDH6A1 | Homo sapiens aldehyde dehydrogenase 6 family, member A1 (ALDH6A1), nuclear gene encoding mitochondrial protein, mRNA [NM_005589] | -2.62 |
| KCNMB3 | Homo sapiens potassium large conductance calcium-activated channel, subfamily M beta member 3 (KCNMB3), transcript variant 1, mRNA [NM_171828] | -2.62 |
| AK123450 | Homo sapiens cDNA FLJ41456 fis, clone BRSTN2012320. [AK123450] | -2.61 |
| GPRC5B | Homo sapiens G protein-coupled receptor, family C, group 5, member B (GPRC5B), mRNA [NM_016235] | -2.61 |
| EVL | Homo sapiens Enah/Vasp-like (EVL), mRNA [NM_016337] | -2.61 |
| TMEM50A | Homo sapiens transmembrane protein 50A (TMEM50A), mRNA [NM_014313] | -2.61 |
| A_23_P392897 | Unknown | -2.61 |
| FAM13A1 | Homo sapiens family with sequence similarity 13, member A1 (FAM13A1), transcript variant 1, mRNA [NM_014883] | -2.61 |
| BPHL | Homo sapiens biphenyl hydrolase-like (serine hydrolase; breast epithelial mucin-associated antigen) (BPHL), mRNA [NM_004332] | -2.61 |
| HIST1H4J | Homo sapiens histone cluster 1, H4j (HIST1H4J), mRNA [NM_021968] | -2.60 |
| ANXA3 | Homo sapiens annexin A3 (ANXA3), mRNA [NM_005139] | -2.60 |
| UNQ501 | Homo sapiens MBC3205 (UNQ501), mRNA [NM_198536] | -2.60 |
| SAAL1 | Homo sapiens serum amyloid A-like 1 (SAAL1), mRNA [NM_138421] | -2.60 |
| GLS | Homo sapiens glutaminase (GLS), mRNA [NM_014905] | -2.60 |
| NUDT16 | Homo sapiens nudix (nucleoside diphosphate linked moiety X)-type motif 16 (NUDT16), mRNA [NM_152395] | -2.60 |
| MRPL35 | Homo sapiens mitochondrial ribosomal protein L35 (MRPL35), nuclear gene encoding mitochondrial protein, transcript variant 2, mRNA [NM_145644] | -2.59 |
| PPP2R5C | Homo sapiens protein phosphatase 2, regulatory subunit B (B56), gamma isoform (PPP2R5C), transcript variant 4, mRNA [NM_178588] | -2.59 |
| ENST00000292140 | PHLDB3 protein (Fragment). [Source:Uniprot/SPTREMBL;Acc:Q96HZ0] [ENST00000292140] | -2.58 |
| RAB3D | Homo sapiens RAB3D, member RAS oncogene family, mRNA (cDNA clone IMAGE:4301650), partial cds. [BC007960] | -2.58 |
| SCN9A | Homo sapiens sodium channel, voltage-gated, type IX, alpha (SCN9A), mRNA [NM_002977] | -2.58 |
| C4orf32 | Homo sapiens chromosome 4 open reading frame 32 (C4orf32), mRNA [NM_152400] | -2.58 |
| ATXN3 | Homo sapiens ataxin 3 (ATXN3), transcript variant 1, mRNA [NM_004993] | -2.58 |
| LDOC1L | Homo sapiens leucine zipper, down-regulated in cancer 1-like (LDOC1L), mRNA [NM_032287] | -2.58 |
| FLJ35880 | Homo sapiens hypothetical protein FLJ35880 (FLJ35880), mRNA [NM_153264] | -2.58 |
| FLJ20021 | Homo sapiens cDNA FLJ20021 fis, clone ADSE01233. [AK000028] | -2.57 |
| DDR1 | Homo sapiens discoidin domain receptor family, member 1 (DDR1), transcript variant 1, mRNA [NM_013993] | -2.57 |
| NDUFB1 | Homo sapiens NADH dehydrogenase (ubiquinone) 1 beta subcomplex, 1, 7kDa (NDUFB1), mRNA [NM_004545] | -2.57 |
| LOC646561 | PREDICTED: Homo sapiens similar to WW45 protein (LOC646561), mRNA [XM_929496] | -2.56 |
| MRPL17 | Homo sapiens mitochondrial ribosomal protein L17 (MRPL17), nuclear gene encoding mitochondrial protein, mRNA [NM_022061] | -2.56 |
| PEX14 | Homo sapiens peroxisomal biogenesis factor 14, mRNA (cDNA clone IMAGE:4696946), partial cds. [BC017848] | -2.56 |
| MLSTD2 | Homo sapiens male sterility domain containing 2 (MLSTD2), mRNA [NM_032228] | -2.56 |
| ADCK2 | Homo sapiens aarF domain containing kinase 2 (ADCK2), mRNA [NM_052853] | -2.55 |
| KIAA0284 | Homo sapiens KIAA0284 (KIAA0284), mRNA [NM_015005] | -2.55 |
| ZNF485 | Homo sapiens zinc finger protein 485 (ZNF485), mRNA [NM_145312] | -2.55 |
| CTBS | Homo sapiens chitobiase, di-N-acetyl- (CTBS), mRNA [NM_004388] | -2.55 |
| THC2774217 | AF264779 sporulation-induced transcript 4-associated protein SAPLa {Homo sapiens} (exp=-1; wgp=0; cg=0), partial (3%) [THC2774217] | -2.55 |
| THC2704973 | Unknown | -2.55 |
| SLC36A4 | Homo sapiens solute carrier family 36 (proton/amino acid symporter), member 4 (SLC36A4), mRNA [NM_152313] | -2.55 |
| SOCS3 | Homo sapiens suppressor of cytokine signaling 3 (SOCS3), mRNA [NM_003955] | -2.55 |
| ENST00000324677 | Protein phosphatase Slingshot homolog 2 (EC 3.1.3.48) (EC 3.1.3.16) (SSH-2L) (hSSH-2L). [Source:Uniprot/SWISSPROT;Acc:Q76I76] [ENST00000324677] | -2.55 |
| DLL3 | Homo sapiens delta-like 3 (Drosophila) (DLL3), transcript variant 2, mRNA [NM_203486] | -2.55 |
| THC2565393 | Q5VT28_HUMAN (Q5VT28) Family with sequence similarity 27, member B (Family with sequence similarity 27, member A) (Family with sequence similarity 27, member C), partial (81%) [THC2565393] | -2.55 |
| EPPB9 | Homo sapiens B9 protein (EPPB9), mRNA [NM_015681] | -2.55 |
| GNG10 | Homo sapiens guanine nucleotide binding protein (G protein), gamma 10 (GNG10), mRNA [NM_001017998] | -2.55 |
| C14orf103 | Homo sapiens chromosome 14 open reading frame 103 (C14orf103), mRNA [NM_018036] | -2.54 |
| PGEA1 | Homo sapiens PKD2 interactor, golgi and endoplasmic reticulum associated 1 (PGEA1), transcript variant 2, mRNA [NM_001002880] | -2.54 |
| DCP2 | Homo sapiens DCP2 decapping enzyme homolog (S. cerevisiae) (DCP2), mRNA [NM_152624] | -2.53 |
| TUBB2A | Homo sapiens tubulin, beta 2A (TUBB2A), mRNA [NM_001069] | -2.53 |
| ZADH1 | Homo sapiens zinc binding alcohol dehydrogenase, domain containing 1 (ZADH1), mRNA [NM_152444] | -2.52 |
| AMOTL1 | Homo sapiens angiomotin like 1 (AMOTL1), mRNA [NM_130847] | -2.52 |
| MRPL19 | Homo sapiens mitochondrial ribosomal protein L19 (MRPL19), nuclear gene encoding mitochondrial protein, mRNA [NM_014763] | -2.52 |
| SPATA20 | Homo sapiens spermatogenesis associated 20 (SPATA20), mRNA [NM_022827] | -2.51 |
| DYSF | Homo sapiens dysferlin, limb girdle muscular dystrophy 2B (autosomal recessive) (DYSF), mRNA [NM_003494] | -2.51 |
| SMYD3 | Homo sapiens SET and MYND domain containing 3 (SMYD3), mRNA [NM_022743] | -2.51 |
| GAA | Homo sapiens glucosidase, alpha; acid (Pompe disease, glycogen storage disease type II) (GAA), transcript variant 1, mRNA [NM_000152] | -2.51 |
| ANXA1 | Homo sapiens annexin A1 (ANXA1), mRNA [NM_000700] | -2.51 |
| CSNK1G3 | Homo sapiens casein kinase 1, gamma 3 (CSNK1G3), transcript variant 4, mRNA [NM_001044723] | -2.51 |
| CXorf38 | Homo sapiens chromosome X open reading frame 38 (CXorf38), mRNA [NM_144970] | -2.50 |
| HDHD1A | Homo sapiens haloacid dehalogenase-like hydrolase domain containing 1A (HDHD1A), mRNA [NM_012080] | -2.50 |
| VAMP8 | Homo sapiens vesicle-associated membrane protein 8 (endobrevin) (VAMP8), mRNA [NM_003761] | -2.50 |
| C6orf1 | Homo sapiens chromosome 6 open reading frame 1 (C6orf1), transcript variant 1, mRNA [NM_178508] | -2.50 |
| C6orf64 | Homo sapiens chromosome 6 open reading frame 64 (C6orf64), mRNA [NM_018322] | -2.50 |
| FLJ21986 | Homo sapiens hypothetical protein FLJ21986 (FLJ21986), mRNA [NM_024913] | -2.50 |
| METTL8 | Homo sapiens methyltransferase like 8 (METTL8), mRNA [NM_024770] | -2.50 |
| RPLP2 | Homo sapiens ribosomal protein, large, P2 (RPLP2), mRNA [NM_001004] | -2.49 |
| C14orf133 | Homo sapiens chromosome 14 open reading frame 133 (C14orf133), mRNA [NM_022067] | -2.49 |
| ENST00000379855 | OTTHUMP00000021181. [Source:Uniprot/SPTREMBL;Acc:Q5VYT7] [ENST00000379855] | -2.49 |
| NUDT14 | Homo sapiens nudix (nucleoside diphosphate linked moiety X)-type motif 14 (NUDT14), mRNA [NM_177533] | -2.48 |
| CD59 | Homo sapiens CD59 molecule, complement regulatory protein (CD59), transcript variant 1, mRNA [NM_203330] | -2.48 |
| DACT3 | Homo sapiens dapper, antagonist of beta-catenin, homolog 3 (Xenopus laevis) (DACT3), mRNA [NM_145056] | -2.48 |
| HIST1H2AH | Homo sapiens histone cluster 1, H2ah (HIST1H2AH), mRNA [NM_080596] | -2.48 |
| GLRX5 | Homo sapiens glutaredoxin 5 homolog (S. cerevisiae) (GLRX5), mRNA [NM_016417] | -2.48 |
| POLD4 | Homo sapiens polymerase (DNA-directed), delta 4 (POLD4), mRNA [NM_021173] | -2.47 |
| PTPRS | Homo sapiens protein tyrosine phosphatase, receptor type, S (PTPRS), transcript variant 3, mRNA [NM_130853] | -2.47 |
| COX6A2 | Homo sapiens cytochrome c oxidase subunit VIa polypeptide 2 (COX6A2), nuclear gene encoding mitochondrial protein, mRNA [NM_005205] | -2.47 |
| RAB11FIP1 | Homo sapiens RAB11 family interacting protein 1 (class I) (RAB11FIP1), transcript variant 2, mRNA [NM_001002233] | -2.47 |
| AK057740 | Homo sapiens cDNA FLJ25011 fis, clone CBL01244. [AK057740] | -2.47 |
| ENST00000333546 | PREDICTED: Homo sapiens similar to 60S ribosomal protein L23a (LOC391282), mRNA [XM_372878] | -2.47 |
| TP53TG3 | Homo sapiens TP53TG3 protein (TP53TG3), mRNA [NM_016212] | -2.47 |
| TMEM150 | Homo sapiens transmembrane protein 150 (TMEM150), transcript variant 2, mRNA [NM_153342] | -2.46 |
| RHD | Homo sapiens Rh blood group, D antigen (RHD), mRNA [NM_016124] | -2.46 |
| TANK | Homo sapiens TRAF family member-associated NFKB activator (TANK), transcript variant 2, mRNA [NM_133484] | -2.45 |
| THC2613527 | Unknown | -2.45 |
| LOC728996 | PREDICTED: Homo sapiens similar to methyltransferase like 2 (LOC728996), mRNA [XR_015425] | -2.45 |
| AHRR | Homo sapiens aryl-hydrocarbon receptor repressor (AHRR), mRNA [NM_020731] | -2.45 |
| FLJ21127 | Homo sapiens tectonic (FLJ21127), mRNA [NM_024549] | -2.45 |
| EMP2 | Homo sapiens epithelial membrane protein 2 (EMP2), mRNA [NM_001424] | -2.45 |
| DAB2 | Homo sapiens disabled homolog 2, mitogen-responsive phosphoprotein (Drosophila) (DAB2), mRNA [NM_001343] | -2.45 |
| POLR2L | Homo sapiens polymerase (RNA) II (DNA directed) polypeptide L, 7.6kDa (POLR2L), mRNA [NM_021128] | -2.44 |
| THC2559002 | Unknown | -2.44 |
| PCK2 | Homo sapiens phosphoenolpyruvate carboxykinase 2 (mitochondrial) (PCK2), nuclear gene encoding mitochondrial protein, transcript variant 1, mRNA [NM_004563] | -2.43 |
| SPA17 | Homo sapiens sperm autoantigenic protein 17 (SPA17), mRNA [NM_017425] | -2.43 |
| TMPRSS5 | Homo sapiens transmembrane protease, serine 5 (spinesin) (TMPRSS5), mRNA [NM_030770] | -2.43 |
| PPP2R5C | Homo sapiens protein phosphatase 2, regulatory subunit B (B56), gamma isoform (PPP2R5C), transcript variant 1, mRNA [NM_002719] | -2.42 |
| CSRP2 | Homo sapiens cysteine and glycine-rich protein 2 (CSRP2), mRNA [NM_001321] | -2.42 |
| TMEM51 | Homo sapiens transmembrane protein 51 (TMEM51), mRNA [NM_018022] | -2.42 |
| STAM2 | Homo sapiens signal transducing adaptor molecule (SH3 domain and ITAM motif) 2 (STAM2), mRNA [NM_005843] | -2.42 |
| STEAP1 | Homo sapiens six transmembrane epithelial antigen of the prostate 1 (STEAP1), mRNA [NM_012449] | -2.42 |
| CLEC11A | Homo sapiens C-type lectin domain family 11, member A (CLEC11A), mRNA [NM_002975] | -2.42 |
| ELP4 | Homo sapiens elongation protein 4 homolog (S. cerevisiae) (ELP4), mRNA [NM_019040] | -2.42 |
| TMEM107 | Homo sapiens transmembrane protein 107 (TMEM107), transcript variant 1, mRNA [NM_032354] | -2.41 |
| LOC619208 | Homo sapiens hypothetical protein LOC619208 (LOC619208), mRNA [NM_001033564] | -2.41 |
| CLDN7 | Homo sapiens claudin 7 (CLDN7), mRNA [NM_001307] | -2.41 |
| NEU1 | Homo sapiens sialidase 1 (lysosomal sialidase) (NEU1), mRNA [NM_000434] | -2.41 |
| TNXB | Homo sapiens tenascin XB (TNXB), transcript variant XB-S, mRNA [NM_032470] | -2.41 |
| IFI30 | Homo sapiens interferon, gamma-inducible protein 30 (IFI30), mRNA [NM_006332] | -2.41 |
| RY1 | Homo sapiens putative nucleic acid binding protein RY-1 (RY1), mRNA [NM_006857] | -2.41 |
| PPP3CA | Homo sapiens protein phosphatase 3 (formerly 2B), catalytic subunit, alpha isoform (calcineurin A alpha) (PPP3CA), mRNA [NM_000944] | -2.41 |
| BC030106 | Homo sapiens cDNA clone IMAGE:4797878. [BC030106] | -2.41 |
| SLC39A13 | Homo sapiens solute carrier family 39 (zinc transporter), member 13 (SLC39A13), mRNA [NM_152264] | -2.41 |
| THC2700145 | Q9F8M7_CARHY (Q9F8M7) DTDP-glucose 4,6-dehydratase (Fragment), partial (11%) [THC2700145] | -2.40 |
| FLJ22639 | Homo sapiens hypothetical protein FLJ22639, mRNA (cDNA clone MGC:22172 IMAGE:4700838), complete cds. [BC017762] | -2.40 |
| SLC35A3 | Homo sapiens solute carrier family 35 (UDP-N-acetylglucosamine (UDP-GlcNAc) transporter), member A3 (SLC35A3), mRNA [NM_012243] | -2.39 |
| PEX13 | Homo sapiens peroxisome biogenesis factor 13 (PEX13), mRNA [NM_002618] | -2.39 |
| ZFAND3 | Homo sapiens zinc finger, AN1-type domain 3 (ZFAND3), mRNA [NM_021943] | -2.38 |
| ENST00000356572 | FAM39B protein. [Source:Uniprot/SPTREMBL;Acc:Q6GMS0] [ENST00000356572] | -2.38 |
| ENST00000328711 | Homo sapiens chromosome 13 open reading frame 29, mRNA (cDNA clone MGC:120939 IMAGE:7939749), complete cds. [BC093904] | -2.38 |
| MVP | Homo sapiens major vault protein (MVP), transcript variant 1, mRNA [NM_017458] | -2.38 |
| FGD6 | Homo sapiens FYVE, RhoGEF and PH domain containing 6 (FGD6), mRNA [NM_018351] | -2.38 |
| FAHD2A | Homo sapiens fumarylacetoacetate hydrolase domain containing 2A (FAHD2A), mRNA [NM_016044] | -2.37 |
| AMZ2 | Homo sapiens archaemetzincins-2 (AMZ2), transcript variant 1, mRNA [NM_016627] | -2.37 |
| MRPL35 | Homo sapiens mitochondrial ribosomal protein L35 (MRPL35), nuclear gene encoding mitochondrial protein, transcript variant 2, mRNA [NM_145644] | -2.37 |
| CR613972 | full-length cDNA clone CS0DI009YA14 of Placenta Cot 25-normalized of Homo sapiens (human). [CR613972] | -2.37 |
| GK | Homo sapiens glycerol kinase (GK), transcript variant 1, mRNA [NM_203391] | -2.37 |
| BATF2 | Homo sapiens basic leucine zipper transcription factor, ATF-like 2 (BATF2), mRNA [NM_138456] | -2.37 |
| MORN2 | Homo sapiens MORN repeat containing 2 (MORN2), mRNA [NM_194270] | -2.37 |
| THC2526647 | AV708150 AV708150 ADC Homo sapiens cDNA clone ADCAFF01 5', mRNA sequence [AV708150] | -2.37 |
| PARC | Homo sapiens p53-associated parkin-like cytoplasmic protein (PARC), mRNA [NM_015089] | -2.37 |
| IL10RA | Homo sapiens interleukin 10 receptor, alpha (IL10RA), mRNA [NM_001558] | -2.37 |
| SH3BP5 | Homo sapiens SH3-domain binding protein 5 (BTK-associated) (SH3BP5), transcript variant 1, mRNA [NM_004844] | -2.36 |
| CR625518 | full-length cDNA clone CS0DL012YA17 of B cells (Ramos cell line) Cot 25-normalized of Homo sapiens (human). [CR625518] | -2.36 |
| HIST1H2BK | Homo sapiens histone cluster 1, H2bk (HIST1H2BK), mRNA [NM_080593] | -2.36 |
| FUCA1 | Homo sapiens fucosidase, alpha-L- 1, tissue (FUCA1), mRNA [NM_000147] | -2.36 |
| IMMT | Homo sapiens inner membrane protein, mitochondrial (mitofilin) (IMMT), mRNA [NM_006839] | -2.36 |
| ZDHHC24 | Homo sapiens zinc finger, DHHC-type containing 24 (ZDHHC24), mRNA [NM_207340] | -2.35 |
| ASMTL | Homo sapiens acetylserotonin O-methyltransferase-like (ASMTL), mRNA [NM_004192] | -2.35 |
| TPRKB | Homo sapiens TP53RK binding protein (TPRKB), mRNA [NM_016058] | -2.35 |
| HDGFRP3 | Homo sapiens hepatoma-derived growth factor, related protein 3 (HDGFRP3), mRNA [NM_016073] | -2.35 |
| HIST1H2AC | Histone H2A type 1-C. [Source:Uniprot/SWISSPROT;Acc:Q93077] [ENST00000314088] | -2.34 |
| GFPT1 | Homo sapiens glutamine-fructose-6-phosphate transaminase 1 (GFPT1), mRNA [NM_002056] | -2.34 |
| LACTB2 | Homo sapiens lactamase, beta 2 (LACTB2), mRNA [NM_016027] | -2.34 |
| SEC61A2 | Homo sapiens Sec61 alpha 2 subunit (S. cerevisiae) (SEC61A2), mRNA [NM_018144] | -2.34 |
| PAM | Homo sapiens peptidylglycine alpha-amidating monooxygenase (PAM), transcript variant 1, mRNA [NM_000919] | -2.34 |
| ZSCAN2 | Homo sapiens zinc finger and SCAN domain containing 2 (ZSCAN2), transcript variant 2, mRNA [NM_017894] | -2.34 |
| PPP2R3B | Homo sapiens protein phosphatase 2 (formerly 2A), regulatory subunit B'', beta (PPP2R3B), transcript variant 1, mRNA [NM_013239] | -2.34 |
| A_24_P401150 | Unknown | -2.34 |
| ZDHHC13 | Homo sapiens zinc finger, DHHC-type containing 13 (ZDHHC13), transcript variant 1, mRNA [NM_019028] | -2.34 |
| C20orf19 | Homo sapiens chromosome 20 open reading frame 19 (C20orf19), mRNA [NM_018474] | -2.33 |
| HMGCL | Homo sapiens 3-hydroxymethyl-3-methylglutaryl-Coenzyme A lyase (hydroxymethylglutaricaciduria) (HMGCL), mRNA [NM_000191] | -2.33 |
| LOC145783 | full-length cDNA clone CS0DF019YP12 of Fetal brain of Homo sapiens (human). [CR612225] | -2.33 |
| BC029473 | Homo sapiens cDNA clone IMAGE:4723680, **** WARNING: chimeric clone ****. [BC029473] | -2.33 |
| A_23_P251002 | Unknown | -2.33 |
| HCCS | Homo sapiens holocytochrome c synthase (cytochrome c heme-lyase) (HCCS), mRNA [NM_005333] | -2.33 |
| A_32_P93584 | Unknown | -2.33 |
| HIST1H2BD | AGENCOURT_8209273 NIH_MGC_112 Homo sapiens cDNA clone IMAGE:6265521 5', mRNA sequence [BQ683489] | -2.32 |
| NDNL2 | Homo sapiens necdin-like 2 (NDNL2), mRNA [NM_138704] | -2.32 |
| FBXO25 | Homo sapiens F-box protein 25 (FBXO25), transcript variant 2, mRNA [NM_183420] | -2.32 |
| MAP1D | Homo sapiens methionine aminopeptidase 1D (MAP1D), mRNA [NM_199227] | -2.32 |
| RMND5A | Homo sapiens required for meiotic nuclear division 5 homolog A (S. cerevisiae) (RMND5A), mRNA [NM_022780] | -2.32 |
| CDC42BPB | Homo sapiens CDC42 binding protein kinase beta (DMPK-like) (CDC42BPB), mRNA [NM_006035] | -2.32 |
| COX15 | Homo sapiens COX15 homolog, cytochrome c oxidase assembly protein (yeast) (COX15), nuclear gene encoding mitochondrial protein, transcript variant 1, mRNA [NM_078470] | -2.32 |
| TRPV2 | Homo sapiens transient receptor potential cation channel, subfamily V, member 2 (TRPV2), mRNA [NM_016113] | -2.32 |
| A_24_P346859 | Unknown | -2.32 |
| HIST1H3H | Homo sapiens histone cluster 1, H3h (HIST1H3H), mRNA [NM_003536] | -2.32 |
| NP | Homo sapiens nucleoside phosphorylase (NP), mRNA [NM_000270] | -2.32 |
| WWOX | Homo sapiens WW domain containing oxidoreductase (WWOX), transcript variant 3, mRNA [NM_130844] | -2.31 |
| CRYAB | Homo sapiens crystallin, alpha B (CRYAB), mRNA [NM_001885] | -2.31 |
| RFFL | Homo sapiens ring finger and FYVE-like domain containing 1 (RFFL), transcript variant 1, mRNA [NM_057178] | -2.31 |
| GGCX | Homo sapiens gamma-glutamyl carboxylase (GGCX), mRNA [NM_000821] | -2.31 |
| HIST1H3D | Homo sapiens histone cluster 1, H3d (HIST1H3D), mRNA [NM_003530] | -2.31 |
| THC2541678 | IF2_STRAW (Q82K53) Translation initiation factor IF-2, partial (4%) [THC2541678] | -2.31 |
| C14orf129 | Homo sapiens chromosome 14 open reading frame 129 (C14orf129), mRNA [NM_016472] | -2.31 |
| ROD1 | Homo sapiens ROD1 regulator of differentiation 1 (S. pombe) (ROD1), mRNA [NM_005156] | -2.31 |
| STEAP1 | Homo sapiens six transmembrane epithelial antigen of the prostate 1 (STEAP1), mRNA [NM_012449] | -2.31 |
| TMEM136 | Homo sapiens transmembrane protein 136 (TMEM136), mRNA [NM_174926] | -2.30 |
| CCDC115 | Homo sapiens coiled-coil domain containing 115 (CCDC115), mRNA [NM_032357] | -2.30 |
| C17orf61 | Homo sapiens chromosome 17 open reading frame 61 (C17orf61), mRNA [NM_152766] | -2.30 |
| GALK1 | Homo sapiens galactokinase 1 (GALK1), mRNA [NM_000154] | -2.30 |
| C6orf85 | Homo sapiens chromosome 6 open reading frame 85, mRNA (cDNA clone IMAGE:3846727), complete cds. [BC022217] | -2.30 |
| MOBKL2B | Homo sapiens MOB1, Mps One Binder kinase activator-like 2B (yeast) (MOBKL2B), mRNA [NM_024761] | -2.29 |
| ZNF643 | Homo sapiens zinc finger protein 643 (ZNF643), mRNA [NM_023070] | -2.29 |
| ID2 | Homo sapiens inhibitor of DNA binding 2, dominant negative helix-loop-helix protein (ID2), mRNA [NM_002166] | -2.29 |
| ZNF778 | CDNA FLJ31875 fis, clone NT2RP7002450, weakly similar to ZINC FINGER PROTEIN 84. [Source:Uniprot/SPTREMBL;Acc:Q96MU6] [ENST00000306502] | -2.29 |
| LOC120379 | Homo sapiens hypothetical protein BC019238 (LOC120379), mRNA [NM_138789] | -2.29 |
| CR601496 | full-length cDNA clone CS0DI062YF05 of Placenta Cot 25-normalized of Homo sapiens (human). [CR601496] | -2.29 |
| HIST1H2AG | Homo sapiens histone cluster 1, H2ag (HIST1H2AG), mRNA [NM_021064] | -2.28 |
| THC2619545 | BC001305 ELOVL6 protein {Homo sapiens} (exp=-1; wgp=0; cg=0), partial (11%) [THC2619545] | -2.28 |
| HIST3H2BB | DB010344 TCOLN2 Homo sapiens cDNA clone TCOLN2001340 5', mRNA sequence [DB010344] | -2.28 |
| SCD | Homo sapiens stearoyl-CoA desaturase (delta-9-desaturase) (SCD), mRNA [NM_005063] | -2.28 |
| NINJ2 | Homo sapiens ninjurin 2 (NINJ2), mRNA [NM_016533] | -2.28 |
| SLC13A3 | Homo sapiens solute carrier family 13 (sodium-dependent dicarboxylate transporter), member 3 (SLC13A3), transcript variant 2, mRNA [NM_001011554] | -2.28 |
| AP3D1 | Homo sapiens adaptor-related protein complex 3, delta 1 subunit (AP3D1), transcript variant 2, mRNA [NM_003938] | -2.28 |
| LOC387763 | Homo sapiens hypothetical LOC387763, mRNA (cDNA clone IMAGE:6272440), partial cds. [BC052560] | -2.27 |
| CCDC53 | Homo sapiens coiled-coil domain containing 53 (CCDC53), mRNA [NM_016053] | -2.27 |
| REEP5 | Homo sapiens receptor accessory protein 5 (REEP5), mRNA [NM_005669] | -2.27 |
| C14orf112 | Homo sapiens chromosome 14 open reading frame 112 (C14orf112), mRNA [NM_016468] | -2.27 |
| TRIM6 | Homo sapiens tripartite motif-containing 6 (TRIM6), transcript variant 1, mRNA [NM_001003818] | -2.27 |
| MLLT3 | Homo sapiens myeloid/lymphoid or mixed-lineage leukemia (trithorax homolog, Drosophila); translocated to, 3 (MLLT3), mRNA [NM_004529] | -2.27 |
| RAB27A | Homo sapiens RAB27A, member RAS oncogene family (RAB27A), transcript variant 1, mRNA [NM_004580] | -2.27 |
| DOCK11 | Homo sapiens dedicator of cytokinesis 11 (DOCK11), mRNA [NM_144658] | -2.27 |
| GGTLA4 | Homo sapiens gamma-glutamyltransferase-like activity 4 (GGTLA4), transcript variant C, mRNA [NM_080920] | -2.27 |
| BM474343 | BM474343 AGENCOURT_6489981 NIH_MGC_71 Homo sapiens cDNA clone IMAGE:5521645 5', mRNA sequence [BM474343] | -2.26 |
| ZNF626 | Homo sapiens zinc finger protein 626 (ZNF626), transcript variant 2, mRNA [NM_145297] | -2.26 |
| AK054718 | Homo sapiens cDNA FLJ30156 fis, clone BRACE2000487. [AK054718] | -2.26 |
| ENST00000356931 | MGC10850 protein. [Source:Uniprot/SPTREMBL;Acc:Q96C21] [ENST00000356931] | -2.26 |
| Z69892 | H.sapiens mRNA (clone ICRFp507I1077). [Z69892] | -2.26 |
| CCNL2 | Homo sapiens cyclin L2 (CCNL2), transcript variant 2, mRNA [NM_001039577] | -2.25 |
| KIT | Homo sapiens v-kit Hardy-Zuckerman 4 feline sarcoma viral oncogene homolog (KIT), mRNA [NM_000222] | -2.25 |
| FEM1B | Homo sapiens fem-1 homolog b (C. elegans) (FEM1B), mRNA [NM_015322] | -2.25 |
| LDOC1L | Homo sapiens leucine zipper, down-regulated in cancer 1-like (LDOC1L), mRNA [NM_032287] | -2.25 |
| C14orf80 | Homo sapiens chromosome 14 open reading frame 80 (C14orf80), mRNA [NM_173608] | -2.25 |
| A_23_P216071 | Unknown | -2.25 |
| SF3B14 | Homo sapiens splicing factor 3B, 14 kDa subunit (SF3B14), mRNA [NM_016047] | -2.25 |
| ENST00000369158 | Histone H3.2 (H3/m) (H3/o). [Source:Uniprot/SWISSPROT;Acc:Q71DI3] [ENST00000369158] | -2.24 |
| CCDC25 | Homo sapiens coiled-coil domain containing 25 (CCDC25), mRNA [NM_018246] | -2.24 |
| TBC1D3 | Homo sapiens TBC1 domain family, member 3 (TBC1D3), mRNA [NM_032258] | -2.24 |
| FAM14B | Homo sapiens family with sequence similarity 14, member B (FAM14B), mRNA [NM_206949] | -2.24 |
| UBE2H | Homo sapiens ubiquitin-conjugating enzyme E2H (UBC8 homolog, yeast) (UBE2H), transcript variant 1, mRNA [NM_003344] | -2.24 |
| MSI2 | Homo sapiens musashi homolog 2 (Drosophila) (MSI2), transcript variant 2, mRNA [NM_170721] | -2.23 |
| LYSMD2 | Homo sapiens LysM, putative peptidoglycan-binding, domain containing 2 (LYSMD2), mRNA [NM_153374] | -2.23 |
| HIST1H2BB | Homo sapiens histone cluster 1, H2bb (HIST1H2BB), mRNA [NM_021062] | -2.23 |
| GMCL1 | Homo sapiens germ cell-less homolog 1 (Drosophila) (GMCL1), mRNA [NM_178439] | -2.23 |
| BX956036 | DKFZp781J1775_r1 781 (synonym: hlcc4) Homo sapiens cDNA clone DKFZp781J1775 5', mRNA sequence [BX956036] | -2.23 |
| HIST1H2BO | Homo sapiens histone cluster 1, H2bo (HIST1H2BO), mRNA [NM_003527] | -2.23 |
| SCAND1 | Homo sapiens SCAN domain containing 1 (SCAND1), transcript variant 1, mRNA [NM_016558] | -2.22 |
| FZD1 | Homo sapiens frizzled homolog 1 (Drosophila) (FZD1), mRNA [NM_003505] | -2.22 |
| ENST00000318291 | Homo sapiens, clone IMAGE:3621928, mRNA, partial cds. [BC033133] | -2.22 |
| LACTB | Homo sapiens lactamase, beta (LACTB), nuclear gene encoding mitochondrial protein, transcript variant 2, mRNA [NM_171846] | -2.22 |
| PROCR | Homo sapiens protein C receptor, endothelial (EPCR) (PROCR), mRNA [NM_006404] | -2.22 |
| FLJ35348 | Homo sapiens FLJ35348, mRNA (cDNA clone IMAGE:5398136), partial cds. [BC065204] | -2.22 |
| CA8 | Homo sapiens carbonic anhydrase VIII (CA8), mRNA [NM_004056] | -2.22 |
| SIN3B | Homo sapiens SIN3 homolog B, transcription regulator (yeast), mRNA (cDNA clone IMAGE:3923074), partial cds. [BC025026] | -2.21 |
| PRR7 | Homo sapiens proline rich 7 (synaptic) (PRR7), mRNA [NM_030567] | -2.21 |
| COMMD1 | Homo sapiens copper metabolism (Murr1) domain containing 1 (COMMD1), mRNA [NM_152516] | -2.21 |
| SERPINB9 | Homo sapiens serpin peptidase inhibitor, clade B (ovalbumin), member 9 (SERPINB9), mRNA [NM_004155] | -2.21 |
| MCEE | Homo sapiens methylmalonyl CoA epimerase (MCEE), mRNA [NM_032601] | -2.21 |
| PSMA1 | Homo sapiens proteasome (prosome, macropain) subunit, alpha type, 1 (PSMA1), transcript variant 1, mRNA [NM_148976] | -2.21 |
| DOK1 | Homo sapiens docking protein 1, 62kDa (downstream of tyrosine kinase 1) (DOK1), mRNA [NM_001381] | -2.21 |
| TM2D1 | Homo sapiens TM2 domain containing 1 (TM2D1), mRNA [NM_032027] | -2.21 |
| VPS24 | Homo sapiens vacuolar protein sorting 24 homolog (S. cerevisiae) (VPS24), transcript variant 1, mRNA [NM_016079] | -2.20 |
| SAMD4A | Homo sapiens sterile alpha motif domain containing 4A (SAMD4A), mRNA [NM_015589] | -2.20 |
| CR616939 | full-length cDNA clone CS0DF026YH11 of Fetal brain of Homo sapiens (human). [CR616939] | -2.20 |
| C10orf78 | Homo sapiens chromosome 10 open reading frame 78 (C10orf78), transcript variant 2, mRNA [NM_145247] | -2.20 |
| PEX16 | Homo sapiens peroxisomal biogenesis factor 16 (PEX16), transcript variant 1, mRNA [NM_004813] | -2.20 |
| LONRF2 | Homo sapiens LON peptidase N-terminal domain and ring finger 2 (LONRF2), mRNA [NM_198461] | -2.20 |
| SUSD3 | Homo sapiens sushi domain containing 3 (SUSD3), mRNA [NM_145006] | -2.20 |
| TMEM107 | Homo sapiens transmembrane protein 107 (TMEM107), transcript variant 1, mRNA [NM_032354] | -2.20 |
| CHMP2B | Homo sapiens chromatin modifying protein 2B (CHMP2B), mRNA [NM_014043] | -2.19 |
| GSTO1 | Homo sapiens glutathione S-transferase omega 1 (GSTO1), mRNA [NM_004832] | -2.19 |
| RPS27L | Homo sapiens ribosomal protein S27-like (RPS27L), mRNA [NM_015920] | -2.19 |
| FAM82B | Homo sapiens family with sequence similarity 82, member B (FAM82B), mRNA [NM_016033] | -2.19 |
| ENST00000377492 | FAM27E1 protein. [Source:Uniprot/SPTREMBL;Acc:Q08E93] [ENST00000377492] | -2.19 |
| FAM11A | Homo sapiens family with sequence similarity 11, member A (FAM11A), mRNA [NM_032508] | -2.19 |
| PAOX | Homo sapiens polyamine oxidase (exo-N4-amino) (PAOX), transcript variant 1, mRNA [NM_152911] | -2.18 |
| TIGD2 | Homo sapiens tigger transposable element derived 2 (TIGD2), mRNA [NM_145715] | -2.18 |
| BC084557 | Homo sapiens cDNA clone IMAGE:6302083. [BC084557] | -2.18 |
| CUL4B | Homo sapiens cullin 4B (CUL4B), mRNA [NM_003588] | -2.18 |
| ANKRD9 | Homo sapiens ankyrin repeat domain 9 (ANKRD9), mRNA [NM_152326] | -2.18 |
| ATP6V1C1 | Homo sapiens ATPase, H+ transporting, lysosomal 42kDa, V1 subunit C1 (ATP6V1C1), transcript variant 1, mRNA [NM_001695] | -2.18 |
| MKLN1 | Homo sapiens muskelin 1, intracellular mediator containing kelch motifs (MKLN1), mRNA [NM_013255] | -2.18 |
| PPM1J | Homo sapiens protein phosphatase 1J (PP2C domain containing) (PPM1J), mRNA [NM_005167] | -2.18 |
| PSIP1 | Homo sapiens PC4 and SFRS1 interacting protein 1 (PSIP1), transcript variant 2, mRNA [NM_033222] | -2.18 |
| SLC38A6 | Homo sapiens solute carrier family 38, member 6 (SLC38A6), mRNA [NM_153811] | -2.17 |
| RDH14 | Homo sapiens retinol dehydrogenase 14 (all-trans/9-cis/11-cis) (RDH14), mRNA [NM_020905] | -2.17 |
| ACYP1 | Homo sapiens acylphosphatase 1, erythrocyte (common) type (ACYP1), transcript variant 2, mRNA [NM_203488] | -2.17 |
| TBKBP1 | Homo sapiens TBK1 binding protein 1 (TBKBP1), mRNA [NM_014726] | -2.17 |
| NUDT8 | Homo sapiens nudix (nucleoside diphosphate linked moiety X)-type motif 8 (NUDT8), mRNA [NM_181843] | -2.17 |
| NR1H3 | Homo sapiens nuclear receptor subfamily 1, group H, member 3 (NR1H3), mRNA [NM_005693] | -2.17 |
| ABI2 | Homo sapiens abl interactor 2 (ABI2), mRNA [NM_005759] | -2.17 |
| RNASET2 | Homo sapiens ribonuclease T2 (RNASET2), mRNA [NM_003730] | -2.17 |
| ENST00000322831 | Zinc finger CCHC domain-containing protein 7. [Source:Uniprot/SWISSPROT;Acc:Q8N3Z6] [ENST00000322831] | -2.17 |
| PCID1 | Homo sapiens PCI domain containing 1 (herpesvirus entry mediator) (PCID1), mRNA [NM_006360] | -2.16 |
| AY090769 | Homo sapiens ribosomal protein S18/S6-like mRNA, complete sequence. [AY090769] | -2.16 |
| MGST3 | Homo sapiens microsomal glutathione S-transferase 3 (MGST3), mRNA [NM_004528] | -2.16 |
| GTPBP6 | Homo sapiens GTP binding protein 6 (putative) (GTPBP6), mRNA [NM_012227] | -2.16 |
| BC029473 | Homo sapiens cDNA clone IMAGE:4723680, **** WARNING: chimeric clone ****. [BC029473] | -2.16 |
| SUHW1 | Homo sapiens suppressor of hairy wing homolog 1 (Drosophila) (SUHW1), mRNA [NM_080740] | -2.16 |
| LOC731183 | PREDICTED: Homo sapiens similar to beta-1,4-mannosyltransferase (LOC731183), mRNA [XR_015406] | -2.16 |
| PRKAR1B | Homo sapiens protein kinase, cAMP-dependent, regulatory, type I, beta (PRKAR1B), mRNA [NM_002735] | -2.16 |
| WBP1 | Homo sapiens WW domain binding protein 1 (WBP1), mRNA [NM_012477] | -2.15 |
| KIAA0409 | Homo sapiens KIAA0409 (KIAA0409), mRNA [NM_015324] | -2.15 |
| THC2549673 | TM50A_HUMAN (O95807) Transmembrane protein 50A (Small membrane protein 1), partial (50%) [THC2549673] | -2.15 |
| PER3 | Homo sapiens period homolog 3 (Drosophila) (PER3), mRNA [NM_016831] | -2.15 |
| TDRD7 | Homo sapiens tudor domain containing 7 (TDRD7), mRNA [NM_014290] | -2.15 |
| COPG2 | Homo sapiens coatomer protein complex, subunit gamma 2 (COPG2), mRNA [NM_012133] | -2.15 |
| PIGK | Homo sapiens phosphatidylinositol glycan anchor biosynthesis, class K (PIGK), mRNA [NM_005482] | -2.15 |
| UNC84A | Homo sapiens unc-84 homolog A (C. elegans) (UNC84A), mRNA [NM_025154] | -2.14 |
| CRIPT | Homo sapiens cysteine-rich PDZ-binding protein (CRIPT), mRNA [NM_014171] | -2.14 |
| HIST1H2BH | Homo sapiens histone cluster 1, H2bh (HIST1H2BH), mRNA [NM_003524] | -2.14 |
| HIST1H2BF | Homo sapiens histone cluster 1, H2bf (HIST1H2BF), mRNA [NM_003522] | -2.14 |
| EFHC1 | Homo sapiens EF-hand domain (C-terminal) containing 1 (EFHC1), mRNA [NM_018100] | -2.14 |
| BRAF | Homo sapiens v-raf murine sarcoma viral oncogene homolog B1 (BRAF), mRNA [NM_004333] | -2.14 |
| CPAMD8 | Homo sapiens C3 and PZP-like, alpha-2-macroglobulin domain containing 8 (CPAMD8), mRNA [NM_015692] | -2.14 |
| SERPINB6 | Homo sapiens serpin peptidase inhibitor, clade B (ovalbumin), member 6 (SERPINB6), mRNA [NM_004568] | -2.13 |
| TMEM170 | Homo sapiens transmembrane protein 170 (TMEM170), mRNA [NM_145254] | -2.13 |
| COMMD6 | Homo sapiens COMM domain containing 6 (COMMD6), transcript variant 1, mRNA [NM_203497] | -2.13 |
| PLA2G4B | Homo sapiens phospholipase A2, group IVB (cytosolic), mRNA (cDNA clone IMAGE:4556397), complete cds. [BC025290] | -2.13 |
| SPR | Homo sapiens sepiapterin reductase (7,8-dihydrobiopterin:NADP+ oxidoreductase) (SPR), mRNA [NM_003124] | -2.13 |
| IRF7 | Homo sapiens interferon regulatory factor 7 (IRF7), transcript variant d, mRNA [NM_004031] | -2.13 |
| RAB8B | Homo sapiens RAB8B, member RAS oncogene family (RAB8B), mRNA [NM_016530] | -2.13 |
| TDRD10 | Homo sapiens tudor domain containing 10 (TDRD10), mRNA [NM_182499] | -2.13 |
| LOH12CR1 | Homo sapiens loss of heterozygosity, 12, chromosomal region 1 (LOH12CR1), mRNA [NM_058169] | -2.13 |
| ATP5H | Homo sapiens ATP synthase, H+ transporting, mitochondrial F0 complex, subunit d (ATP5H), nuclear gene encoding mitochondrial protein, transcript variant 1, mRNA [NM_006356] | -2.12 |
| WWOX | Homo sapiens WW domain containing oxidoreductase (WWOX), transcript variant 1, mRNA [NM_016373] | -2.12 |
| LOC442229 | Homo sapiens cDNA clone MGC:33544 IMAGE:4821649, complete cds. [BC024198] | -2.12 |
| A_24_P341616 | Unknown | -2.12 |
| BCL2L11 | Homo sapiens BCL2-like 11 (apoptosis facilitator) (BCL2L11), transcript variant 1, mRNA [NM_138621] | -2.12 |
| PCYOX1L | Homo sapiens prenylcysteine oxidase 1 like (PCYOX1L), mRNA [NM_024028] | -2.12 |
| HIST1H3B | Homo sapiens histone cluster 1, H3b (HIST1H3B), mRNA [NM_003537] | -2.12 |
| THC2688497 | Q59GX2_HUMAN (Q59GX2) Solute carrier family 2 (Facilitated glucose transporter), member 1 variant (Fragment), partial (6%) [THC2564899] | -2.12 |
| HIST1H2BE | Homo sapiens histone cluster 1, H2be (HIST1H2BE), mRNA [NM_003523] | -2.12 |
| COX17 | Homo sapiens COX17 cytochrome c oxidase assembly homolog (S. cerevisiae) (COX17), nuclear gene encoding mitochondrial protein, mRNA [NM_005694] | -2.11 |
| AMPD3 | Homo sapiens adenosine monophosphate deaminase (isoform E) (AMPD3), transcript variant 3, mRNA [NM_001025390] | -2.11 |
| LOH12CR1 | Homo sapiens loss of heterozygosity, 12, chromosomal region 1 (LOH12CR1), mRNA [NM_058169] | -2.11 |
| FAM92A1 | Homo sapiens mRNA; cDNA DKFZp564I0278 (from clone DKFZp564I0278). [CR627475] | -2.11 |
| C14orf169 | Homo sapiens chromosome 14 open reading frame 169 (C14orf169), mRNA [NM_024644] | -2.11 |
| C9orf119 | HBV DNAPTP1-transactivated protein A. [Source:Uniprot/SPTREMBL;Acc:Q1ZZU3] [ENST00000320188] | -2.11 |
| GABARAPL2 | Homo sapiens GABA(A) receptor-associated protein-like 2 (GABARAPL2), mRNA [NM_007285] | -2.11 |
| RRAS2 | Homo sapiens related RAS viral (r-ras) oncogene homolog 2 (RRAS2), mRNA [NM_012250] | -2.11 |
| HIST1H2BC | Homo sapiens histone cluster 1, H2bc (HIST1H2BC), mRNA [NM_003526] | -2.11 |
| HIST2H2BE | Homo sapiens histone cluster 2, H2be (HIST2H2BE), mRNA [NM_003528] | -2.10 |
| TBC1D20 | Homo sapiens TBC1 domain family, member 20, mRNA (cDNA clone MGC:23197 IMAGE:4861869), complete cds. [BC014983] | -2.10 |
| BU661610 | cl74d04.z1 Hembase; Erythroid Precursor Cells (LCB:cl library) Homo sapiens cDNA clone cl74d04 5', mRNA sequence [BU661610] | -2.10 |
| YWHAQ | Homo sapiens tyrosine 3-monooxygenase/tryptophan 5-monooxygenase activation protein, theta polypeptide (YWHAQ), mRNA [NM_006826] | -2.10 |
| EPB41L1 | Homo sapiens erythrocyte membrane protein band 4.1-like 1 (EPB41L1), transcript variant 1, mRNA [NM_012156] | -2.10 |
| AJ002788 | Homo sapiens mRNA for protein kinase C beta 1, 3' UTR; fetal brain cDNA ICRFp507K04156. [AJ002788] | -2.10 |
| API5 | Homo sapiens apoptosis inhibitor 5 (API5), mRNA [NM_006595] | -2.10 |
| C10orf4 | Homo sapiens chromosome 10 open reading frame 4 (C10orf4), transcript variant FRA10AC1-3.3, mRNA [NM_203438] | -2.10 |
| THC2668853 | Q416P5_KINRA (Q416P5) Serine--pyruvate transaminase , partial (3%) [THC2668853] | -2.10 |
| IMMP1L | Homo sapiens IMP1 inner mitochondrial membrane peptidase-like (S. cerevisiae) (IMMP1L), mRNA [NM_144981] | -2.10 |
| HERPUD1 | Homo sapiens homocysteine-inducible, endoplasmic reticulum stress-inducible, ubiquitin-like domain member 1 (HERPUD1), transcript variant 1, mRNA [NM_014685] | -2.10 |
| RAB11FIP4 | Homo sapiens RAB11 family interacting protein 4 (class II) (RAB11FIP4), mRNA [NM_032932] | -2.10 |
| ITCH | Homo sapiens itchy homolog E3 ubiquitin protein ligase (mouse) (ITCH), mRNA [NM_031483] | -2.10 |
| A_32_P101420 | Unknown | -2.10 |
| HEYL | Homo sapiens hairy/enhancer-of-split related with YRPW motif-like (HEYL), mRNA [NM_014571] | -2.09 |
| RNF141 | Homo sapiens ring finger protein 141 (RNF141), mRNA [NM_016422] | -2.09 |
| EIF2C3 | Homo sapiens eukaryotic translation initiation factor 2C, 3 (EIF2C3), transcript variant 1, mRNA [NM_024852] | -2.09 |
| ATP5S | Homo sapiens ATP synthase, H+ transporting, mitochondrial F0 complex, subunit s (factor B) (ATP5S), nuclear gene encoding mitochondrial protein, transcript variant 1, mRNA [NM_001003803] | -2.09 |
| MSL3L1 | Homo sapiens male-specific lethal 3-like 1 (Drosophila) (MSL3L1), transcript variant 1, mRNA [NM_078629] | -2.09 |
| C20orf112 | Homo sapiens cDNA FLJ40485 fis, clone TESTI2043857, moderately similar to Homo sapiens nolp mRNA. [AK097804] | -2.09 |
| TNFAIP1 | Homo sapiens tumor necrosis factor, alpha-induced protein 1 (endothelial) (TNFAIP1), mRNA [NM_021137] | -2.09 |
| DYNC1I2 | Homo sapiens dynein, cytoplasmic 1, intermediate chain 2 (DYNC1I2), mRNA [NM_001378] | -2.09 |
| FBXO3 | Homo sapiens F-box protein 3 (FBXO3), transcript variant 2, mRNA [NM_033406] | -2.09 |
| FCRLB | Homo sapiens Fc receptor-like B (FCRLB), mRNA [NM_001002901] | -2.08 |
| C14orf109 | Homo sapiens mRNA; cDNA DKFZp564F1123 (from clone DKFZp564F1123). [AL080118] | -2.08 |
| TMEM9B | Homo sapiens TMEM9 domain family, member B (TMEM9B), mRNA [NM_020644] | -2.08 |
| PEPD | Homo sapiens peptidase D (PEPD), mRNA [NM_000285] | -2.08 |
| LOC391566 | PREDICTED: Homo sapiens similar to Histone H2B 291B (LOC391566), mRNA [XR_018583] | -2.08 |
| GTDC1 | Homo sapiens glycosyltransferase-like domain containing 1 (GTDC1), transcript variant 1, mRNA [NM_001006636] | -2.08 |
| NAPRT1 | Homo sapiens nicotinate phosphoribosyltransferase domain containing 1 (NAPRT1), mRNA [NM_145201] | -2.08 |
| MTMR9 | Homo sapiens myotubularin related protein 9 (MTMR9), mRNA [NM_015458] | -2.08 |
| TMEM14A | Homo sapiens transmembrane protein 14A (TMEM14A), mRNA [NM_014051] | -2.08 |
| SERTAD1 | Homo sapiens SERTA domain containing 1 (SERTAD1), mRNA [NM_013376] | -2.08 |
| C7orf41 | Homo sapiens chromosome 7 open reading frame 41 (C7orf41), mRNA [NM_152793] | -2.08 |
| RNF170 | Homo sapiens ring finger protein 170 (RNF170), mRNA [NM_030954] | -2.08 |
| NUMB | Homo sapiens numb homolog (Drosophila) (NUMB), transcript variant 1, mRNA [NM_001005743] | -2.08 |
| PCID1 | Homo sapiens PCI domain containing 1 (herpesvirus entry mediator) (PCID1), mRNA [NM_006360] | -2.08 |
| ENST00000377492 | FAM27E1 protein. [Source:Uniprot/SPTREMBL;Acc:Q08E93] [ENST00000377492] | -2.08 |
| SLC7A8 | Homo sapiens solute carrier family 7 (cationic amino acid transporter, y+ system), member 8 (SLC7A8), transcript variant 2, mRNA [NM_182728] | -2.08 |
| TMEM54 | Homo sapiens transmembrane protein 54 (TMEM54), mRNA [NM_033504] | -2.07 |
| THC2618446 | Q213Y3_RHOPA (Q213Y3) Single-strand binding protein, partial (9%) [THC2618446] | -2.07 |
| TTC7A | Homo sapiens tetratricopeptide repeat domain 7A (TTC7A), mRNA [NM_020458] | -2.07 |
| FLJ32065 | Homo sapiens hypothetical protein FLJ32065, mRNA (cDNA clone MGC:90301 IMAGE:5502453), complete cds. [BC073870] | -2.07 |
| DHRS7 | Homo sapiens dehydrogenase/reductase (SDR family) member 7 (DHRS7), mRNA [NM_016029] | -2.07 |
| UBA52 | Homo sapiens ubiquitin A-52 residue ribosomal protein fusion product 1 (UBA52), transcript variant 1, mRNA [NM_001033930] | -2.07 |
| DYNC2LI1 | Homo sapiens dynein, cytoplasmic 2, light intermediate chain 1 (DYNC2LI1), transcript variant 2, mRNA [NM_015522] | -2.07 |
| CFLAR | Homo sapiens FLAME-1 mRNA, complete cds. [AF009616] | -2.07 |
| HSPC049 | Homo sapiens HSPC049 protein (HSPC049), mRNA [NM_014149] | -2.07 |
| N6AMT1 | Homo sapiens N-6 adenine-specific DNA methyltransferase 1 (putative) (N6AMT1), transcript variant 1, mRNA [NM_013240] | -2.07 |
| EIF2C2 | Homo sapiens eukaryotic translation initiation factor 2C, 2 (EIF2C2), mRNA [NM_012154] | -2.07 |
| A_32_P144629 | Unknown | -2.07 |
| COMMD4 | Homo sapiens COMM domain containing 4 (COMMD4), mRNA [NM_017828] | -2.07 |
| SLIT2 | Homo sapiens slit homolog 2 (Drosophila) (SLIT2), mRNA [NM_004787] | -2.07 |
| C11orf60 | Homo sapiens chromosome 11 open reading frame 60 (C11orf60), mRNA [NM_020153] | -2.07 |
| ZNF446 | Homo sapiens zinc finger protein 446 (ZNF446), mRNA [NM_017908] | -2.07 |
| HIST1H2BL | Homo sapiens histone cluster 1, H2bl (HIST1H2BL), mRNA [NM_003519] | -2.07 |
| FAM14B | Homo sapiens family with sequence similarity 14, member B (FAM14B), mRNA [NM_206949] | -2.07 |
| NUP62CL | Homo sapiens nucleoporin 62kDa C-terminal like (NUP62CL), mRNA [NM_017681] | -2.07 |
| C1orf182 | Homo sapiens chromosome 1 open reading frame 182 (C1orf182), mRNA [NM_144627] | -2.07 |
| ZNF277P | Homo sapiens zinc finger protein 277 pseudogene (ZNF277P), mRNA [NM_021994] | -2.07 |
| STK38L | Homo sapiens serine/threonine kinase 38 like (STK38L), mRNA [NM_015000] | -2.06 |
| A_32_P918263 | Unknown | -2.06 |
| AL390181 | Homo sapiens mRNA; cDNA DKFZp547J125 (from clone DKFZp547J125). [AL390181] | -2.06 |
| HIST1H2BG | Homo sapiens histone cluster 1, H2bg (HIST1H2BG), mRNA [NM_003518] | -2.06 |
| AKR7A2 | Homo sapiens aldo-keto reductase family 7, member A2 (aflatoxin aldehyde reductase) (AKR7A2), mRNA [NM_003689] | -2.06 |
| RPL23AP7 | Homo sapiens ribosomal protein L23a pseudogene 7 (RPL23AP7) on chromosome 2 [NR_000029] | -2.06 |
| MKRN1 | Homo sapiens znf-xp protein mRNA, complete cds. [AF117233] | -2.06 |
| DHRSX | Homo sapiens dehydrogenase/reductase (SDR family) X-linked (DHRSX), mRNA [NM_145177] | -2.06 |
| AFAR3 | Homo sapiens aflatoxin B1 aldehyde reductase 3 (AFAR3), mRNA [NM_201252] | -2.06 |
| ZDHHC24 | Homo sapiens zinc finger, DHHC-type containing 24, mRNA (cDNA clone IMAGE:3636082), partial cds. [BC005015] | -2.06 |
| FECH | Homo sapiens ferrochelatase (protoporphyria) (FECH), nuclear gene encoding mitochondrial protein, transcript variant 1, mRNA [NM_001012515] | -2.06 |
| LYPLAL1 | Homo sapiens lysophospholipase-like 1 (LYPLAL1), mRNA [NM_138794] | -2.06 |
| USP18 | Homo sapiens ubiquitin specific peptidase 18 (USP18), mRNA [NM_017414] | -2.06 |
| BCL2L11 | Homo sapiens BCL2-like 11 (apoptosis facilitator) (BCL2L11), transcript variant 1, mRNA [NM_138621] | -2.06 |
| HIST1H2BE | Homo sapiens histone cluster 1, H2be (HIST1H2BE), mRNA [NM_003523] | -2.05 |
| THC2668193 | Unknown | -2.05 |
| EHD2 | Homo sapiens EH-domain containing 2 (EHD2), mRNA [NM_014601] | -2.05 |
| KRCC1 | Homo sapiens lysine-rich coiled-coil 1 (KRCC1), mRNA [NM_016618] | -2.05 |
| TMED8 | Protein TMED8. [Source:Uniprot/SWISSPROT;Acc:Q6PL24] [ENST00000216468] | -2.05 |
| HIST1H2BJ | Homo sapiens histone cluster 1, H2bj (HIST1H2BJ), mRNA [NM_021058] | -2.05 |
| DZIP3 | Homo sapiens zinc finger DAZ interacting protein 3 (DZIP3), mRNA [NM_014648] | -2.05 |
| RMND5A | Homo sapiens required for meiotic nuclear division 5 homolog A (S. cerevisiae) (RMND5A), mRNA [NM_022780] | -2.05 |
| RFX2 | Homo sapiens regulatory factor X, 2 (influences HLA class II expression) (RFX2), transcript variant 1, mRNA [NM_000635] | -2.05 |
| DYNC2LI1 | Homo sapiens dynein, cytoplasmic 2, light intermediate chain 1 (DYNC2LI1), transcript variant 2, mRNA [NM_015522] | -2.05 |
| C11orf56 | Homo sapiens chromosome 11 open reading frame 56 (C11orf56), mRNA [NM_032127] | -2.05 |
| FAHD1 | Homo sapiens fumarylacetoacetate hydrolase domain containing 1 (FAHD1), transcript variant 2, mRNA [NM_031208] | -2.05 |
| RNF12 | Homo sapiens ring finger protein 12 (RNF12), transcript variant 1, mRNA [NM_016120] | -2.05 |
| GSTA4 | Homo sapiens glutathione S-transferase A4 (GSTA4), mRNA [NM_001512] | -2.05 |
| A_23_P17152 | Unknown | -2.05 |
| CHMP5 | Homo sapiens chromatin modifying protein 5 (CHMP5), mRNA [NM_016410] | -2.05 |
| IFT20 | Homo sapiens intraflagellar transport 20 homolog (Chlamydomonas) (IFT20), mRNA [NM_174887] | -2.04 |
| EBAG9 | Homo sapiens estrogen receptor binding site associated, antigen, 9 (EBAG9), transcript variant 1, mRNA [NM_004215] | -2.04 |
| APOM | Homo sapiens apolipoprotein M (APOM), mRNA [NM_019101] | -2.04 |
| HIST1H3F | Homo sapiens histone cluster 1, H3f (HIST1H3F), mRNA [NM_021018] | -2.04 |
| RAB9 | Homo sapiens RAB9, member RAS oncogene family (RAB9), mRNA [NM_004251] | -2.04 |
| SNX8 | Homo sapiens sorting nexin 8 (SNX8), mRNA [NM_013321] | -2.04 |
| RFK | Homo sapiens riboflavin kinase (RFK), mRNA [NM_018339] | -2.04 |
| HIST3H2BB | Homo sapiens histone cluster 3, H2bb (HIST3H2BB), mRNA [NM_175055] | -2.04 |
| A_24_P7330 | Unknown | -2.04 |
| GSTO1 | Homo sapiens glutathione S-transferase omega 1 (GSTO1), mRNA [NM_004832] | -2.04 |
| NSUN3 | Homo sapiens NOL1/NOP2/Sun domain family, member 3 (NSUN3), mRNA [NM_022072] | -2.04 |
| KLK1 | Homo sapiens kallikrein 1 (KLK1), mRNA [NM_002257] | -2.03 |
| ATP5S | Homo sapiens ATP synthase, H+ transporting, mitochondrial F0 complex, subunit s (factor B) (ATP5S), nuclear gene encoding mitochondrial protein, transcript variant 3, mRNA [NM_015684] | -2.03 |
| AK3L1 | Homo sapiens adenylate kinase 3-like 1 (AK3L1), nuclear gene encoding mitochondrial protein, transcript variant 5, mRNA [NM_001005353] | -2.03 |
| SIGIRR | Homo sapiens single immunoglobulin and toll-interleukin 1 receptor (TIR) domain (SIGIRR), mRNA [NM_021805] | -2.03 |
| THC2597502 | BC002110 Rpl24 protein {Mus musculus} (exp=-1; wgp=0; cg=0), partial (82%) [THC2597502] | -2.03 |
| C20orf108 | Homo sapiens chromosome 20 open reading frame 108 (C20orf108), mRNA [NM_080821] | -2.03 |
| SLC9A5 | Homo sapiens solute carrier family 9 (sodium/hydrogen exchanger), member 5 (SLC9A5), mRNA [NM_004594] | -2.03 |
| RAB6B | Homo sapiens RAB6B, member RAS oncogene family (RAB6B), mRNA [NM_016577] | -2.03 |
| CLIC2 | Homo sapiens chloride intracellular channel 2 (CLIC2), mRNA [NM_001289] | -2.03 |
| C16orf35 | Homo sapiens chromosome 16 open reading frame 35 (C16orf35), transcript variant 2, mRNA [NM_001039476] | -2.03 |
| BC014395 | Homo sapiens, clone IMAGE:3029191, mRNA. [BC014395] | -2.03 |
| CYB5D2 | Homo sapiens cytochrome b5 domain containing 2 (CYB5D2), mRNA [NM_144611] | -2.03 |
| HIST1H4F | Homo sapiens histone cluster 1, H4f (HIST1H4F), mRNA [NM_003540] | -2.02 |
| C11orf1 | Homo sapiens chromosome 11 open reading frame 1 (C11orf1), mRNA [NM_022761] | -2.02 |
| HIST1H2BN | Homo sapiens histone cluster 1, H2bn (HIST1H2BN), mRNA [NM_003520] | -2.02 |
| TOM1L1 | Homo sapiens target of myb1-like 1 (chicken) (TOM1L1), mRNA [NM_005486] | -2.02 |
| GSN | Homo sapiens gelsolin (amyloidosis, Finnish type) (GSN), transcript variant 2, mRNA [NM_198252] | -2.02 |
| AKR7A3 | Homo sapiens aldo-keto reductase family 7, member A3 (aflatoxin aldehyde reductase) (AKR7A3), mRNA [NM_012067] | -2.02 |
| RRAGC | Homo sapiens Ras-related GTP binding C (RRAGC), mRNA [NM_022157] | -2.02 |
| EPPB9 | Homo sapiens B9 protein (EPPB9), mRNA [NM_015681] | -2.02 |
| TATDN1 | Homo sapiens TatD DNase domain containing 1 (TATDN1), mRNA [NM_032026] | -2.02 |
| OGT | Homo sapiens O-linked N-acetylglucosamine (GlcNAc) transferase (UDP-N-acetylglucosamine:polypeptide-N-acetylglucosaminyl transferase) (OGT), transcript variant 1, mRNA [NM_181672] | -2.02 |
| APOBEC3G | Homo sapiens apolipoprotein B mRNA editing enzyme, catalytic polypeptide-like 3G (APOBEC3G), mRNA [NM_021822] | -2.02 |
| THC2538534 | Unknown | -2.02 |
| KIAA1729 | Homo sapiens KIAA1729 protein (KIAA1729), mRNA [NM_053042] | -2.02 |
| TMEM111 | Homo sapiens transmembrane protein 111 (TMEM111), mRNA [NM_018447] | -2.02 |
| CAMLG | Homo sapiens calcium modulating ligand (CAMLG), mRNA [NM_001745] | -2.02 |
| ENST00000331306 | PREDICTED: Homo sapiens similar to large subunit ribosomal protein L36a (LOC728202), mRNA [XM_001129191] | -2.02 |
| RIOK3 | Homo sapiens RIO kinase 3 (yeast) (RIOK3), transcript variant 2, mRNA [NM_145906] | -2.02 |
| YTHDC2 | Homo sapiens YTH domain containing 2 (YTHDC2), mRNA [NM_022828] | -2.01 |
| UCHL5 | Homo sapiens ubiquitin carboxyl-terminal hydrolase L5 (UCHL5), mRNA [NM_015984] | -2.01 |
| ACSM3 | Homo sapiens acyl-CoA synthetase medium-chain family member 3 (ACSM3), transcript variant 2, mRNA [NM_202000] | -2.01 |
| A_32_P3914 | Unknown | -2.01 |
| PCMTD1 | Homo sapiens protein-L-isoaspartate (D-aspartate) O-methyltransferase domain containing 1 (PCMTD1), mRNA [NM_052937] | -2.01 |
| AY358619 | Homo sapiens clone DNA107443 AGLW2560 (UNQ2560) mRNA, complete cds. [AY358619] | -2.01 |
| RNASEH1 | Homo sapiens ribonuclease H1 (RNASEH1), mRNA [NM_002936] | -2.01 |
| HIST1H4H | Homo sapiens histone cluster 1, H4h (HIST1H4H), mRNA [NM_003543] | -2.01 |
| GTF3C4 | Homo sapiens general transcription factor IIIC, polypeptide 4, 90kDa (GTF3C4), mRNA [NM_012204] | -2.00 |
| GABARAPL2 | Homo sapiens GABA(A) receptor-associated protein-like 2 (GABARAPL2), mRNA [NM_007285] | -2.00 |
| MKRN3 | Homo sapiens makorin, ring finger protein, 3 (MKRN3), mRNA [NM_005664] | -2.00 |
| LOC729768 | PREDICTED: Homo sapiens similar to signal sequence receptor gamma subunit (LOC729768), mRNA [XR_016019] | -2.00 |
| DKFZp434N035 | Homo sapiens hypothetical protein DKFZp434N035 (DKFZp434N035), mRNA [NM_032262] | -2.00 |
| C14orf156 | Homo sapiens chromosome 14 open reading frame 156 (C14orf156), mRNA [NM_031210] | -2.00 |
| BOLA3 | Homo sapiens bolA homolog 3 (E. coli) (BOLA3), transcript variant 1, mRNA [NM_212552] | -2.00 |
| BQ438133 | AGENCOURT_7858573 NIH_MGC_71 Homo sapiens cDNA clone IMAGE:6175090 5', mRNA sequence [BQ438133] | -2.00 |
| C5orf32 | Homo sapiens chromosome 5 open reading frame 32 (C5orf32), mRNA [NM_032412] | -2.00 |
| OXNAD1 | Homo sapiens oxidoreductase NAD-binding domain containing 1 (OXNAD1), mRNA [NM_138381] | 2.00 |
| ENST00000355793 | Prothymosin alpha. [Source:Uniprot/SPTREMBL;Acc:Q15203] [ENST00000355793] | 2.00 |
| RPLP0 | Homo sapiens ribosomal protein, large, P0 (RPLP0), transcript variant 2, mRNA [NM_053275] | 2.00 |
| AK021744 | Homo sapiens cDNA FLJ11682 fis, clone HEMBA1004880. [AK021744] | 2.00 |
| C12orf52 | Homo sapiens chromosome 12 open reading frame 52 (C12orf52), mRNA [NM_032848] | 2.00 |
| TULP4 | Homo sapiens tubby like protein 4 (TULP4), transcript variant 1, mRNA [NM_020245] | 2.00 |
| TCHP | Homo sapiens trichoplein, keratin filament binding (TCHP), mRNA [NM_032300] | 2.00 |
| LYL1 | Homo sapiens lymphoblastic leukemia derived sequence 1 (LYL1), mRNA [NM_005583] | 2.00 |
| ZZZ3 | Homo sapiens zinc finger, ZZ-type containing 3 (ZZZ3), mRNA [NM_015534] | 2.00 |
| RIMS3 | Homo sapiens regulating synaptic membrane exocytosis 3 (RIMS3), mRNA [NM_014747] | 2.00 |
| LOC643988 | Homo sapiens mRNA, clone: TH072F08. [AB188495] | 2.00 |
| MPHOSPH9 | Homo sapiens M-phase phosphoprotein 9 (MPHOSPH9), mRNA [NM_022782] | 2.00 |
| CHPT1 | Homo sapiens choline phosphotransferase 1 (CHPT1), mRNA [NM_020244] | 2.00 |
| METTL7A | Homo sapiens methyltransferase like 7A (METTL7A), mRNA [NM_014033] | 2.00 |
| NT5C2 | Homo sapiens 5'-nucleotidase, cytosolic II (NT5C2), mRNA [NM_012229] | 2.00 |
| TMED2 | Homo sapiens transmembrane emp24 domain trafficking protein 2 (TMED2), mRNA [NM_006815] | 2.01 |
| GOPC | Homo sapiens golgi associated PDZ and coiled-coil motif containing (GOPC), transcript variant 1, mRNA [NM_020399] | 2.01 |
| CMTM6 | Homo sapiens CKLF-like MARVEL transmembrane domain containing 6 (CMTM6), mRNA [NM_017801] | 2.01 |
| SUMF2 | Homo sapiens sulfatase modifying factor 2 (SUMF2), transcript variant 1, mRNA [NM_001042468] | 2.01 |
| NARG1 | Homo sapiens NMDA receptor regulated 1 (NARG1), mRNA [NM_057175] | 2.01 |
| ARHGAP22 | Homo sapiens Rho GTPase activating protein 22 (ARHGAP22), mRNA [NM_021226] | 2.01 |
| VPS37B | Homo sapiens vacuolar protein sorting 37 homolog B (S. cerevisiae) (VPS37B), mRNA [NM_024667] | 2.01 |
| SEC63 | Homo sapiens SEC63 homolog (S. cerevisiae) (SEC63), mRNA [NM_007214] | 2.01 |
| PPIA | Homo sapiens peptidylprolyl isomerase A (cyclophilin A) (PPIA), mRNA [NM_021130] | 2.01 |
| CXCL3 | Homo sapiens chemokine (C-X-C motif) ligand 3 (CXCL3), mRNA [NM_002090] | 2.01 |
| AK129879 | Homo sapiens cDNA FLJ26369 fis, clone HRT06001. [AK129879] | 2.01 |
| LOC390299 | Unknown | 2.01 |
| METTL1 | Homo sapiens methyltransferase like 1 (METTL1), transcript variant 1, mRNA [NM_005371] | 2.01 |
| DLG1 | Homo sapiens mRNA; cDNA DKFZp761P0818 (from clone DKFZp761P0818). [AL831922] | 2.01 |
| MYNN | Homo sapiens myoneurin (MYNN), mRNA [NM_018657] | 2.01 |
| PUS1 | Homo sapiens pseudouridylate synthase 1 (PUS1), transcript variant 1, mRNA [NM_025215] | 2.01 |
| SP100 | Homo sapiens SP100 nuclear antigen (SP100), mRNA [NM_003113] | 2.01 |
| LOC644016 | PREDICTED: Homo sapiens similar to heat shock protein 1 (chaperonin) (LOC644016), mRNA [XR_018885] | 2.02 |
| WSB2 | Homo sapiens WD repeat and SOCS box-containing 2 (WSB2), mRNA [NM_018639] | 2.02 |
| HNRPC | Homo sapiens heterogeneous nuclear ribonucleoprotein C (C1/C2) (HNRPC), transcript variant 1, mRNA [NM_031314] | 2.02 |
| PRPF4B | Homo sapiens PRP4 pre-mRNA processing factor 4 homolog B (yeast) (PRPF4B), mRNA [NM_003913] | 2.02 |
| BX648950 | Homo sapiens mRNA; cDNA DKFZp686E1648 (from clone DKFZp686E1648). [BX648950] | 2.02 |
| SGPL1 | Homo sapiens sphingosine-1-phosphate lyase 1 (SGPL1), mRNA [NM_003901] | 2.02 |
| NCSTN | Homo sapiens nicastrin (NCSTN), mRNA [NM_015331] | 2.02 |
| BU507302 | AGENCOURT_10309688 NIH_MGC_71 Homo sapiens cDNA clone IMAGE:6501220 5', mRNA sequence [BU507302] | 2.02 |
| PEA15 | Homo sapiens phosphoprotein enriched in astrocytes 15 (PEA15), mRNA [NM_003768] | 2.02 |
| A_24_P186779 | Unknown | 2.02 |
| CHD1 | Homo sapiens chromodomain helicase DNA binding protein 1 (CHD1), mRNA [NM_001270] | 2.02 |
| THC2747796 | Unknown | 2.02 |
| TRIP6 | Homo sapiens thyroid hormone receptor interactor 6 (TRIP6), mRNA [NM_003302] | 2.02 |
| THC2682884 | Unknown | 2.02 |
| RPLP0 | Homo sapiens ribosomal protein, large, P0 (RPLP0), transcript variant 2, mRNA [NM_053275] | 2.02 |
| FAM3C | Homo sapiens family with sequence similarity 3, member C (FAM3C), transcript variant 1, mRNA [NM_014888] | 2.02 |
| NEK1 | Homo sapiens NIMA (never in mitosis gene a)-related kinase 1 (NEK1), mRNA [NM_012224] | 2.03 |
| CENPJ | Homo sapiens centromere protein J (CENPJ), mRNA [NM_018451] | 2.03 |
| SLC16A1 | Homo sapiens solute carrier family 16, member 1 (monocarboxylic acid transporter 1) (SLC16A1), mRNA [NM_003051] | 2.03 |
| LRRFIP1 | Homo sapiens leucine rich repeat (in FLII) interacting protein 1 (LRRFIP1), mRNA [NM_004735] | 2.03 |
| IDE | Insulin-degrading enzyme (EC 3.4.24.56) (Insulysin) (Insulinase) (Insulin protease). [Source:Uniprot/SWISSPROT;Acc:P14735] [ENST00000265986] | 2.03 |
| ECT2 | Homo sapiens epithelial cell transforming sequence 2 oncogene (ECT2), mRNA [NM_018098] | 2.03 |
| PPIA | Homo sapiens peptidylprolyl isomerase A (cyclophilin A) (PPIA), mRNA [NM_021130] | 2.03 |
| FAM116A | Homo sapiens family with sequence similarity 116, member A (FAM116A), mRNA [NM_152678] | 2.03 |
| DDX21 | Homo sapiens DEAD (Asp-Glu-Ala-Asp) box polypeptide 21 (DDX21), mRNA [NM_004728] | 2.03 |
| TLE4 | Homo sapiens transducin-like enhancer of split 4 (E(sp1) homolog, Drosophila) (TLE4), mRNA [NM_007005] | 2.03 |
| DBF4 | Homo sapiens DBF4 homolog (S. cerevisiae) (DBF4), mRNA [NM_006716] | 2.03 |
| RFC4 | Homo sapiens replication factor C (activator 1) 4, 37kDa (RFC4), transcript variant 1, mRNA [NM_002916] | 2.03 |
| NUDT15 | Homo sapiens nudix (nucleoside diphosphate linked moiety X)-type motif 15 (NUDT15), mRNA [NM_018283] | 2.03 |
| LYSMD3 | Homo sapiens LysM, putative peptidoglycan-binding, domain containing 3 (LYSMD3), mRNA [NM_198273] | 2.04 |
| KIAA0692 | Homo sapiens cDNA FLJ13999 fis, clone Y79AA1002234, highly similar to Homo sapiens mRNA for KIAA0692 protein. [AK024061] | 2.04 |
| TMED2 | Homo sapiens transmembrane emp24 domain trafficking protein 2 (TMED2), mRNA [NM_006815] | 2.04 |
| CSK | Homo sapiens c-src tyrosine kinase (CSK), mRNA [NM_004383] | 2.04 |
| CAST | Homo sapiens calpastatin (CAST), transcript variant 1, mRNA [NM_001750] | 2.04 |
| NEDD4L | Homo sapiens neural precursor cell expressed, developmentally down-regulated 4-like (NEDD4L), mRNA [NM_015277] | 2.04 |
| HNRPDL | Homo sapiens heterogeneous nuclear ribonucleoprotein D-like (HNRPDL), transcript variant 2, mRNA [NM_031372] | 2.04 |
| Sep-02 | Homo sapiens septin 2 (SEPT2), transcript variant 1, mRNA [NM_001008491] | 2.04 |
| ACSS2 | Homo sapiens acyl-CoA synthetase short-chain family member 2 (ACSS2), transcript variant 1, mRNA [NM_018677] | 2.04 |
| BC039246 | Homo sapiens cDNA clone IMAGE:5278517. [BC039246] | 2.04 |
| THC2622086 | HSA277441 xylosyltransferase I {Homo sapiens} (exp=-1; wgp=0; cg=0), partial (8%) [THC2622086] | 2.04 |
| PASK | Homo sapiens PAS domain containing serine/threonine kinase (PASK), mRNA [NM_015148] | 2.04 |
| COTL1 | Homo sapiens coactosin-like 1 (Dictyostelium) (COTL1), mRNA [NM_021149] | 2.04 |
| PTEN | Homo sapiens phosphatase and tensin homolog (mutated in multiple advanced cancers 1) (PTEN), mRNA [NM_000314] | 2.04 |
| CR622586 | full-length cDNA clone CS0DI026YN16 of Placenta Cot 25-normalized of Homo sapiens (human). [CR622586] | 2.05 |
| BX107836 | BX107836 Soares_fetal_heart_NbHH19W Homo sapiens cDNA clone IMAGp998I17743, mRNA sequence [BX107836] | 2.05 |
| ING5 | Homo sapiens inhibitor of growth family, member 5 (ING5), mRNA [NM_032329] | 2.05 |
| A_24_P840868 | Unknown | 2.05 |
| Sep-05 | Homo sapiens septin 5 (SEPT5), mRNA [NM_002688] | 2.05 |
| HACE1 | Homo sapiens HECT domain and ankyrin repeat containing, E3 ubiquitin protein ligase 1 (HACE1), mRNA [NM_020771] | 2.05 |
| RANBP5 | Homo sapiens RAN binding protein 5 (RANBP5), mRNA [NM_002271] | 2.05 |
| TLOC1 | Homo sapiens translocation protein 1 (TLOC1), mRNA [NM_003262] | 2.05 |
| LOC441212 | Homo sapiens PNAS-13 mRNA, complete cds. [AF274938] | 2.05 |
| PPP4R2 | Homo sapiens protein phosphatase 4, regulatory subunit 2 (PPP4R2), mRNA [NM_174907] | 2.05 |
| KIAA1450 | Homo sapiens mRNA for KIAA1450 protein, partial cds. [AB040883] | 2.05 |
| FLJ38663 | Homo sapiens hypothetical protein FLJ38663 (FLJ38663), mRNA [NM_152269] | 2.05 |
| DLG1 | Homo sapiens discs, large homolog 1 (Drosophila) (DLG1), mRNA [NM_004087] | 2.05 |
| MAP4 | Homo sapiens microtubule-associated protein 4 (MAP4), transcript variant 1, mRNA [NM_002375] | 2.06 |
| MAPK6 | Homo sapiens mitogen-activated protein kinase 6 (MAPK6), mRNA [NM_002748] | 2.06 |
| CD99L2 | Homo sapiens CD99 molecule-like 2 (CD99L2), transcript variant 1, mRNA [NM_031462] | 2.06 |
| TRAK1 | Homo sapiens trafficking protein, kinesin binding 1 (TRAK1), transcript variant 1, mRNA [NM_001042646] | 2.06 |
| CTGLF4 | Homo sapiens centaurin, gamma-like family, member 4 (CTGLF4), mRNA [NM_001077685] | 2.06 |
| C6orf60 | Homo sapiens chromosome 6 open reading frame 60 (C6orf60), mRNA [NM_024581] | 2.06 |
| RASIP1 | Homo sapiens Ras interacting protein 1 (RASIP1), mRNA [NM_017805] | 2.06 |
| PEBP1 | Homo sapiens phosphatidylethanolamine binding protein 1 (PEBP1), mRNA [NM_002567] | 2.06 |
| SURF6 | Homo sapiens surfeit 6 (SURF6), mRNA [NM_006753] | 2.06 |
| CALML4 | Homo sapiens calmodulin-like 4 (CALML4), transcript variant 1, mRNA [NM_033429] | 2.06 |
| SURF6 | Homo sapiens surfeit 6 (SURF6), mRNA [NM_006753] | 2.06 |
| PPIA | Homo sapiens peptidylprolyl isomerase A (cyclophilin A) (PPIA), mRNA [NM_021130] | 2.06 |
| HMGN2 | Homo sapiens high-mobility group nucleosomal binding domain 2 (HMGN2), mRNA [NM_005517] | 2.06 |
| AK022936 | Homo sapiens cDNA FLJ12874 fis, clone NT2RP2003769. [AK022936] | 2.06 |
| PALM | Homo sapiens paralemmin (PALM), transcript variant 1, mRNA [NM_002579] | 2.06 |
| KIAA0664 | Homo sapiens KIAA0664 (KIAA0664), mRNA [NM_015229] | 2.06 |
| AK098422 | Homo sapiens cDNA FLJ25556 fis, clone JTH02629. [AK098422] | 2.06 |
| TMPO | Homo sapiens thymopoietin (TMPO), transcript variant 1, mRNA [NM_003276] | 2.06 |
| ZNF174 | Homo sapiens zinc finger protein 174 (ZNF174), transcript variant 1, mRNA [NM_003450] | 2.06 |
| SSR3 | Homo sapiens signal sequence receptor, gamma (translocon-associated protein gamma) (SSR3), mRNA [NM_007107] | 2.07 |
| AK094623 | Homo sapiens cDNA FLJ37304 fis, clone BRAMY2016070. [AK094623] | 2.07 |
| TCFL5 | Homo sapiens cDNA clone IMAGE:5787400, partial cds. [BC065520] | 2.07 |
| AHCTF1 | Homo sapiens AT hook containing transcription factor 1 (AHCTF1), mRNA [NM_015446] | 2.07 |
| STMN3 | Homo sapiens stathmin-like 3 (STMN3), mRNA [NM_015894] | 2.07 |
| ALS2CR2 | Homo sapiens amyotrophic lateral sclerosis 2 (juvenile) chromosome region, candidate 2 (ALS2CR2), mRNA [NM_018571] | 2.07 |
| AK026647 | Homo sapiens cDNA: FLJ22994 fis, clone KAT11918. [AK026647] | 2.07 |
| LIN28B | Homo sapiens lin-28 homolog B (C. elegans) (LIN28B), mRNA [NM_001004317] | 2.07 |
| RFC3 | Homo sapiens replication factor C (activator 1) 3, 38kDa (RFC3), transcript variant 2, mRNA [NM_181558] | 2.07 |
| AK024870 | Homo sapiens cDNA: FLJ21217 fis, clone COL00536. [AK024870] | 2.07 |
| AP2M1 | Homo sapiens adaptor-related protein complex 2, mu 1 subunit (AP2M1), transcript variant 1, mRNA [NM_004068] | 2.07 |
| NOLC1 | Homo sapiens nucleolar and coiled-body phosphoprotein 1, mRNA (cDNA clone MGC:5049 IMAGE:2900024), complete cds. [BC006769] | 2.07 |
| BCLAF1 | Homo sapiens BCL2-associated transcription factor 1 (BCLAF1), transcript variant 1, mRNA [NM_014739] | 2.07 |
| DCUN1D2 | Homo sapiens DCN1, defective in cullin neddylation 1, domain containing 2 (S. cerevisiae) (DCUN1D2), mRNA [NM_001014283] | 2.07 |
| KIAA0649 | Homo sapiens KIAA0649 (KIAA0649), mRNA [NM_014811] | 2.07 |
| ENST00000355049 | similar to peptidylprolyl isomerase A isoform 1 (LOC126170), mRNA [Source:RefSeq_dna;Acc:XR_016157] [ENST00000355049] | 2.08 |
| GIT2 | Homo sapiens G protein-coupled receptor kinase interactor 2 (GIT2), transcript variant 4, mRNA [NM_139201] | 2.08 |
| UPF3A | Homo sapiens UPF3 regulator of nonsense transcripts homolog A (yeast) (UPF3A), transcript variant 1, mRNA [NM_023011] | 2.08 |
| PILRB | Homo sapiens paired immunoglobin-like type 2 receptor beta (PILRB), transcript variant 1, mRNA [NM_013440] | 2.08 |
| BCL7A | Homo sapiens B-cell CLL/lymphoma 7A (BCL7A), transcript variant 1, mRNA [NM_020993] | 2.08 |
| MAML1 | Homo sapiens mastermind-like 1 (Drosophila) (MAML1), mRNA [NM_014757] | 2.08 |
| BC013799 | Homo sapiens, clone IMAGE:4386363, mRNA. [BC013799] | 2.08 |
| GTPBP5 | Homo sapiens GTP binding protein 5 (putative) (GTPBP5), mRNA [NM_015666] | 2.08 |
| NEK1 | Homo sapiens NIMA (never in mitosis gene a)-related kinase 1 (NEK1), mRNA [NM_012224] | 2.08 |
| LOC341457 | PREDICTED: Homo sapiens similar to peptidylprolyl isomerase A isoform 1 (LOC341457), mRNA [XM_292085] | 2.08 |
| GOLGA4 | Homo sapiens golgi autoantigen, golgin subfamily a, 4 (GOLGA4), mRNA [NM_002078] | 2.08 |
| POGZ | Homo sapiens pogo transposable element with ZNF domain (POGZ), transcript variant 1, mRNA [NM_015100] | 2.09 |
| FBXO28 | F-box only protein 28. [Source:Uniprot/SWISSPROT;Acc:Q9NVF7] [ENST00000366862] | 2.09 |
| SSRP1 | Homo sapiens structure specific recognition protein 1 (SSRP1), mRNA [NM_003146] | 2.09 |
| AK129838 | Homo sapiens cDNA FLJ26328 fis, clone HRT01493. [AK129838] | 2.09 |
| THC2554861 | AV739766 AV739766 CB Homo sapiens cDNA clone CBDAGB02 5', mRNA sequence [AV739766] | 2.09 |
| RNF169 | RING finger protein 169. [Source:Uniprot/SWISSPROT;Acc:Q8NCN4] [ENST00000299563] | 2.09 |
| R3HDM2 | Homo sapiens R3H domain containing 2 (R3HDM2), mRNA [NM_014925] | 2.09 |
| CCDC86 | Homo sapiens coiled-coil domain containing 86 (CCDC86), mRNA [NM_024098] | 2.09 |
| DLG1 | Homo sapiens mRNA; cDNA DKFZp761P0818 (from clone DKFZp761P0818). [AL831922] | 2.09 |
| UNC13D | Homo sapiens unc-13 homolog D (C. elegans) (UNC13D), mRNA [NM_199242] | 2.09 |
| PHIP | Homo sapiens pleckstrin homology domain interacting protein (PHIP), mRNA [NM_017934] | 2.09 |
| NCOR2 | Homo sapiens nuclear receptor co-repressor 2 (NCOR2), transcript variant 1, mRNA [NM_006312] | 2.09 |
| LOC440353 | Homo sapiens nuclear pore complex interacting protein pseudogene (LOC440353) on chromosome 16 [NR_002603] | 2.09 |
| SELPLG | Homo sapiens selectin P ligand (SELPLG), mRNA [NM_003006] | 2.10 |
| ZFYVE28 | Homo sapiens zinc finger, FYVE domain containing 28 (ZFYVE28), mRNA [NM_020972] | 2.10 |
| E2F5 | Homo sapiens E2F transcription factor 5, p130-binding (E2F5), mRNA [NM_001951] | 2.10 |
| NR2C1 | Homo sapiens nuclear receptor subfamily 2, group C, member 1 (NR2C1), transcript variant 1, mRNA [NM_003297] | 2.10 |
| WDFY1 | Homo sapiens WD repeat and FYVE domain containing 1 (WDFY1), mRNA [NM_020830] | 2.10 |
| UBE2N | Homo sapiens ubiquitin-conjugating enzyme E2N (UBC13 homolog, yeast) (UBE2N), mRNA [NM_003348] | 2.10 |
| SART3 | Homo sapiens squamous cell carcinoma antigen recognized by T cells 3 (SART3), mRNA [NM_014706] | 2.10 |
| LOC442019 | PREDICTED: Homo sapiens similar to peptidylprolyl isomerase A isoform 1 (LOC442019), mRNA [XR_019337] | 2.10 |
| LOC442582 | Homo sapiens STAG3-like (LOC442582), mRNA [NM_001025202] | 2.10 |
| NPAL3 | Homo sapiens NIPA-like domain containing 3 (NPAL3), mRNA [NM_020448] | 2.10 |
| KIAA0907 | Homo sapiens KIAA0907 (KIAA0907), mRNA [NM_014949] | 2.10 |
| ZDHHC14 | Homo sapiens zinc finger, DHHC-type containing 14 (ZDHHC14), transcript variant 2, mRNA [NM_153746] | 2.10 |
| AK130366 | Homo sapiens cDNA FLJ26856 fis, clone PRS08123. [AK130366] | 2.10 |
| RAN | Homo sapiens RAN, member RAS oncogene family (RAN), mRNA [NM_006325] | 2.10 |
| EIF1AX | Homo sapiens eukaryotic translation initiation factor 1A, X-linked (EIF1AX), mRNA [NM_001412] | 2.10 |
| BRD9 | Homo sapiens bromodomain containing 9 (BRD9), transcript variant 1, mRNA [NM_023924] | 2.10 |
| ELK4 | ETS domain-containing protein Elk-4 (Serum response factor accessory protein 1) (SAP-1). [Source:Uniprot/SWISSPROT;Acc:P28324] [ENST00000367146] | 2.11 |
| MAN2A1 | Homo sapiens mannosidase, alpha, class 2A, member 1 (MAN2A1), mRNA [NM_002372] | 2.11 |
| LNPEP | Leucyl-cystinyl aminopeptidase (EC 3.4.11.3) (Cystinyl aminopeptidase) (Oxytocinase) (OTase) (Insulin-regulated membrane aminopeptidase) (Insulin-responsive aminopeptidase) (IRAP) (Placental leucine aminopeptidase) (P-LAP).... | 2.11 |
| NO145 | Homo sapiens 145 kDa nucleolar protein (NO145), mRNA [NM_001040274] | 2.11 |
| THC2703681 | Q9BVD9_HUMAN (Q9BVD9) FTO protein, partial (16%) [THC2703681] | 2.11 |
| C1orf38 | Homo sapiens chromosome 1 open reading frame 38 (C1orf38), transcript variant 2, mRNA [NM_001039477] | 2.11 |
| MUTED | Homo sapiens muted homolog (mouse) (MUTED), mRNA [NM_201280] | 2.11 |
| HCST | Homo sapiens hematopoietic cell signal transducer (HCST), transcript variant 1, mRNA [NM_014266] | 2.11 |
| RYBP | Homo sapiens RING1 and YY1 binding protein (RYBP), mRNA [NM_012234] | 2.11 |
| GKAP1 | Homo sapiens G kinase anchoring protein 1 (GKAP1), mRNA [NM_025211] | 2.11 |
| IDH1 | Homo sapiens isocitrate dehydrogenase 1 (NADP+), soluble (IDH1), mRNA [NM_005896] | 2.11 |
| RHOBTB1 | Homo sapiens Rho-related BTB domain containing 1 (RHOBTB1), transcript variant 1, mRNA [NM_014836] | 2.11 |
| SESN1 | Homo sapiens sestrin 1 (SESN1), mRNA [NM_014454] | 2.11 |
| THC2676284 | Q6DN14_HUMAN (Q6DN14) MCTP1L, partial (4%) [THC2676284] | 2.11 |
| TPM3 | Homo sapiens tropomyosin 3 (TPM3), transcript variant 2, mRNA [NM_153649] | 2.11 |
| PRDM4 | Homo sapiens PR domain containing 4 (PRDM4), mRNA [NM_012406] | 2.11 |
| TPM3 | Homo sapiens tropomyosin 3 (TPM3), transcript variant 1, mRNA [NM_152263] | 2.12 |
| CNN1 | Homo sapiens calponin 1, basic, smooth muscle (CNN1), mRNA [NM_001299] | 2.12 |
| CAB39 | Homo sapiens calcium binding protein 39 (CAB39), mRNA [NM_016289] | 2.12 |
| C20orf11 | Homo sapiens chromosome 20 open reading frame 11 (C20orf11), mRNA [NM_017896] | 2.12 |
| SHPRH | Homo sapiens SNF2 histone linker PHD RING helicase (SHPRH), transcript variant 1, mRNA [NM_001042683] | 2.12 |
| NOS3 | Homo sapiens nitric oxide synthase 3 (endothelial cell) (NOS3), mRNA [NM_000603] | 2.12 |
| PLEKHA5 | Homo sapiens pleckstrin homology domain containing, family A member 5 (PLEKHA5), mRNA [NM_019012] | 2.13 |
| ENST00000378679 | apical protein 2 [Source:RefSeq_peptide;Acc:NP_597713] [ENST00000378679] | 2.13 |
| CHDH | Homo sapiens cDNA FLJ30840 fis, clone FEBRA2002442, highly similar to Homo sapiens partial mRNA for choline dehydrogenase. [AK055402] | 2.13 |
| PAK2 | Homo sapiens p21 (CDKN1A)-activated kinase 2 (PAK2), mRNA [NM_002577] | 2.13 |
| REV3L | Homo sapiens REV3-like, catalytic subunit of DNA polymerase zeta (yeast) (REV3L), mRNA [NM_002912] | 2.13 |
| RPL35A | Homo sapiens ribosomal protein L35a (RPL35A), mRNA [NM_000996] | 2.13 |
| RPL22L1 | Homo sapiens ribosomal protein L22-like 1, mRNA (cDNA clone IMAGE:4865966). [BC049823] | 2.13 |
| CMTM6 | Homo sapiens CKLF-like MARVEL transmembrane domain containing 6 (CMTM6), mRNA [NM_017801] | 2.13 |
| PRKCBP1 | Homo sapiens mRNA; cDNA DKFZp564P1772 (from clone DKFZp564P1772). [AL137703] | 2.14 |
| AF161353 | Homo sapiens HSPC090 mRNA, partial cds. [AF161353] | 2.14 |
| CUL3 | Homo sapiens cullin 3 (CUL3), mRNA [NM_003590] | 2.14 |
| THC2540396 | ALU8_HUMAN (P39195) Alu subfamily SX sequence contamination warning entry, partial (23%) [THC2540396] | 2.14 |
| COQ10B | Homo sapiens coenzyme Q10 homolog B (S. cerevisiae) (COQ10B), mRNA [NM_025147] | 2.14 |
| SEC63 | Homo sapiens SEC63 homolog (S. cerevisiae) (SEC63), mRNA [NM_007214] | 2.14 |
| TMEM181 | Homo sapiens mRNA for KIAA1423 protein, partial cds. [AB037844] | 2.14 |
| KIAA1856 | Homo sapiens cDNA FLJ14435 fis, clone HEMBA1007085. [AK027341] | 2.15 |
| ZBTB2 | Homo sapiens zinc finger and BTB domain containing 2 (ZBTB2), mRNA [NM_020861] | 2.15 |
| WDFY2 | Homo sapiens WD repeat and FYVE domain containing 2 (WDFY2), mRNA [NM_052950] | 2.15 |
| RANBP1 | Homo sapiens RAN binding protein 1 (RANBP1), mRNA [NM_002882] | 2.15 |
| FBXL10 | Homo sapiens F-box and leucine-rich repeat protein 10 (FBXL10), transcript variant 1, mRNA [NM_032590] | 2.15 |
| LOC730850 | PREDICTED: Homo sapiens similar to peptidylprolyl isomerase A isoform 1 (LOC730850), mRNA [XR_015255] | 2.15 |
| ASCC3 | Homo sapiens activating signal cointegrator 1 complex subunit 3 (ASCC3), transcript variant 1, mRNA [NM_006828] | 2.15 |
| ALS2CR13 | Homo sapiens amyotrophic lateral sclerosis 2 (juvenile) chromosome region, candidate 13 (ALS2CR13), mRNA [NM_173511] | 2.16 |
| GPSM3 | Homo sapiens G-protein signalling modulator 3 (AGS3-like, C. elegans) (GPSM3), mRNA [NM_022107] | 2.16 |
| SGK | Homo sapiens serum/glucocorticoid regulated kinase (SGK), mRNA [NM_005627] | 2.16 |
| SMG5 | Homo sapiens Smg-5 homolog, nonsense mediated mRNA decay factor (C. elegans) (SMG5), mRNA [NM_015327] | 2.16 |
| SFRS8 | Homo sapiens splicing factor, arginine/serine-rich 8 (suppressor-of-white-apricot homolog, Drosophila) (SFRS8), mRNA [NM_004592] | 2.16 |
| MED18 | Homo sapiens mediator of RNA polymerase II transcription, subunit 18 homolog (S. cerevisiae) (MED18), mRNA [NM_017638] | 2.16 |
| ADAR | Homo sapiens adenosine deaminase, RNA-specific (ADAR), transcript variant 1, mRNA [NM_001111] | 2.16 |
| MIA3 | full-length cDNA clone CS0DI033YD20 of Placenta Cot 25-normalized of Homo sapiens (human). [CR613982] | 2.16 |
| LOC92312 | Homo sapiens cDNA FLJ43493 fis, clone OCBBF3009279. [AK125482] | 2.16 |
| ENST00000273582 | full-length cDNA clone CS0DI044YK15 of Placenta Cot 25-normalized of Homo sapiens (human). [CR601728] | 2.16 |
| FBXL10 | Homo sapiens F-box and leucine-rich repeat protein 10 (FBXL10), transcript variant 1, mRNA [NM_032590] | 2.16 |
| OXCT1 | Homo sapiens 3-oxoacid CoA transferase 1 (OXCT1), nuclear gene encoding mitochondrial protein, mRNA [NM_000436] | 2.16 |
| ERBB2IP | Homo sapiens erbb2 interacting protein (ERBB2IP), transcript variant 2, mRNA [NM_018695] | 2.16 |
| EIF4G1 | Homo sapiens eukaryotic translation initiation factor 4 gamma, 1 (EIF4G1), transcript variant 1, mRNA [NM_182917] | 2.17 |
| DSG2 | Homo sapiens desmoglein 2 (DSG2), mRNA [NM_001943] | 2.17 |
| DNAJC14 | Homo sapiens DnaJ (Hsp40) homolog, subfamily C, member 14 (DNAJC14), mRNA [NM_032364] | 2.17 |
| GM632 | Homo sapiens KIAA1196 protein (GM632), mRNA [NM_020713] | 2.17 |
| A_24_P135515 | Unknown | 2.17 |
| LEPR | Homo sapiens leptin receptor (LEPR), transcript variant 2, mRNA [NM_001003679] | 2.17 |
| THC2617699 | AL353671 match: proteins: BAC05300 {Homo sapiens} (exp=0; wgp=1; cg=0), partial (12%) [THC2617699] | 2.17 |
| C17orf27 | Homo sapiens chromosome 17 open reading frame 27 (C17orf27), mRNA [NM_020914] | 2.17 |
| TMTC4 | Homo sapiens transmembrane and tetratricopeptide repeat containing 4 (TMTC4), transcript variant 1, mRNA [NM_032813] | 2.17 |
| TMEM48 | Homo sapiens cDNA FLJ34120 fis, clone FCBBF3009541. [AK091439] | 2.18 |
| A_24_P323778 | Unknown | 2.18 |
| RIOK1 | Homo sapiens RIO kinase 1 (yeast) (RIOK1), transcript variant 1, mRNA [NM_031480] | 2.18 |
| PCBP2 | Homo sapiens poly(rC) binding protein 2 (PCBP2), transcript variant 2, mRNA [NM_031989] | 2.18 |
| C10orf38 | Homo sapiens chromosome 10 open reading frame 38 (C10orf38), mRNA [NM_001010924] | 2.18 |
| ENST00000311275 | Protein kinase C-binding protein 1 (Rack7) (Cutaneous T-cell lymphoma- associated antigen se14-3) (CTCL tumor antigen se14-3) (Zinc finger MYND domain-containing protein 8). [Source:Uniprot/SWISSPROT;Acc:Q9ULU4] [ENST00000311275] | 2.18 |
| ANAPC5 | Homo sapiens anaphase promoting complex subunit 5 (ANAPC5), mRNA [NM_016237] | 2.18 |
| FLJ10324 | Homo sapiens hypothetical protein FLJ10324 (FLJ10324), mRNA [NM_018059] | 2.18 |
| ZNF639 | Homo sapiens zinc finger protein 639 (ZNF639), mRNA [NM_016331] | 2.18 |
| KIAA0528 | Homo sapiens KIAA0528 (KIAA0528), mRNA [NM_014802] | 2.18 |
| CD46 | Homo sapiens CD46 molecule, complement regulatory protein (CD46), transcript variant a, mRNA [NM_002389] | 2.19 |
| GABRE | Homo sapiens gamma-aminobutyric acid (GABA) A receptor, epsilon (GABRE), transcript variant 4, mRNA [NM_021990] | 2.19 |
| GNAS | Homo sapiens GNAS complex locus (GNAS), transcript variant 7, mRNA [NM_001077489] | 2.19 |
| DNAJB12 | Homo sapiens DnaJ (Hsp40) homolog, subfamily B, member 12 (DNAJB12), transcript variant 2, mRNA [NM_017626] | 2.19 |
| TMEM64 | Homo sapiens cDNA FLJ38153 fis, clone DFNES1000083. [AK095472] | 2.19 |
| AL117599 | Homo sapiens mRNA; cDNA DKFZp564I0463 (from clone DKFZp564I0463). [AL117599] | 2.19 |
| ST13 | Homo sapiens suppression of tumorigenicity 13 (colon carcinoma) (Hsp70 interacting protein) (ST13), mRNA [NM_003932] | 2.19 |
| PPP4R2 | Homo sapiens protein phosphatase 4, regulatory subunit 2 (PPP4R2), mRNA [NM_174907] | 2.19 |
| EFHC2 | Homo sapiens EF-hand domain (C-terminal) containing 2 (EFHC2), mRNA [NM_025184] | 2.20 |
| OBSCN | Homo sapiens cDNA FLJ14124 fis, clone MAMMA1002498. [AK024186] | 2.20 |
| LUC7L | Homo sapiens LUC7-like (S. cerevisiae) (LUC7L), transcript variant 2, mRNA [NM_201412] | 2.20 |
| SPRY2 | Homo sapiens sprouty homolog 2 (Drosophila) (SPRY2), mRNA [NM_005842] | 2.20 |
| C12orf52 | Homo sapiens chromosome 12 open reading frame 52 (C12orf52), mRNA [NM_032848] | 2.20 |
| NFKBIB | Homo sapiens nuclear factor of kappa light polypeptide gene enhancer in B-cells inhibitor, beta (NFKBIB), transcript variant 2, mRNA [NM_001001716] | 2.20 |
| TTLL4 | Homo sapiens tubulin tyrosine ligase-like family, member 4 (TTLL4), mRNA [NM_014640] | 2.20 |
| THC2519808 | Unknown | 2.20 |
| LOC729313 | PREDICTED: Homo sapiens similar to peptidylprolyl isomerase A isoform 1 (LOC729313), mRNA [XR_015971] | 2.20 |
| LOC651462 | PREDICTED: Homo sapiens similar to 60 kDa heat shock protein, mitochondrial precursor (Hsp60) (60 kDa chaperonin) (CPN60) (Heat shock protein 60) (HSP-60) (Mitochondrial matrix protein P1) (P60 lymphocyte protein) (HuCHA60) (LOC651462), mRNA [XR_019400] | 2.20 |
| ITM2A | Homo sapiens integral membrane protein 2A (ITM2A), mRNA [NM_004867] | 2.20 |
| WDR1 | Homo sapiens WD repeat domain 1 (WDR1), transcript variant 1, mRNA [NM_017491] | 2.21 |
| FAM82A | Homo sapiens family with sequence similarity 82, member A (FAM82A), mRNA [NM_144713] | 2.21 |
| TNIK | Homo sapiens mRNA for KIAA0551 protein, partial cds. [AB011123] | 2.21 |
| CDC42BPA | Homo sapiens CDC42 binding protein kinase alpha (DMPK-like) (CDC42BPA), transcript variant A, mRNA [NM_014826] | 2.21 |
| LOC650031 | PREDICTED: Homo sapiens similar to peptidylprolyl isomerase A isoform 1 (LOC650031), mRNA [XR_018815] | 2.21 |
| BAZ1B | Homo sapiens bromodomain adjacent to zinc finger domain, 1B (BAZ1B), mRNA [NM_032408] | 2.21 |
| PPP4R2 | Homo sapiens protein phosphatase 4, regulatory subunit 2 (PPP4R2), mRNA [NM_174907] | 2.21 |
| IGF2BP2 | Homo sapiens insulin-like growth factor 2 mRNA binding protein 2 (IGF2BP2), transcript variant 1, mRNA [NM_006548] | 2.21 |
| PPIA | Homo sapiens peptidylprolyl isomerase A (cyclophilin A) (PPIA), mRNA [NM_021130] | 2.21 |
| CTDSP1 | Homo sapiens CTD (carboxy-terminal domain, RNA polymerase II, polypeptide A) small phosphatase 1 (CTDSP1), transcript variant 1, mRNA [NM_021198] | 2.22 |
| ENST00000330469 | T-box transcription factor TBX18 (T-box protein 18). [Source:Uniprot/SWISSPROT;Acc:O95935] [ENST00000330469] | 2.22 |
| ZNF174 | Homo sapiens zinc finger protein 174 (ZNF174), transcript variant 1, mRNA [NM_003450] | 2.22 |
| CAMKK2 | Homo sapiens calcium/calmodulin-dependent protein kinase kinase 2, beta (CAMKK2), transcript variant 1, mRNA [NM_006549] | 2.22 |
| PIK4CB | Homo sapiens phosphatidylinositol 4-kinase, catalytic, beta polypeptide (PIK4CB), mRNA [NM_002651] | 2.22 |
| GOLGA4 | Homo sapiens golgi autoantigen, golgin subfamily a, 4 (GOLGA4), mRNA [NM_002078] | 2.22 |
| KIAA1274 | Homo sapiens KIAA1274 (KIAA1274), mRNA [NM_014431] | 2.22 |
| BIVM | Homo sapiens basic, immunoglobulin-like variable motif containing (BIVM), mRNA [NM_017693] | 2.22 |
| LOC401859 | PREDICTED: Homo sapiens similar to peptidylprolyl isomerase A isoform 1 (LOC401859), mRNA [XR_017566] | 2.22 |
| BF895757 | BF895757 RC3-MT0162-221100-012-h03 MT0162 Homo sapiens cDNA, mRNA sequence [BF895757] | 2.23 |
| HSPE1 | Homo sapiens heat shock 10kDa protein 1 (chaperonin 10) (HSPE1), mRNA [NM_002157] | 2.23 |
| NCL | Homo sapiens nucleolin (NCL), mRNA [NM_005381] | 2.23 |
| UTP20 | Homo sapiens UTP20, small subunit (SSU) processome component, homolog (yeast) (UTP20), mRNA [NM_014503] | 2.24 |
| C21orf66 | Homo sapiens cDNA clone IMAGE:5497083, containing frame-shift errors. [BC062992] | 2.24 |
| LOC442336 | PREDICTED: Homo sapiens similar to peptidylprolyl isomerase A isoform 1 (LOC442336), mRNA [XR_018032] | 2.24 |
| NUCKS1 | Nuclear ubiquitous casein and cyclin-dependent kinases substrate (P1). [Source:Uniprot/SWISSPROT;Acc:Q9H1E3] [ENST00000367142] | 2.24 |
| BBC3 | Homo sapiens BCL2 binding component 3 (BBC3), mRNA [NM_014417] | 2.24 |
| GABPB2 | Homo sapiens GA binding protein transcription factor, beta subunit 2 (GABPB2), transcript variant beta-1, mRNA [NM_005254] | 2.25 |
| COX1 | Cytochrome c oxidase subunit 1 (EC 1.9.3.1) (Cytochrome c oxidase polypeptide I). [Source:Uniprot/SWISSPROT;Acc:P00395] [ENST00000361624] | 2.25 |
| MDN1 | Homo sapiens MDN1, midasin homolog (yeast) (MDN1), mRNA [NM_014611] | 2.25 |
| LOC202319 | PREDICTED: Homo sapiens similar to peptidylprolyl isomerase A isoform 1 (LOC202319), mRNA [XR_016130] | 2.25 |
| RASGEF1A | Homo sapiens RasGEF domain family, member 1A (RASGEF1A), mRNA [NM_145313] | 2.25 |
| CNN2 | Homo sapiens calponin 2 (CNN2), transcript variant 1, mRNA [NM_004368] | 2.26 |
| A_24_P332326 | Unknown | 2.26 |
| STEAP3 | Homo sapiens STEAP family member 3 (STEAP3), transcript variant 1, mRNA [NM_182915] | 2.26 |
| FN1 | Homo sapiens fibronectin 1 (FN1), transcript variant 1, mRNA [NM_212482] | 2.26 |
| DDX46 | Homo sapiens DEAD (Asp-Glu-Ala-Asp) box polypeptide 46 (DDX46), mRNA [NM_014829] | 2.26 |
| KRTAP4-7 | Homo sapiens mRNA; cDNA DKFZp686I16170 (from clone DKFZp686I16170). [BX648343] | 2.26 |
| VWA1 | Homo sapiens von Willebrand factor A domain containing 1 (VWA1), transcript variant 1, mRNA [NM_022834] | 2.26 |
| CITED2 | Homo sapiens Cbp/p300-interacting transactivator, with Glu/Asp-rich carboxy-terminal domain, 2 (CITED2), mRNA [NM_006079] | 2.27 |
| FAM89A | Homo sapiens family with sequence similarity 89, member A (FAM89A), mRNA [NM_198552] | 2.27 |
| JARID2 | Homo sapiens jumonji, AT rich interactive domain 2 (JARID2), mRNA [NM_004973] | 2.27 |
| CR622342 | full-length cDNA clone CS0DJ006YC05 of T cells (Jurkat cell line) Cot 10-normalized of Homo sapiens (human). [CR622342] | 2.27 |
| NETO2 | Homo sapiens neuropilin (NRP) and tolloid (TLL)-like 2 (NETO2), mRNA [NM_018092] | 2.27 |
| KCTD12 | Homo sapiens potassium channel tetramerisation domain containing 12 (KCTD12), mRNA [NM_138444] | 2.27 |
| ASAHL | Homo sapiens N-acylsphingosine amidohydrolase (acid ceramidase)-like (ASAHL), transcript variant 1, mRNA [NM_014435] | 2.27 |
| PPIAL4 | Homo sapiens peptidylprolyl isomerase A (cyclophilin A)-like 4 (PPIAL4), mRNA [NM_178230] | 2.28 |
| ELF1 | Homo sapiens E74-like factor 1 (ets domain transcription factor) (ELF1), mRNA [NM_172373] | 2.28 |
| ACTG1 | Homo sapiens actin, gamma 1 (ACTG1), mRNA [NM_001614] | 2.28 |
| CDC42SE1 | Homo sapiens CDC42 small effector 1 (CDC42SE1), transcript variant 1, mRNA [NM_001038707] | 2.29 |
| A_24_P170103 | Unknown | 2.29 |
| KLHL17 | Homo sapiens kelch-like 17 (Drosophila) (KLHL17), mRNA [NM_198317] | 2.29 |
| CXXC1 | Homo sapiens CXXC finger 1 (PHD domain) (CXXC1), mRNA [NM_014593] | 2.29 |
| ZNF588 | Homo sapiens zinc finger protein 588 (ZNF588), transcript variant 1, mRNA [NM_016220] | 2.29 |
| RBM17 | Homo sapiens RNA binding motif protein 17 (RBM17), mRNA [NM_032905] | 2.29 |
| PERQ1 | Homo sapiens PERQ amino acid rich, with GYF domain 1 (PERQ1), mRNA [NM_022574] | 2.30 |
| THC2503819 | BC010854 amyloid beta A4 precursor protein-binding, family B, member 1, isoform delta E9 {Homo sapiens} (exp=-1; wgp=0; cg=0), partial (22%) [THC2503819] | 2.30 |
| FAM82A | Homo sapiens unknown mRNA. [AF435956] | 2.30 |
| AK098081 | Homo sapiens cDNA FLJ40762 fis, clone TRACH2002847. [AK098081] | 2.30 |
| GNB2 | Homo sapiens guanine nucleotide binding protein (G protein), beta polypeptide 2 (GNB2), mRNA [NM_005273] | 2.30 |
| EWSR1 | Homo sapiens Ewing sarcoma breakpoint region 1 (EWSR1), transcript variant EWS-b, mRNA [NM_013986] | 2.30 |
| CBFB | Homo sapiens core-binding factor, beta subunit (CBFB), transcript variant 2, mRNA [NM_001755] | 2.31 |
| PNMT | Homo sapiens phenylethanolamine N-methyltransferase (PNMT), mRNA [NM_002686] | 2.31 |
| KBTBD2 | Homo sapiens kelch repeat and BTB (POZ) domain containing 2 (KBTBD2), mRNA [NM_015483] | 2.31 |
| USP15 | Homo sapiens ubiquitin specific peptidase 15 (USP15), mRNA [NM_006313] | 2.31 |
| PLAU | Homo sapiens plasminogen activator, urokinase (PLAU), mRNA [NM_002658] | 2.32 |
| TMEM41A | Homo sapiens transmembrane protein 41A (TMEM41A), mRNA [NM_080652] | 2.32 |
| A_24_P852099 | Unknown | 2.32 |
| PDE6D | Homo sapiens phosphodiesterase 6D, cGMP-specific, rod, delta (PDE6D), mRNA [NM_002601] | 2.32 |
| EXOC7 | Homo sapiens exocyst complex component 7 (EXOC7), transcript variant 1, mRNA [NM_001013839] | 2.32 |
| AK057015 | Homo sapiens cDNA FLJ32453 fis, clone SKMUS2001703. [AK057015] | 2.32 |
| RCSD1 | Homo sapiens RCSD domain containing 1 (RCSD1), mRNA [NM_052862] | 2.32 |
| KIAA0152 | Homo sapiens KIAA0152 (KIAA0152), mRNA [NM_014730] | 2.33 |
| USP47 | Homo sapiens ubiquitin specific peptidase 47 (USP47), mRNA [NM_017944] | 2.33 |
| CTGLF5 | Homo sapiens centaurin, gamma-like family, member 5 (CTGLF5), mRNA [NM_001077686] | 2.33 |
| ARL6IP5 | Homo sapiens ADP-ribosylation-like factor 6 interacting protein 5 (ARL6IP5), mRNA [NM_006407] | 2.33 |
| HDDC2 | Homo sapiens HD domain containing 2 (HDDC2), mRNA [NM_016063] | 2.33 |
| ZNF498 | Homo sapiens zinc finger protein 498 (ZNF498), mRNA [NM_145115] | 2.33 |
| SLMAP | Homo sapiens sarcolemma associated protein (SLMAP), mRNA [NM_007159] | 2.33 |
| ICA1 | Homo sapiens islet cell autoantigen 1, 69kDa (ICA1), transcript variant 2, mRNA [NM_004968] | 2.33 |
| ZFP64 | Homo sapiens zinc finger protein 64 homolog (mouse) (ZFP64), transcript variant 1, mRNA [NM_018197] | 2.34 |
| RFXAP | Homo sapiens regulatory factor X-associated protein (RFXAP), mRNA [NM_000538] | 2.34 |
| NCOA7 | Homo sapiens nuclear receptor coactivator 7 (NCOA7), mRNA [NM_181782] | 2.34 |
| DES | Homo sapiens desmin (DES), mRNA [NM_001927] | 2.34 |
| BCL3 | Homo sapiens B-cell CLL/lymphoma 3 (BCL3), mRNA [NM_005178] | 2.34 |
| SFRS2 | Homo sapiens splicing factor, arginine/serine-rich 2 (SFRS2), mRNA [NM_003016] | 2.35 |
| ENST00000326470 | Protein phosphatase Slingshot homolog 1 (EC 3.1.3.48) (EC 3.1.3.16) (SSH-1L) (hSSH-1L). [Source:Uniprot/SWISSPROT;Acc:Q8WYL5] [ENST00000326470] | 2.35 |
| LOC441768 | PREDICTED: Homo sapiens similar to RAB31, member RAS oncogene family (LOC441768), mRNA [XR_019199] | 2.35 |
| DAG1 | Homo sapiens dystroglycan 1 (dystrophin-associated glycoprotein 1) (DAG1), mRNA [NM_004393] | 2.35 |
| TBL3 | Homo sapiens transducin (beta)-like 3 (TBL3), mRNA [NM_006453] | 2.35 |
| LOC92312 | PREDICTED: Homo sapiens hypothetical protein LOC92312 (LOC92312), mRNA [XM_044166] | 2.35 |
| KIAA1666 | Homo sapiens KIAA1666 protein, mRNA (cDNA clone IMAGE:4827837), complete cds. [BC035246] | 2.36 |
| NUP153 | Homo sapiens nucleoporin 153kDa (NUP153), mRNA [NM_005124] | 2.36 |
| ZNF217 | Homo sapiens zinc finger protein 217 (ZNF217), mRNA [NM_006526] | 2.36 |
| NETO2 | Homo sapiens neuropilin (NRP) and tolloid (TLL)-like 2 (NETO2), mRNA [NM_018092] | 2.36 |
| GON4L | Homo sapiens gon-4-like (C. elegans) (GON4L), transcript variant 1, mRNA [NM_001037533] | 2.36 |
| BC033829 | Homo sapiens cDNA clone IMAGE:3856003, partial cds. [BC033829] | 2.36 |
| CCDC92 | Homo sapiens coiled-coil domain containing 92 (CCDC92), mRNA [NM_025140] | 2.36 |
| PRPF4B | Homo sapiens PRP4 pre-mRNA processing factor 4 homolog B (yeast) (PRPF4B), mRNA [NM_003913] | 2.36 |
| RP5-1022P6.2 | Homo sapiens hypothetical protein KIAA1434 (KIAA1434), mRNA [NM_019593] | 2.37 |
| SATB2 | Homo sapiens SATB family member 2 (SATB2), mRNA [NM_015265] | 2.37 |
| AK054852 | Homo sapiens cDNA FLJ30290 fis, clone BRACE2002884. [AK054852] | 2.37 |
| CCT6AP1 | Homo sapiens cDNA FLJ34861 fis, clone NT2NE2012847. [AK092180] | 2.38 |
| TOMM20 | Homo sapiens translocase of outer mitochondrial membrane 20 homolog (yeast) (TOMM20), mRNA [NM_014765] | 2.38 |
| ECM1 | Homo sapiens extracellular matrix protein 1 (ECM1), transcript variant 1, mRNA [NM_004425] | 2.38 |
| PIP5K3 | Homo sapiens phosphatidylinositol-3-phosphate/phosphatidylinositol 5-kinase, type III (PIP5K3), transcript variant 2, mRNA [NM_015040] | 2.39 |
| CYP51A1 | Homo sapiens cytochrome P450, family 51, subfamily A, polypeptide 1 (CYP51A1), mRNA [NM_000786] | 2.39 |
| FN1 | Homo sapiens fibronectin 1 (FN1), transcript variant 1, mRNA [NM_212482] | 2.39 |
| AK074291 | Homo sapiens cDNA FLJ23711 fis, clone HEP12371. [AK074291] | 2.39 |
| PTMA | Homo sapiens prothymosin, alpha (gene sequence 28) (PTMA), mRNA [NM_002823] | 2.39 |
| RHOT1 | Homo sapiens ras homolog gene family, member T1 (RHOT1), transcript variant 4, mRNA [NM_001033567] | 2.40 |
| EWSR1 | Homo sapiens Ewing sarcoma breakpoint region 1 (EWSR1), transcript variant EWS-b, mRNA [NM_013986] | 2.40 |
| PILRB | Homo sapiens paired immunoglobin-like type 2 receptor beta (PILRB), transcript variant 1, mRNA [NM_013440] | 2.40 |
| MPHOSPH1 | Homo sapiens M-phase phosphoprotein 1 (MPHOSPH1), mRNA [NM_016195] | 2.41 |
| CNKSR3 | Homo sapiens CNKSR family member 3 (CNKSR3), mRNA [NM_173515] | 2.41 |
| ZNF529 | Homo sapiens zinc finger protein 529 (ZNF529), mRNA [NM_020951] | 2.41 |
| DKFZp586I1420 | Homo sapiens hypothetical protein DKFZp586I1420 (DKFZp586I1420) on chromosome 7 [NR_002186] | 2.41 |
| NFKBIA | Homo sapiens nuclear factor of kappa light polypeptide gene enhancer in B-cells inhibitor, alpha (NFKBIA), mRNA [NM_020529] | 2.41 |
| CUGBP2 | Homo sapiens CUG triplet repeat, RNA binding protein 2 (CUGBP2), transcript variant 1, mRNA [NM_001025076] | 2.41 |
| EEA1 | Homo sapiens early endosome antigen 1, 162kD (EEA1), mRNA [NM_003566] | 2.42 |
| GNB1 | Homo sapiens guanine nucleotide binding protein (G protein), beta polypeptide 1 (GNB1), mRNA [NM_002074] | 2.42 |
| CLCN2 | Homo sapiens chloride channel 2 (CLCN2), mRNA [NM_004366] | 2.42 |
| GNAQ | Guanine nucleotide-binding protein G(q) subunit alpha (Guanine nucleotide-binding protein alpha-q). [Source:Uniprot/SWISSPROT;Acc:P50148] [ENST00000376611] | 2.42 |
| GLS | Homo sapiens glutaminase C mRNA, complete cds. [AF158555] | 2.42 |
| MMD | Homo sapiens monocyte to macrophage differentiation-associated (MMD), mRNA [NM_012329] | 2.42 |
| C5orf25 | Homo sapiens chromosome 5 open reading frame 25 (C5orf25), mRNA [NM_198567] | 2.42 |
| LPGAT1 | Homo sapiens lysophosphatidylglycerol acyltransferase 1 (LPGAT1), mRNA [NM_014873] | 2.43 |
| NACA | Homo sapiens nascent-polypeptide-associated complex alpha polypeptide (NACA), mRNA [NM_005594] | 2.43 |
| SLC30A5 | Homo sapiens mRNA; cDNA DKFZp686F23142 (from clone DKFZp686F23142); complete cds. [BX537394] | 2.44 |
| AB040974 | Homo sapiens mRNA for KIAA1541 protein, partial cds. [AB040974] | 2.44 |
| GYPA | Homo sapiens glycophorin A (MNS blood group) (GYPA), mRNA [NM_002099] | 2.44 |
| CTGLF4 | Homo sapiens centaurin, gamma-like family, member 4 (CTGLF4), mRNA [NM_001077685] | 2.44 |
| TBC1D24 | Homo sapiens mRNA; cDNA DKFZp686J0866 (from clone DKFZp686J0866). [BX648283] | 2.45 |
| TM9SF3 | Transmembrane 9 superfamily protein member 3 precursor (SM-11044- binding protein) (EP70-P-iso). [Source:Uniprot/SWISSPROT;Acc:Q9HD45] [ENST00000371142] | 2.45 |
| ANKRD11 | Homo sapiens ankyrin repeat domain 11 (ANKRD11), mRNA [NM_013275] | 2.45 |
| ESPL1 | Homo sapiens extra spindle pole bodies homolog 1 (S. cerevisiae) (ESPL1), mRNA [NM_012291] | 2.46 |
| AK092083 | Homo sapiens cDNA FLJ34764 fis, clone NT2NE2002311. [AK092083] | 2.46 |
| GNAS | Homo sapiens GNAS complex locus (GNAS), transcript variant 7, mRNA [NM_001077489] | 2.46 |
| SACM1L | Homo sapiens SAC1 suppressor of actin mutations 1-like (yeast) (SACM1L), mRNA [NM_014016] | 2.46 |
| LOC653364 | PREDICTED: Homo sapiens similar to Heat-shock protein beta-1 (HspB1) (Heat shock 27 kDa protein) (HSP 27) (Stress-responsive protein 27) (SRP27) (Estrogen-regulated 24 kDa protein) (28 kDa heat shock protein) (LOC653364), mRNA [XR_017030] | 2.47 |
| ESCO1 | Homo sapiens establishment of cohesion 1 homolog 1 (S. cerevisiae) (ESCO1), mRNA [NM_052911] | 2.47 |
| SGOL2 | Homo sapiens shugoshin-like 2 (S. pombe) (SGOL2), mRNA [NM_152524] | 2.47 |
| SPTA1 | Homo sapiens spectrin, alpha, erythrocytic 1 (elliptocytosis 2) (SPTA1), mRNA [NM_003126] | 2.47 |
| CENPJ | Homo sapiens centromere protein J (CENPJ), mRNA [NM_018451] | 2.47 |
| MALAT1 | Homo sapiens metastasis associated lung adenocarcinoma transcript 1 (non-coding RNA) (MALAT1) on chromosome 11 [NR_002819] | 2.47 |
| IRS1 | Homo sapiens insulin receptor substrate 1 (IRS1), mRNA [NM_005544] | 2.47 |
| RPL6 | Homo sapiens ribosomal protein L6 (RPL6), transcript variant 1, mRNA [NM_001024662] | 2.48 |
| PSEN2 | Homo sapiens presenilin 2 (Alzheimer disease 4) (PSEN2), transcript variant 2, mRNA [NM_012486] | 2.48 |
| AL117621 | Homo sapiens mRNA; cDNA DKFZp564M0264 (from clone DKFZp564M0264). [AL117621] | 2.48 |
| FLJ34077 | Homo sapiens weakly similar to zinc finger protein 195, mRNA (cDNA clone IMAGE:3606289), partial cds. [BC003519] | 2.48 |
| THC2682280 | Unknown | 2.48 |
| C18orf2 | Homo sapiens cDNA FLJ32943 fis, clone TESTI2007829. [AK057505] | 2.49 |
| TBCCD1 | Homo sapiens TBCC domain containing 1 (TBCCD1), mRNA [NM_018138] | 2.49 |
| RHOBTB3 | Homo sapiens Rho-related BTB domain containing 3 (RHOBTB3), mRNA [NM_014899] | 2.49 |
| THC2510124 | Q41JZ9_METBU (Q41JZ9) UbiA prenyltransferase, partial (7%) [THC2510124] | 2.49 |
| ABCC5 | Homo sapiens ATP-binding cassette, sub-family C (CFTR/MRP), member 5 (ABCC5), transcript variant 1, mRNA [NM_005688] | 2.50 |
| MAP4K4 | Homo sapiens mitogen-activated protein kinase kinase kinase kinase 4 (MAP4K4), transcript variant 2, mRNA [NM_145686] | 2.50 |
| 2'-PDE | Homo sapiens 2'-phosphodiesterase (2'-PDE), mRNA [NM_177966] | 2.50 |
| KIAA0528 | Homo sapiens KIAA0528 (KIAA0528), mRNA [NM_014802] | 2.50 |
| HLTF | Homo sapiens helicase-like transcription factor (HLTF), transcript variant 2, mRNA [NM_139048] | 2.50 |
| A_32_P26443 | Unknown | 2.50 |
| PAK2 | Homo sapiens p21 (CDKN1A)-activated kinase 2 (PAK2), mRNA [NM_002577] | 2.51 |
| XRCC6BP1 | Homo sapiens XRCC6 binding protein 1 (XRCC6BP1), mRNA [NM_033276] | 2.51 |
| OPA1 | Homo sapiens optic atrophy 1 (autosomal dominant) (OPA1), nuclear gene encoding mitochondrial protein, transcript variant 8, mRNA [NM_130837] | 2.51 |
| P18SRP | Homo sapiens P18SRP protein (P18SRP), mRNA [NM_173829] | 2.51 |
| CTBP2 | Homo sapiens C-terminal binding protein 2 (CTBP2), transcript variant 2, mRNA [NM_022802] | 2.52 |
| SUB1 | Activated RNA polymerase II transcriptional coactivator p15 (SUB1 homolog) (Positive cofactor 4) (PC4) (p14). [Source:Uniprot/SWISSPROT;Acc:P53999] [ENST00000265073] | 2.52 |
| CR608275 | full-length cDNA clone CS0CAP007YE04 of Thymus of Homo sapiens (human). [CR608275] | 2.52 |
| THC2683448 | Q7WZG3_PASPI (Q7WZG3) Ferric uptake regulator, partial (8%) [THC2683448] | 2.53 |
| LOC645808 | PREDICTED: Homo sapiens similar to 60 kDa heat shock protein, mitochondrial precursor (Hsp60) (60 kDa chaperonin) (CPN60) (Heat shock protein 60) (HSP-60) (Mitochondrial matrix protein P1) (P60 lymphocyte protein) (HuCHA60) (LOC645808), mRNA [XR_016831] | 2.53 |
| PPIAL4 | Homo sapiens peptidylprolyl isomerase A (cyclophilin A)-like 4 (PPIAL4), mRNA [NM_178230] | 2.54 |
| HDAC4 | Homo sapiens histone deacetylase 4 (HDAC4), mRNA [NM_006037] | 2.54 |
| NUCKS1 | Nuclear ubiquitous casein and cyclin-dependent kinases substrate (P1). [Source:Uniprot/SWISSPROT;Acc:Q9H1E3] [ENST00000367142] | 2.55 |
| LOC400036 | PREDICTED: Homo sapiens similar to DEAD (Asp-Glu-Ala-Asp) box polypeptide 23 (LOC400036), mRNA [XR_016505] | 2.55 |
| ATF6 | Cyclic AMP-dependent transcription factor ATF-6 alpha (Activating transcription factor 6 alpha) (ATF6-alpha). [Source:Uniprot/SWISSPROT;Acc:P18850] [ENST00000367942] | 2.55 |
| ACSL4 | Homo sapiens acyl-CoA synthetase long-chain family member 4 (ACSL4), transcript variant 1, mRNA [NM_004458] | 2.55 |
| TRIM13 | Homo sapiens tripartite motif-containing 13 (TRIM13), transcript variant 3, mRNA [NM_213590] | 2.55 |
| TBL1XR1 | Homo sapiens transducin (beta)-like 1X-linked receptor 1 (TBL1XR1), mRNA [NM_024665] | 2.55 |
| CNOT2 | Homo sapiens CCR4-NOT transcription complex, subunit 2 (CNOT2), mRNA [NM_014515] | 2.56 |
| TPM3 | Homo sapiens tropomyosin 3 (TPM3), transcript variant 3, mRNA [NM_001043352] | 2.56 |
| UBL3 | Homo sapiens ubiquitin-like 3 (UBL3), mRNA [NM_007106] | 2.56 |
| THC2757997 | Unknown | 2.56 |
| AK026215 | Homo sapiens cDNA: FLJ22562 fis, clone HSI01814. [AK026215] | 2.57 |
| ZNF521 | Homo sapiens zinc finger protein 521 (ZNF521), mRNA [NM_015461] | 2.57 |
| KIAA1815 | Homo sapiens KIAA1815 (KIAA1815), mRNA [NM_024896] | 2.58 |
| USP54 | Homo sapiens cDNA FLJ45138 fis, clone BRAWH3039258. [AK127081] | 2.58 |
| ENST00000308894 | Homo sapiens HSPC324 mRNA, partial cds. [AF161442] | 2.58 |
| RGS2 | Homo sapiens regulator of G-protein signalling 2, 24kDa (RGS2), mRNA [NM_002923] | 2.59 |
| BQ424374 | AGENCOURT_7892842 NIH_MGC_72 Homo sapiens cDNA clone IMAGE:6157378 5', mRNA sequence [BQ424374] | 2.59 |
| ATP2B1 | Homo sapiens ATPase, Ca++ transporting, plasma membrane 1 (ATP2B1), transcript variant 2, mRNA [NM_001682] | 2.59 |
| NAB2 | Homo sapiens NGFI-A binding protein 2 (EGR1 binding protein 2) (NAB2), mRNA [NM_005967] | 2.60 |
| CXXC6 | CXXC-type zinc finger protein 6 (Leukemia-associated protein with a CXXC domain). [Source:Uniprot/SWISSPROT;Acc:Q8NFU7] [ENST00000373644] | 2.60 |
| ERMAP | Homo sapiens erythroblast membrane-associated protein (Scianna blood group) (ERMAP), transcript variant 1, mRNA [NM_001017922] | 2.60 |
| RAB31 | Homo sapiens RAB31, member RAS oncogene family (RAB31), mRNA [NM_006868] | 2.60 |
| LRP8 | Homo sapiens low density lipoprotein receptor-related protein 8, apolipoprotein e receptor (LRP8), transcript variant 2, mRNA [NM_033300] | 2.60 |
| SLC39A10 | Homo sapiens solute carrier family 39 (zinc transporter), member 10 (SLC39A10), mRNA [NM_020342] | 2.60 |
| TFDP1 | Homo sapiens transcription factor Dp-1 (TFDP1), mRNA [NM_007111] | 2.60 |
| ZNF138 | Homo sapiens zinc finger protein 138 (ZNF138), mRNA [NM_006524] | 2.60 |
| RAB31 | Homo sapiens RAB31, member RAS oncogene family (RAB31), mRNA [NM_006868] | 2.61 |
| ZNF595 | Homo sapiens zinc finger protein 595 (ZNF595), mRNA [NM_182524] | 2.61 |
| CDCA7L | Homo sapiens cell division cycle associated 7-like (CDCA7L), mRNA [NM_018719] | 2.61 |
| RAB11A | Homo sapiens RAB11A, member RAS oncogene family (RAB11A), mRNA [NM_004663] | 2.61 |
| POLR3C | Homo sapiens polymerase (RNA) III (DNA directed) polypeptide C (62kD) (POLR3C), mRNA [NM_006468] | 2.62 |
| SFRS2IP | Homo sapiens splicing factor, arginine/serine-rich 2, interacting protein (SFRS2IP), mRNA [NM_004719] | 2.63 |
| CR603272 | full-length cDNA clone CS0DC013YI10 of Neuroblastoma Cot 25-normalized of Homo sapiens (human). [CR603272] | 2.63 |
| REEP3 | Receptor expression-enhancing protein 3. [Source:Uniprot/SWISSPROT;Acc:Q6NUK4] [ENST00000298249] | 2.63 |
| RASGEF1A | Homo sapiens RasGEF domain family, member 1A, mRNA (cDNA clone MGC:26821 IMAGE:4814750), complete cds. [BC022548] | 2.64 |
| TMEM168 | Homo sapiens transmembrane protein 168 (TMEM168), mRNA [NM_022484] | 2.64 |
| TRAF5 | Homo sapiens TNF receptor-associated factor 5 (TRAF5), transcript variant 1, mRNA [NM_004619] | 2.65 |
| ARID5B | Homo sapiens AT rich interactive domain 5B (MRF1-like) (ARID5B), mRNA [NM_032199] | 2.65 |
| TNFRSF8 | Homo sapiens tumor necrosis factor receptor superfamily, member 8 (TNFRSF8), transcript variant 1, mRNA [NM_001243] | 2.65 |
| C20orf45 | Homo sapiens chromosome 20 open reading frame 45 (C20orf45), mRNA [NM_016045] | 2.65 |
| ANKRD11 | Homo sapiens ankyrin repeat domain 11 (ANKRD11), mRNA [NM_013275] | 2.66 |
| RKHD2 | Homo sapiens ring finger and KH domain containing 2 (RKHD2), mRNA [NM_016626] | 2.66 |
| BC036626 | Homo sapiens cDNA clone IMAGE:5288439. [BC036626] | 2.66 |
| C1orf201 | Homo sapiens chromosome 1 open reading frame 201 (C1orf201), mRNA [NM_178122] | 2.66 |
| CENTG3 | Homo sapiens centaurin, gamma 3 (CENTG3), transcript variant 1, mRNA [NM_031946] | 2.66 |
| PPP1R12A | Homo sapiens protein phosphatase 1, regulatory (inhibitor) subunit 12A (PPP1R12A), mRNA [NM_002480] | 2.67 |
| BC064492 | Homo sapiens cDNA clone IMAGE:5090094, partial cds. [BC064492] | 2.67 |
| STAT1 | Homo sapiens signal transducer and activator of transcription 1, 91kDa (STAT1), transcript variant alpha, mRNA [NM_007315] | 2.68 |
| A_23_P210285 | Unknown | 2.69 |
| OTUD4 | Homo sapiens OTU domain containing 4 (OTUD4), transcript variant 1, mRNA [NM_199324] | 2.69 |
| C20orf56 | Homo sapiens chromosome 20 open reading frame 56 (C20orf56) on chromosome 20 [NR_001558] | 2.69 |
| A_32_P93894 | Unknown | 2.69 |
| LRP11 | Homo sapiens low density lipoprotein receptor-related protein 11 (LRP11), mRNA [NM_032832] | 2.69 |
| THC2495785 | CN284574 17000531534200 GRN_EB Homo sapiens cDNA 5', mRNA sequence [CN284574] | 2.70 |
| PTMA | Homo sapiens prothymosin, alpha (gene sequence 28) (PTMA), mRNA [NM_002823] | 2.70 |
| ENST00000261569 | Microtubule-associated serine/threonine-protein kinase 4 (EC 2.7.11.1). [Source:Uniprot/SWISSPROT;Acc:O15021] [ENST00000261569] | 2.71 |
| CR603272 | full-length cDNA clone CS0DC013YI10 of Neuroblastoma Cot 25-normalized of Homo sapiens (human). [CR603272] | 2.71 |
| SIPA1L1 | Homo sapiens signal-induced proliferation-associated 1 like 1 (SIPA1L1), mRNA [NM_015556] | 2.71 |
| DEK | Homo sapiens DEK oncogene (DNA binding) (DEK), mRNA [NM_003472] | 2.71 |
| IL1RAP | Homo sapiens interleukin 1 receptor accessory protein (IL1RAP), transcript variant 1, mRNA [NM_002182] | 2.72 |
| SLC38A2 | Homo sapiens solute carrier family 38, member 2 (SLC38A2), mRNA [NM_018976] | 2.72 |
| ANKRD11 | Homo sapiens ankyrin repeat domain 11 (ANKRD11), mRNA [NM_013275] | 2.72 |
| APOC1 | Homo sapiens apolipoprotein C-I (APOC1), mRNA [NM_001645] | 2.73 |
| AMMECR1 | Homo sapiens Alport syndrome, mental retardation, midface hypoplasia and elliptocytosis chromosomal region, gene 1 (AMMECR1), transcript variant 1, mRNA [NM_015365] | 2.73 |
| STAT6 | Homo sapiens signal transducer and activator of transcription 6, interleukin-4 induced (STAT6), mRNA [NM_003153] | 2.74 |
| GYPB | Homo sapiens glycophorin B (MNS blood group) (GYPB), mRNA [NM_002100] | 2.74 |
| METAP2 | Homo sapiens methionyl aminopeptidase 2 (METAP2), mRNA [NM_006838] | 2.74 |
| EGR2 | Homo sapiens early growth response 2 (Krox-20 homolog, Drosophila) (EGR2), mRNA [NM_000399] | 2.75 |
| CENTG3 | Homo sapiens centaurin, gamma 3 (CENTG3), transcript variant 1, mRNA [NM_031946] | 2.75 |
| GAS6 | Homo sapiens growth arrest-specific 6 (GAS6), mRNA [NM_000820] | 2.75 |
| SMARCA2 | Homo sapiens SWI/SNF related, matrix associated, actin dependent regulator of chromatin, subfamily a, member 2 (SMARCA2), transcript variant 2, mRNA [NM_139045] | 2.75 |
| KCNH2 | Homo sapiens potassium voltage-gated channel, subfamily H (eag-related), member 2 (KCNH2), transcript variant 1, mRNA [NM_000238] | 2.75 |
| THC2696414 | Unknown | 2.75 |
| TMEM44 | Homo sapiens transmembrane protein 44 (TMEM44), transcript variant 1, mRNA [NM_138399] | 2.76 |
| PARP12 | Homo sapiens poly (ADP-ribose) polymerase family, member 12 (PARP12), mRNA [NM_022750] | 2.77 |
| GNPTAB | Homo sapiens N-acetylglucosamine-1-phosphate transferase, alpha and beta subunits (GNPTAB), mRNA [NM_024312] | 2.77 |
| ENST00000380344 | Beta-1,3-glucosyltransferase (EC 2.4.1.-) (Beta3Glc-T) (Beta-3- glycosyltransferase-like). [Source:Uniprot/SWISSPROT;Acc:Q6Y288] [ENST00000380344] | 2.77 |
| AHCTF1 | Homo sapiens AT hook containing transcription factor 1 (AHCTF1), mRNA [NM_015446] | 2.77 |
| TBL1XR1 | Homo sapiens transducin (beta)-like 1X-linked receptor 1 (TBL1XR1), mRNA [NM_024665] | 2.78 |
| A_24_P15502 | Unknown | 2.78 |
| SLC40A1 | Homo sapiens solute carrier family 40 (iron-regulated transporter), member 1 (SLC40A1), mRNA [NM_014585] | 2.78 |
| FAM76B | Homo sapiens family with sequence similarity 76, member B (FAM76B), mRNA [NM_144664] | 2.79 |
| HNRPU | Homo sapiens heterogeneous nuclear ribonucleoprotein U (scaffold attachment factor A) (HNRPU), transcript variant 1, mRNA [NM_031844] | 2.79 |
| ENST00000382990 | Unknown | 2.79 |
| THC2544148 | Q6TXI7_RAT (Q6TXI7) LRRGT00012, partial (5%) [THC2506462] | 2.79 |
| AK023412 | Homo sapiens cDNA FLJ13350 fis, clone OVARC1002143. [AK023412] | 2.80 |
| CTNNB1 | Homo sapiens catenin (cadherin-associated protein), beta 1, 88kDa (CTNNB1), mRNA [NM_001904] | 2.80 |
| PTMA | Homo sapiens prothymosin, alpha (gene sequence 28) (PTMA), mRNA [NM_002823] | 2.81 |
| NT5DC3 | Homo sapiens 5'-nucleotidase domain containing 3 (NT5DC3), transcript variant 2, mRNA [NM_016575] | 2.81 |
| THC2540396 | ALU8_HUMAN (P39195) Alu subfamily SX sequence contamination warning entry, partial (23%) [THC2540396] | 2.81 |
| GYPB | Homo sapiens glycophorin B (MNS blood group) (GYPB), mRNA [NM_002100] | 2.81 |
| IRF2BP2 | Homo sapiens interferon regulatory factor 2 binding protein 2 (IRF2BP2), transcript variant 1, mRNA [NM_182972] | 2.81 |
| CNKSR3 | Homo sapiens CNKSR family member 3 (CNKSR3), mRNA [NM_173515] | 2.82 |
| GOLPH2 | Homo sapiens golgi phosphoprotein 2 (GOLPH2), transcript variant 1, mRNA [NM_016548] | 2.82 |
| GBAS | Homo sapiens glioblastoma amplified sequence (GBAS), mRNA [NM_001483] | 2.83 |
| AW946823 | AW946823 RC2-ET0022-080500-012-b10 ET0022 Homo sapiens cDNA, mRNA sequence [AW946823] | 2.83 |
| JRK | Homo sapiens jerky homolog (mouse) (JRK), transcript variant 1, mRNA [NM_003724] | 2.84 |
| CDKN1C | Homo sapiens cyclin-dependent kinase inhibitor 1C (p57, Kip2) (CDKN1C), mRNA [NM_000076] | 2.84 |
| PDCD6IP | Homo sapiens programmed cell death 6 interacting protein (PDCD6IP), mRNA [NM_013374] | 2.85 |
| GAS2L1 | Homo sapiens growth arrest-specific 2 like 1 (GAS2L1), transcript variant 3, mRNA [NM_152237] | 2.85 |
| LOC541469 | Homo sapiens hypothetical LOC541469 protein (LOC541469), mRNA [NM_001013617] | 2.85 |
| PDE4DIP | Homo sapiens mRNA, similar to rat myomegalin, complete cds. [AB042555] | 2.85 |
| VAPA | Homo sapiens VAMP (vesicle-associated membrane protein)-associated protein A, 33kDa (VAPA), transcript variant 1, mRNA [NM_003574] | 2.86 |
| ENST00000341591 | PHD finger protein 10 (XAP135). [Source:Uniprot/SWISSPROT;Acc:Q8WUB8] [ENST00000341591] | 2.86 |
| C6orf148 | Homo sapiens chromosome 6 open reading frame 148 (C6orf148), mRNA [NM_030568] | 2.87 |
| MAP3K8 | Homo sapiens mitogen-activated protein kinase kinase kinase 8 (MAP3K8), mRNA [NM_005204] | 2.87 |
| SLC30A1 | Zinc transporter 1 (ZnT-1) (Solute carrier family 30 member 1). [Source:Uniprot/SWISSPROT;Acc:Q9Y6M5] [ENST00000367001] | 2.88 |
| ENST00000371189 | Nuclear factor 1 A-type (Nuclear factor 1/A) (NF1-A) (NFI-A) (NF-I/A) (CCAAT-box-binding transcription factor) (CTF) (TGGCA-binding protein). [Source:Uniprot/SWISSPROT;Acc:Q12857] [ENST00000371189] | 2.88 |
| FAM129A | Homo sapiens family with sequence similarity 129, member A (FAM129A), transcript variant 2, mRNA [NM_052966] | 2.88 |
| THC2634957 | Q3S279_9STRA (Q3S279) NADH dehydrogenase subunit 9, partial (7%) [THC2730335] | 2.89 |
| EFHD1 | Homo sapiens EF-hand domain family, member D1 (EFHD1), mRNA [NM_025202] | 2.89 |
| EMID1 | Homo sapiens EMI domain containing 1 (EMID1), mRNA [NM_133455] | 2.90 |
| ZNF84 | Homo sapiens zinc finger protein 84 (ZNF84), mRNA [NM_003428] | 2.90 |
| CUGBP2 | Homo sapiens CUG triplet repeat, RNA binding protein 2 (CUGBP2), transcript variant 3, mRNA [NM_001025077] | 2.91 |
| PPP1R12A | Homo sapiens protein phosphatase 1, regulatory (inhibitor) subunit 12A (PPP1R12A), mRNA [NM_002480] | 2.92 |
| CKMT1A | Homo sapiens creatine kinase, mitochondrial 1A (CKMT1A), nuclear gene encoding mitochondrial protein, mRNA [NM_001015001] | 2.94 |
| CENTB2 | Homo sapiens centaurin, beta 2 (CENTB2), mRNA [NM_012287] | 2.95 |
| RRAD | Homo sapiens Ras-related associated with diabetes (RRAD), mRNA [NM_004165] | 2.95 |
| LEMD3 | Homo sapiens LEM domain containing 3 (LEMD3), mRNA [NM_014319] | 2.96 |
| ADNP | Homo sapiens activity-dependent neuroprotector (ADNP), transcript variant 1, mRNA [NM_015339] | 2.97 |
| ATG5 | Homo sapiens ATG5 autophagy related 5 homolog (S. cerevisiae) (ATG5), mRNA [NM_004849] | 2.97 |
| PTGDS | Homo sapiens prostaglandin D2 synthase 21kDa (brain) (PTGDS), mRNA [NM_000954] | 2.97 |
| RP5-1022P6.2 | Putative glycerophosphodiester phosphodiesterase 5 (EC 3.1.-.-). [Source:Uniprot/SWISSPROT;Acc:Q9NPB8] [ENST00000379019] | 2.98 |
| BCAP29 | Homo sapiens B-cell receptor-associated protein 29 (BCAP29), transcript variant 4, mRNA [NM_001008407] | 2.98 |
| PSD4 | Homo sapiens pleckstrin and Sec7 domain containing 4 (PSD4), mRNA [NM_012455] | 2.99 |
| AK124936 | Homo sapiens cDNA FLJ42946 fis, clone BRSTN2005721. [AK124936] | 3.00 |
| APOC1 | Homo sapiens apolipoprotein C-I (APOC1), mRNA [NM_001645] | 3.01 |
| HMGB2 | Homo sapiens high-mobility group box 2 (HMGB2), mRNA [NM_002129] | 3.01 |
| TTC13 | Homo sapiens tetratricopeptide repeat domain 13 (TTC13), mRNA [NM_024525] | 3.02 |
| PPIA | Homo sapiens peptidylprolyl isomerase A (cyclophilin A) (PPIA), mRNA [NM_021130] | 3.04 |
| CR612226 | full-length cDNA clone CS0DF019YP13 of Fetal brain of Homo sapiens (human). [CR612226] | 3.04 |
| KCNMB4 | Homo sapiens potassium large conductance calcium-activated channel, subfamily M, beta member 4 (KCNMB4), mRNA [NM_014505] | 3.04 |
| COL6A1 | Homo sapiens collagen, type VI, alpha 1 (COL6A1), mRNA [NM_001848] | 3.07 |
| C6orf111 | Splicing factor, arginine/serine-rich 130 (Serine-arginine-rich- splicing regulatory protein 130) (SRrp130) (SR-rich protein) (SR- related protein). [Source:Uniprot/SWISSPROT;Acc:Q8TF01] [ENST00000369239] | 3.08 |
| CXXC6 | CXXC-type zinc finger protein 6 (Leukemia-associated protein with a CXXC domain). [Source:Uniprot/SWISSPROT;Acc:Q8NFU7] [ENST00000373644] | 3.08 |
| THC2660361 | Unknown | 3.09 |
| TSC22D1 | Homo sapiens TSC22 domain family, member 1 (TSC22D1), transcript variant 1, mRNA [NM_183422] | 3.09 |
| SH3KBP1 | Homo sapiens SH3-domain kinase binding protein 1 (SH3KBP1), transcript variant 2, mRNA [NM_001024666] | 3.09 |
| HRC | Homo sapiens histidine rich calcium binding protein (HRC), mRNA [NM_002152] | 3.09 |
| A_24_P290087 | Unknown | 3.10 |
| TFDP1 | Homo sapiens transcription factor Dp-1 (TFDP1), mRNA [NM_007111] | 3.11 |
| DIDO1 | Homo sapiens death inducer-obliterator 1 (DIDO1), transcript variant 3, mRNA [NM_080797] | 3.13 |
| GNPTAB | Homo sapiens N-acetylglucosamine-1-phosphate transferase, alpha and beta subunits (GNPTAB), mRNA [NM_024312] | 3.14 |
| P18SRP | Homo sapiens P18SRP protein (P18SRP), mRNA [NM_173829] | 3.14 |
| TMEM48 | Homo sapiens cDNA FLJ34120 fis, clone FCBBF3009541. [AK091439] | 3.14 |
| JDP2 | Homo sapiens jun dimerization protein 2 (JDP2), mRNA [NM_130469] | 3.14 |
| LPHN1 | Homo sapiens latrophilin 1 (LPHN1), transcript variant 1, mRNA [NM_001008701] | 3.15 |
| PLA2G3 | Homo sapiens phospholipase A2, group III (PLA2G3), mRNA [NM_015715] | 3.15 |
| PHTF2 | Homo sapiens putative homeodomain transcription factor 2 (PHTF2), mRNA [NM_020432] | 3.16 |
| CR612518 | full-length cDNA clone CS0DF004YF08 of Fetal brain of Homo sapiens (human). [CR612518] | 3.16 |
| ERMAP | Homo sapiens erythroblast membrane-associated protein (Scianna blood group) (ERMAP), transcript variant 1, mRNA [NM_001017922] | 3.16 |
| THC2692669 | ALU1_HUMAN (P39188) Alu subfamily J sequence contamination warning entry, partial (10%) [THC2692669] | 3.17 |
| CHST2 | Homo sapiens carbohydrate (N-acetylglucosamine-6-O) sulfotransferase 2 (CHST2), mRNA [NM_004267] | 3.17 |
| CR608907 | full-length cDNA clone CS0DM002YA18 of Fetal liver of Homo sapiens (human). [CR608907] | 3.17 |
| ARID2 | Homo sapiens AT rich interactive domain 2 (ARID, RFX-like) (ARID2), mRNA [NM_152641] | 3.18 |
| TFRC | Homo sapiens transferrin receptor (p90, CD71) (TFRC), mRNA [NM_003234] | 3.21 |
| THC2730823 | O19057_PONPY (O19057) Fertilin alpha protein, partial (19%) [THC2730823] | 3.21 |
| SEZ6L2 | Homo sapiens seizure related 6 homolog (mouse)-like 2 (SEZ6L2), transcript variant 2, mRNA [NM_201575] | 3.23 |
| FBXO45 | Homo sapiens cDNA: FLJ22044 fis, clone HEP09141. [AK025697] | 3.24 |
| MGC102966 | Homo sapiens similar to Keratin, type I cytoskeletal 16 (Cytokeratin-16) (CK-16) (Keratin-16) (K16), mRNA (cDNA clone MGC:102966 IMAGE:4752428), complete cds. [BC110641] | 3.25 |
| TXNDC13 | Thioredoxin domain-containing protein 13 precursor. [Source:Uniprot/SWISSPROT;Acc:Q9H1E5] [ENST00000246024] | 3.26 |
| AL831999 | Homo sapiens mRNA; cDNA DKFZp451K063 (from clone DKFZp451K063). [AL831999] | 3.26 |
| TBC1D10C | Homo sapiens TBC1 domain family, member 10C (TBC1D10C), mRNA [NM_198517] | 3.26 |
| PKLR | Homo sapiens pyruvate kinase, liver and RBC (PKLR), nuclear gene encoding mitochondrial protein, transcript variant 1, mRNA [NM_000298] | 3.28 |
| C6orf204 | C6orf204 protein (Fragment). [Source:Uniprot/SPTREMBL;Acc:Q3ZCQ5] [ENST00000368491] | 3.28 |
| GLCCI1 | Homo sapiens glucocorticoid induced transcript 1 (GLCCI1), mRNA [NM_138426] | 3.30 |
| BI771054 | 603059613F1 NIH_MGC_122 Homo sapiens cDNA clone IMAGE:5209035 5', mRNA sequence [BI771054] | 3.35 |
| CD164 | Homo sapiens CD164 molecule, sialomucin (CD164), mRNA [NM_006016] | 3.35 |
| LYZ | Homo sapiens lysozyme (renal amyloidosis) (LYZ), mRNA [NM_000239] | 3.35 |
| ATP13A3 | Probable cation-transporting ATPase 13A3 (EC 3.6.3.-) (ATPase family homolog up-regulated in senescence cells 1). [Source:Uniprot/SWISSPROT;Acc:Q9H7F0] [ENST00000256031] | 3.37 |
| THC2525827 | Unknown | 3.37 |
| AL080082 | Homo sapiens mRNA; cDNA DKFZp564G1162 (from clone DKFZp564G1162). [AL080082] | 3.39 |
| SOX4 | Homo sapiens SRY (sex determining region Y)-box 4 (SOX4), mRNA [NM_003107] | 3.40 |
| CENPF | Homo sapiens centromere protein F, 350/400ka (mitosin) (CENPF), mRNA [NM_016343] | 3.41 |
| THC2783023 | Q8IUM9_HUMAN (Q8IUM9) ACSL3 protein, complete [THC2467888] | 3.41 |
| HDDC2 | Homo sapiens HD domain containing 2 (HDDC2), mRNA [NM_016063] | 3.43 |
| SYNCRIP | Homo sapiens synaptotagmin binding, cytoplasmic RNA interacting protein, mRNA (cDNA clone MGC:45213 IMAGE:5495201), complete cds. [BC032643] | 3.43 |
| OSBPL8 | Homo sapiens oxysterol binding protein-like 8 (OSBPL8), transcript variant 1, mRNA [NM_020841] | 3.43 |
| MLXIP | Homo sapiens MLX interacting protein (MLXIP), mRNA [NM_014938] | 3.47 |
| SLC16A5 | Homo sapiens solute carrier family 16, member 5 (monocarboxylic acid transporter 6) (SLC16A5), mRNA [NM_004695] | 3.48 |
| SACS | Homo sapiens spastic ataxia of Charlevoix-Saguenay (sacsin) (SACS), mRNA [NM_014363] | 3.50 |
| Sep-05 | Homo sapiens septin 5 (SEPT5), mRNA [NM_002688] | 3.51 |
| A_24_P325533 | Unknown | 3.53 |
| SEZ6L2 | Homo sapiens seizure related 6 homolog (mouse)-like 2 (SEZ6L2), transcript variant 2, mRNA [NM_201575] | 3.57 |
| NR4A1 | Homo sapiens nuclear receptor subfamily 4, group A, member 1 (NR4A1), transcript variant 1, mRNA [NM_002135] | 3.59 |
| GOLPH2 | Homo sapiens golgi phosphoprotein 2 (GOLPH2), transcript variant 1, mRNA [NM_016548] | 3.59 |
| AKAP12 | Homo sapiens A kinase (PRKA) anchor protein (gravin) 12 (AKAP12), transcript variant 2, mRNA [NM_144497] | 3.60 |
| ATP8B3 | Homo sapiens ATPase, Class I, type 8B, member 3 (ATP8B3), mRNA [NM_138813] | 3.62 |
| LOC340508 | Homo sapiens hypothetical protein LOC340508 (LOC340508) on chromosome 9 [NR_002942] | 3.63 |
| SMARCA1 | Homo sapiens SWI/SNF related, matrix associated, actin dependent regulator of chromatin, subfamily a, member 1 (SMARCA1), transcript variant 1, mRNA [NM_003069] | 3.63 |
| EGFLAM | Homo sapiens EGF-like, fibronectin type III and laminin G domains (EGFLAM), transcript variant 1, mRNA [NM_152403] | 3.68 |
| LRCH2 | Homo sapiens leucine-rich repeats and calponin homology (CH) domain containing 2 (LRCH2), mRNA [NM_020871] | 3.71 |
| SLC4A11 | Homo sapiens solute carrier family 4, sodium bicarbonate transporter-like, member 11 (SLC4A11), mRNA [NM_032034] | 3.73 |
| SLC10A4 | Homo sapiens solute carrier family 10 (sodium/bile acid cotransporter family), member 4 (SLC10A4), mRNA [NM_152679] | 3.73 |
| RNASE1 | Homo sapiens ribonuclease, RNase A family, 1 (pancreatic) (RNASE1), transcript variant 3, mRNA [NM_198232] | 3.75 |
| SOX4 | Homo sapiens SRY (sex determining region Y)-box 4 (SOX4), mRNA [NM_003107] | 3.77 |
| ENST00000310773 | Probable cation-transporting ATPase 13A3 (EC 3.6.3.-) (ATPase family homolog up-regulated in senescence cells 1). [Source:Uniprot/SWISSPROT;Acc:Q9H7F0] [ENST00000310773] | 3.80 |
| THC2679226 | Q4T539_TETNG (Q4T539) Chromosome undetermined SCAF9438, whole genome shotgun sequence. (Fragment), partial (4%) [THC2679226] | 3.81 |
| CTNNB1 | Homo sapiens catenin (cadherin-associated protein), beta 1, 88kDa (CTNNB1), mRNA [NM_001904] | 3.82 |
| PTPN7 | Homo sapiens protein tyrosine phosphatase, non-receptor type 7 (PTPN7), transcript variant 2, mRNA [NM_080588] | 3.84 |
| FGFR1OP | C-C chemokine receptor type 6 (C-C CKR-6) (CC-CKR-6) (CCR-6) (LARC receptor) (GPR-CY4) (GPRCY4) (Chemokine receptor-like 3) (CKR-L3) (DRY6) (G-protein coupled receptor 29) (CD196 antigen). [Source:Uniprot/SWISSPROT;Acc:P51684] [ENST00000366847] | 3.93 |
| ENST00000312943 | Docking protein 3 (Downstream of tyrosine kinase 3). [Source:Uniprot/SWISSPROT;Acc:Q7L591] [ENST00000312943] | 3.95 |
| PRKAR2B | Homo sapiens protein kinase, cAMP-dependent, regulatory, type II, beta (PRKAR2B), mRNA [NM_002736] | 4.02 |
| EED | Homo sapiens embryonic ectoderm development (EED), transcript variant 2, mRNA [NM_152991] | 4.03 |
| C16orf7 | Homo sapiens chromosome 16 open reading frame 7 (C16orf7), mRNA [NM_004913] | 4.08 |
| CAB39L | Homo sapiens calcium binding protein 39-like (CAB39L), transcript variant 1, mRNA [NM_030925] | 4.09 |
| NCR1 | Homo sapiens natural cytotoxicity triggering receptor 1, mRNA (cDNA clone MGC:65100 IMAGE:5218848), complete cds. [BC064806] | 4.12 |
| NR3C1 | Homo sapiens nuclear receptor subfamily 3, group C, member 1 (glucocorticoid receptor) (NR3C1), transcript variant 1, mRNA [NM_001018077] | 4.12 |
| BC043173 | Homo sapiens cDNA clone IMAGE:5287121. [BC043173] | 4.13 |
| MCM7 | Homo sapiens MCM7 minichromosome maintenance deficient 7 (S. cerevisiae) (MCM7), transcript variant 2, mRNA [NM_182776] | 4.13 |
| ACSL3 | Homo sapiens acyl-CoA synthetase long-chain family member 3 (ACSL3), transcript variant 1, mRNA [NM_004457] | 4.21 |
| GLCCI1 | Homo sapiens glucocorticoid induced transcript 1 (GLCCI1), mRNA [NM_138426] | 4.24 |
| SLC25A40 | Homo sapiens mRNA; cDNA DKFZp564D0472 (from clone DKFZp564D0472) [AL110179] | 4.26 |
| LOC643201 | Homo sapiens cDNA clone IMAGE:5171181. [BC034407] | 4.35 |
| NRTN | Homo sapiens neurturin (NRTN), mRNA [NM_004558] | 4.36 |
| RELB | Homo sapiens v-rel reticuloendotheliosis viral oncogene homolog B, nuclear factor of kappa light polypeptide gene enhancer in B-cells 3 (avian) (RELB), mRNA [NM_006509] | 4.37 |
| FKBP11 | Homo sapiens FK506 binding protein 11, 19 kDa (FKBP11), mRNA [NM_016594] | 4.38 |
| HLTF | Homo sapiens helicase-like transcription factor (HLTF), transcript variant 1, mRNA [NM_003071] | 4.41 |
| DOCK8 | Homo sapiens dedicator of cytokinesis 8 (DOCK8), mRNA [NM_203447] | 4.42 |
| RRP22 | Homo sapiens RAS-related on chromosome 22 (RRP22), transcript variant 2, mRNA [NM_001007279] | 4.48 |
| U09197 | Human 5.5 kb mRNA upregulated in retinoic acid treated HL-60 neutrophilic cells. [U09197] | 4.49 |
| RKHD3 | Homo sapiens ring finger and KH domain containing 3 (RKHD3), mRNA [NM_032246] | 4.53 |
| TCEAL4 | Homo sapiens transcription elongation factor A (SII)-like 4 (TCEAL4), transcript variant 1, mRNA [NM_024863] | 4.53 |
| WWC3 | Homo sapiens WWC family member 3 (WWC3), mRNA [NM_015691] | 4.57 |
| LIMD2 | Homo sapiens LIM domain containing 2 (LIMD2), mRNA [NM_030576] | 4.65 |
| LIFR | Homo sapiens leukemia inhibitory factor receptor alpha (LIFR), mRNA [NM_002310] | 4.66 |
| RKHD3 | Homo sapiens ring finger and KH domain containing 3 (RKHD3), mRNA [NM_032246] | 4.70 |
| HSD17B8 | Homo sapiens hydroxysteroid (17-beta) dehydrogenase 8 (HSD17B8), mRNA [NM_014234] | 4.74 |
| S100A4 | Homo sapiens S100 calcium binding protein A4 (S100A4), transcript variant 1, mRNA [NM_002961] | 4.79 |
| C18orf2 | Homo sapiens putative C18orf2 variant 1 mRNA, complete cds, alternatively spliced. [AF295726] | 4.81 |
| DRAM | Homo sapiens damage-regulated autophagy modulator (DRAM), mRNA [NM_018370] | 4.83 |
| SELENBP1 | Homo sapiens selenium binding protein 1 (SELENBP1), mRNA [NM_003944] | 4.92 |
| ARL15 | Homo sapiens ADP-ribosylation factor-like 15 (ARL15), mRNA [NM_019087] | 4.92 |
| PRSSL1 | Homo sapiens protease, serine-like 1 (PRSSL1), mRNA [NM_214710] | 4.93 |
| AF289562 | Homo sapiens clone pp6337 unknown mRNA. [AF289562] | 5.00 |
| GUSBP1 | Homo sapiens glucuronidase, beta pseudogene 1 (GUSBP1), mRNA [NM_207331] | 5.03 |
| DRAM | Homo sapiens damage-regulated autophagy modulator (DRAM), mRNA [NM_018370] | 5.08 |
| EREG | Homo sapiens epiregulin (EREG), mRNA [NM_001432] | 5.10 |
| AEBP1 | Homo sapiens AE binding protein 1 (AEBP1), mRNA [NM_001129] | 5.24 |
| MARCKS | Homo sapiens myristoylated alanine-rich protein kinase C substrate (MARCKS), mRNA [NM_002356] | 5.27 |
| THC2540396 | ALU8_HUMAN (P39195) Alu subfamily SX sequence contamination warning entry, partial (23%) [THC2540396] | 5.28 |
| RHOH | Homo sapiens ras homolog gene family, member H (RHOH), mRNA [NM_004310] | 5.44 |
| LCP2 | Homo sapiens lymphocyte cytosolic protein 2 (SH2 domain containing leukocyte protein of 76kDa) (LCP2), mRNA [NM_005565] | 5.45 |
| NR3C1 | Homo sapiens nuclear receptor subfamily 3, group C, member 1 (glucocorticoid receptor) (NR3C1), transcript variant 1, mRNA [NM_001018077] | 5.50 |
| ARHGAP15 | Homo sapiens Rho GTPase activating protein 15 (ARHGAP15), mRNA [NM_018460] | 5.56 |
| ENST00000339867 | similar to Interferon-induced transmembrane protein 3 (Interferon-inducible protein 1-8U) (LOC650205), mRNA [Source:RefSeq_dna;Acc:XR_018421] [ENST00000339867] | 5.57 |
| CD24 | Homo sapiens CD24 signal transducer mRNA, complete cds and 3' region. [L33930] | 5.60 |
| LOC728555 | Homo sapiens cDNA FLJ40901 fis, clone UTERU2003704. [AK098220] | 5.64 |
| LOC643201 | Homo sapiens cDNA clone IMAGE:5268504. [BC052945] | 5.65 |
| ANKRD55 | Homo sapiens ankyrin repeat domain 55 (ANKRD55), transcript variant 1, mRNA [NM_024669] | 5.76 |
| RPP25 | Homo sapiens ribonuclease P 25kDa subunit (RPP25), mRNA [NM_017793] | 5.77 |
| APOB48R | Homo sapiens apolipoprotein B48 receptor (APOB48R), mRNA [NM_018690] | 5.81 |
| TMEM65 | Homo sapiens transmembrane protein 65 (TMEM65), mRNA [NM_194291] | 5.89 |
| LAMC3 | Homo sapiens laminin, gamma 3 (LAMC3), mRNA [NM_006059] | 5.96 |
| SLC43A3 | Homo sapiens solute carrier family 43, member 3 (SLC43A3), mRNA [NM_199329] | 5.96 |
| ROBO3 | Homo sapiens roundabout, axon guidance receptor, homolog 3 (Drosophila) (ROBO3), mRNA [NM_022370] | 6.00 |
| AK125361 | Homo sapiens cDNA FLJ43371 fis, clone NTONG2005969. [AK125361] | 6.03 |
| CARD9 | Homo sapiens caspase recruitment domain family, member 9 (CARD9), mRNA [NM_052813] | 6.04 |
| AK021751 | Homo sapiens cDNA FLJ11689 fis, clone HEMBA1004977. [AK021751] | 6.29 |
| FZD2 | Homo sapiens frizzled homolog 2 (Drosophila) (FZD2), mRNA [NM_001466] | 6.72 |
| TRDN | Homo sapiens triadin (TRDN), mRNA [NM_006073] | 6.98 |
| SLC35F3 | Homo sapiens solute carrier family 35, member F3 (SLC35F3), mRNA [NM_173508] | 7.16 |
| BAIAP2L2 | Homo sapiens BAI1-associated protein 2-like 2 (BAIAP2L2), mRNA [NM_025045] | 7.19 |
| TNS4 | Homo sapiens tensin 4 (TNS4), mRNA [NM_032865] | 7.40 |
| IGFBP4 | Homo sapiens insulin-like growth factor binding protein 4 (IGFBP4), mRNA [NM_001552] | 7.49 |
| TAP1 | Homo sapiens transporter 1, ATP-binding cassette, sub-family B (MDR/TAP) (TAP1), mRNA [NM_000593] | 7.66 |
| VAV1 | Homo sapiens vav 1 oncogene (VAV1), mRNA [NM_005428] | 7.89 |
| IFITM2 | Homo sapiens interferon induced transmembrane protein 2 (1-8D) (IFITM2), mRNA [NM_006435] | 8.19 |
| CITED4 | Homo sapiens Cbp/p300-interacting transactivator, with Glu/Asp-rich carboxy-terminal domain, 4 (CITED4), mRNA [NM_133467] | 8.42 |
| LGALS3 | Homo sapiens lectin, galactoside-binding, soluble, 3 (galectin 3) (LGALS3), transcript variant 1, mRNA [NM_002306] | 8.65 |
| INHBA | Homo sapiens inhibin, beta A (activin A, activin AB alpha polypeptide) (INHBA), mRNA [NM_002192] | 8.86 |
| COL4A2 | Homo sapiens collagen, type IV, alpha 2 (COL4A2), mRNA [NM_001846] | 9.14 |
| BC014265 | Homo sapiens cDNA clone IMAGE:4639114, partial cds. [BC014265] | 9.36 |
| TRIM26 | Homo sapiens tripartite motif-containing 26 (TRIM26), mRNA [NM_003449] | 9.57 |
| EBI3 | Homo sapiens Epstein-Barr virus induced gene 3 (EBI3), mRNA [NM_005755] | 9.72 |
| KCNG1 | Homo sapiens potassium voltage-gated channel, subfamily G, member 1 (KCNG1), transcript variant 1, mRNA [NM_002237] | 10.05 |
| EPB41L2 | Homo sapiens erythrocyte membrane protein band 4.1-like 2 (EPB41L2), mRNA [NM_001431] | 10.44 |
| DLK1 | Homo sapiens delta-like 1 homolog (Drosophila) (DLK1), transcript variant 1, mRNA [NM_003836] | 10.56 |
| CD53 | Homo sapiens CD53 molecule (CD53), transcript variant 1, mRNA [NM_001040033] | 10.59 |
| LIFR | Homo sapiens leukemia inhibitory factor receptor alpha (LIFR), mRNA [NM_002310] | 10.98 |
| PLP2 | Homo sapiens proteolipid protein 2 (colonic epithelium-enriched) (PLP2), mRNA [NM_002668] | 11.05 |
| LRMP | Homo sapiens lymphoid-restricted membrane protein (LRMP), mRNA [NM_006152] | 11.06 |
| LAPTM5 | Homo sapiens lysosomal associated multispanning membrane protein 5 (LAPTM5), mRNA [NM_006762] | 11.28 |
| A_23_P40225 | Unknown | 12.05 |
| THC2582296 | 1EA8_A Chain A, Apolipoprotein E3 22kd Fragment Lys146glu Mutant. {Homo sapiens} (exp=-1; wgp=-1; cg=-1), partial (64%) [THC2582296] | 12.48 |
| ZBTB38 | Homo sapiens cDNA clone IMAGE:6168734. [BC072415] | 12.51 |
| KYNU | Homo sapiens kynureninase (L-kynurenine hydrolase) (KYNU), transcript variant 1, mRNA [NM_003937] | 12.73 |
| GPR160 | Homo sapiens G protein-coupled receptor 160 (GPR160), mRNA [NM_014373] | 13.09 |
| S100A6 | Homo sapiens S100 calcium binding protein A6 (S100A6), mRNA [NM_014624] | 13.20 |
| SLC43A3 | Homo sapiens solute carrier family 43, member 3 (SLC43A3), mRNA [NM_199329] | 13.26 |
| PYCARD | Homo sapiens PYD and CARD domain containing (PYCARD), transcript variant 1, mRNA [NM_013258] | 13.30 |
| APOE | Homo sapiens apolipoprotein E (APOE), mRNA [NM_000041] | 13.94 |
| BG216229 | BG216229 RST35803 Athersys RAGE Library Homo sapiens cDNA, mRNA sequence [BG216229] | 14.37 |
| IL18RAP | Homo sapiens interleukin 18 receptor accessory protein (IL18RAP), mRNA [NM_003853] | 14.53 |
| CHES1 | Homo sapiens checkpoint suppressor 1 (CHES1), mRNA [NM_005197] | 14.84 |
| CCND1 | Homo sapiens cyclin D1 (CCND1), mRNA [NM_053056] | 14.98 |
| LAPTM5 | Homo sapiens lysosomal associated multispanning membrane protein 5 (LAPTM5), mRNA [NM_006762] | 15.20 |
| CCND1 | Homo sapiens cyclin D1 (CCND1), mRNA [NM_053056] | 16.04 |
| PEPP-2 | Homo sapiens PEPP subfamily gene 2 (PEPP-2), mRNA [NM_032498] | 16.30 |
| ECHDC3 | Homo sapiens enoyl Coenzyme A hydratase domain containing 3 (ECHDC3), mRNA [NM_024693] | 17.03 |
| ARHGDIB | Homo sapiens Rho GDP dissociation inhibitor (GDI) beta (ARHGDIB), mRNA [NM_001175] | 17.04 |
| A_32_P125808 | Unknown | 17.56 |
| ECHDC3 | Homo sapiens enoyl Coenzyme A hydratase domain containing 3 (ECHDC3), mRNA [NM_024693] | 18.50 |
| CCDC83 | Homo sapiens coiled-coil domain containing 83 (CCDC83), mRNA [NM_173556] | 20.81 |
| MGC16291 | Homo sapiens hypothetical protein MGC16291, mRNA (cDNA clone MGC:16291 IMAGE:3834089), complete cds. [BC007394] | 21.23 |
| SLC16A9 | Homo sapiens solute carrier family 16, member 9 (monocarboxylic acid transporter 9) (SLC16A9), mRNA [NM_194298] | 21.53 |
| IL18R1 | Homo sapiens interleukin 18 receptor 1 (IL18R1), mRNA [NM_003855] | 22.53 |
| AKAP7 | Homo sapiens A kinase (PRKA) anchor protein 7 (AKAP7), transcript variant gamma, mRNA [NM_016377] | 22.84 |
| NLRP2 | Homo sapiens NLR family, pyrin domain containing 2 (NLRP2), mRNA [NM_017852] | 23.74 |
| IRX4 | Homo sapiens iroquois homeobox protein 4 (IRX4), mRNA [NM_016358] | 24.16 |
| NMI | Homo sapiens N-myc (and STAT) interactor (NMI), mRNA [NM_004688] | 24.23 |
| BIRC3 | Homo sapiens baculoviral IAP repeat-containing 3 (BIRC3), transcript variant 1, mRNA [NM_001165] | 24.26 |
| CTSS | Homo sapiens cathepsin S (CTSS), mRNA [NM_004079] | 24.52 |
| ZNF31 | Homo sapiens zinc finger protein 31 (ZNF31), mRNA [NM_145238] | 27.02 |
| NTN1 | Homo sapiens cDNA clone IMAGE:30530513. [BC092429] | 27.83 |
| SOHLH2 | Homo sapiens spermatogenesis and oogenesis specific basic helix-loop-helix 2 (SOHLH2), mRNA [NM_017826] | 28.99 |
| BRDG1 | Homo sapiens BCR downstream signaling 1 (BRDG1), mRNA [NM_012108] | 30.02 |
| MDK | Homo sapiens midkine (neurite growth-promoting factor 2) (MDK), transcript variant 1, mRNA [NM_001012334] | 30.69 |
| TNFAIP2 | Homo sapiens tumor necrosis factor, alpha-induced protein 2 (TNFAIP2), mRNA [NM_006291] | 31.04 |
| NLRX1 | Homo sapiens NLR family member X1 (NLRX1), transcript variant 2, mRNA [NM_170722] | 36.85 |
| PLAGL1 | Homo sapiens pleiomorphic adenoma gene-like 1 (PLAGL1), transcript variant 2, mRNA [NM_006718] | 37.46 |
| MGC16291 | Homo sapiens hypothetical protein MGC16291, mRNA (cDNA clone MGC:16291 IMAGE:3834089), complete cds. [BC007394] | 37.72 |
| LCP1 | Homo sapiens lymphocyte cytosolic protein 1 (L-plastin) (LCP1), mRNA [NM_002298] | 37.77 |
| LTB | Homo sapiens lymphotoxin beta (TNF superfamily, member 3) (LTB), transcript variant 1, mRNA [NM_002341] | 39.35 |
| SYK | Homo sapiens spleen tyrosine kinase (SYK), mRNA [NM_003177] | 40.05 |
| INPP5D | Homo sapiens inositol polyphosphate-5-phosphatase, 145kDa (INPP5D), transcript variant 1, mRNA [NM_001017915] | 41.08 |
| CDH18 | Homo sapiens cadherin 18, type 2 (CDH18), mRNA [NM_004934] | 46.97 |
| DNAJA4 | Homo sapiens DnaJ (Hsp40) homolog, subfamily A, member 4 (DNAJA4), mRNA [NM_018602] | 54.48 |
| PKIB | Homo sapiens protein kinase (cAMP-dependent, catalytic) inhibitor beta (PKIB), transcript variant 1, mRNA [NM_181795] | 55.10 |
| MXRA8 | Homo sapiens matrix-remodelling associated 8 (MXRA8), mRNA [NM_032348] | 55.95 |
| AK026351 | Homo sapiens cDNA: FLJ22698 fis, clone HSI12044. [AK026351] | 56.02 |
| MAGEH1 | Homo sapiens melanoma antigen family H, 1 (MAGEH1), mRNA [NM_014061] | 59.52 |
| C5orf13 | Homo sapiens chromosome 5 open reading frame 13 (C5orf13), mRNA [NM_004772] | 61.16 |
| GPR125 | Homo sapiens G protein-coupled receptor 125 (GPR125), mRNA [NM_145290] | 111.51 |
| CTCFL | Homo sapiens CCCTC-binding factor (zinc finger protein)-like (CTCFL), mRNA [NM_080618] | 113.08 |
| PEPP-2 | Paired-like homeobox protein PEPP-2 (Testis homeobox gene 1). [Source:Uniprot/SWISSPROT;Acc:Q9BQY4] [ENST00000371388] | 303.41 |
| PEPP-2 | Homo sapiens PEPP subfamily gene 2 (PEPP-2), mRNA [NM_032498] | 607.81 |
