# Supplementary material for: Development and characterization of an in vitro model of colorectal adenocarcinoma with MDR phenotype
Source: Cancer Med. 2016 Mar 25;5(6):1279–91. doi: 10.1002/cam4.694 (PMC4924386; doi:10.1002/cam4.694)
Supplement: Supplementary file 3 — Table S2. List of pathways significantly enriched by GO‐Elite analysis. [file CAM4-5-1279-s003.doc]

**Table 2 supplementary material**: List of pathways significantly enriched by GO-Elite analysis. The different columns show the number of genes differentially expressed comparing HCT-8/R cells to HCT-8 parental cells, the number of genes analyzed, the z-score and the p-value.

| **Pathways down-regulated** | | | | | |
| --- | --- | --- | --- | --- | --- |
| **MAPP Name** | **Number Changed** | **Number Measured** | **Z Score** | **PermuteP** | **gene symbols** |
| Cell cycle:WP179 | 34 | 101 | 3.06 | 0.00 | ARF1|BUB1|CCNB2|CCND2|CCNE2|CCNH|CDK2|CDK4|DBF4|E2F3|E2F4|E2F5|E2F6|ESPL1|HDAC2|HDAC4|HDAC6|HDAC7|HDAC8|MCM4|MCM7|MCM8|MCM9|MDM2|ORC4|PTTG1|PTTG2|PTTG3P|RB1|SKP2|SMAD4|TFDP1|YWHAG|YWHAQ |
| p38 MAPK Signaling Pathway:WP400 | 12 | 30 | 2.50 | 0.01 | ATF2|CDC42|HSPB1|MAP3K5|MAPKAPK5|MAX|MEF2D|MYC|PLA2G4A|STAT1|TGFBR1|TRADD |
| TGF beta Signaling Pathway:WP366 | 34 | 111 | 2.42 | 0.01 | ATF2|BCAR1|CCNB2|CCND1|CDC42|DAB2|E2F4|E2F5|EID2|FOS|ITCH|ITGB1|JUNB|KLF6|MAPK8|MYC|NEDD4L|NUP153|PAK2|PJA1|PRKAR2A|SKP1|SMAD4|SNW1|SP1|TAB1|TERT|TFDP1|TGFBR1|TRAF6|TRAP1|UBE2I|UCHL5|ZFYVE16 |
| IL-4 signaling pathway:WP395 | 16 | 45 | 2.33 | 0.02 | ATF2|FES|FOS|IL4R|INPP5D|IRS1|NFKB1|NFKBIA|PIK3CA|PIK3CD|PTPN11|RELA|SOCS1|SOCS3|STAT1|STAT6 |
| Kit receptor signaling pathway:WP304 | 18 | 53 | 2.25 | 0.02 | BTK|CRK|DOK1|FOS|GRB10|INPP5D|JUNB|KIT|MAPK8|MATK|MITF|PRKCB|PTPN11|SOCS1|SOCS6|STAT1|STAT5A|VAV1 |
| Wnt Signaling Pathway and Pluripotency:WP399 | 29 | 98 | 2.01 | 0.04 | CCND1|CCND2|CD44|CTBP2|CTNNB1|DVL3|ESRRB|FZD1|FZD2|FZD4|FZD5|LEF1|MYC|NFYA|NLK|PAFAH1B1|PLAU|PPARD|PPM1J|PPP2R1B|PPP2R3B|PPP2R5C|PPP2R5E|PRKCB|PRKCE|PRKCZ|TCF7L2|WNT10B|WNT6 |
| **Pathways up-regulated** | | | | | |
| **MAPP Name** | **Number Changed** | **Number Measured** | **Z Score** | **PermuteP** | **gene symbols** |
| Notch Signaling Pathway:WP61 | 21 | 57 | 2.87 | 0.00 | APH1A|APH1B|CCND1|CIR1|DLL3|HDAC2|HEY1|ITCH|MAML1|MYC|NCOR2|NCSTN|NFKB1|NOTCH2|NUMB|PSEN1|PSEN2|SAP30|SKP1|SNW1|SPEN |
| Electron Transport Chain:WP111 | 32 | 102 | 2.50 | 0.03 | ATP5B|ATP5E|ATP5G1|ATP5H|ATP5J|ATP5J2|ATP5L|ATP5S|COX15|COX17|COX17P1|COX8A|J01415.25|MT-ATP6|MT-CO1|MT-CO2|NDUFA1|NDUFA10|NDUFA5|NDUFA7|NDUFA8|NDUFB1|NDUFB4|NDUFS3|NDUFS4|SDHD|SEC31B|SLC25A14|SLC25A5|SLC25A6|UCP2|UQCR10|UQCR11|UQCRB |
| Folic Acid Network:WP176 | 11 | 27 | 2.46 | 0.01 | ALOX5|GPX4|GSR|MT-CO1|MT-CO2|MTHFR|MTR|RP11-884K10.5|SEPW1|SEPX1|TXNRD1 |
